# Sex-related differences in cardiovascular pharmacotherapy: fiction or fact? Why can’t we see the evidence?

**DOI:** 10.1093/ehjcvp/pvaf057

**Published:** 2025-08-04

**Authors:** Juan Tamargo, Eva Delpón

**Affiliations:** Department of Pharmacology and Toxicology, School of Medicine, Universidad Complutense, Instituto de Investigación Sanitaria Gregorio Marañón, CIBERCV, Madrid 28040, Spain; Department of Pharmacology and Toxicology, School of Medicine, Universidad Complutense, Instituto de Investigación Sanitaria Gregorio Marañón, CIBERCV, Madrid 28040, Spain

**Keywords:** Women, Men, Sex, Gender, Cardiovascular drugs, Efficacy, Adverse events, Dose, Randomized clinical trials

## Abstract

There are sex-related differences (SRDs) in body composition, physiology, pharmacokinetics, efficacy, safety, and in dosage of some cardiovascular drugs. Thus, men and women may respond differently to certain drugs. However, information on SRDs in efficacy, safety, and dosage of cardiovascular drugs is scarce and their clinical relevance remains uncertain for two main reasons the traditional under-representation of women and drug efficacy and safety is not reported in a sex-disaggregated manner in randomized clinical trials (RCT). Thus, many RCTs were underpowered to analyse and detect SRDs, even if they do exist, and clinical practice guidelines (CPG) based on these RCTs recommend (with few exceptions) to treat women like men. Furthermore, women are less likely to receive CPG-recommended cardiovascular drugs (CPGRDs), present more adverse drug reactions, and may require lower doses of some drugs than men. In the era of ‘precision medicine’, this limited information should stimulate basic and clinical research to better understand the mechanisms underlying these SRDs in the efficacy and safety of CPGRDs because this represents the first step to develop a personalized pharmacotherapy. The aim of this narrative review is to analyse the reasons and consequences of the limited information on SRDs in efficacy, safety, and dosage of CPGRDs, to analyse whether the recommended doses are appropriate for women, to analyse the differences in the use of CPGRDs, and finally, to formulate recommendations to close our gaps in knowledge about SRDs and reverse the current situation to improve CVD prevention and treatment from a sex-specific perspective.

## Graphical Abstract

**Graphical Abstract:**
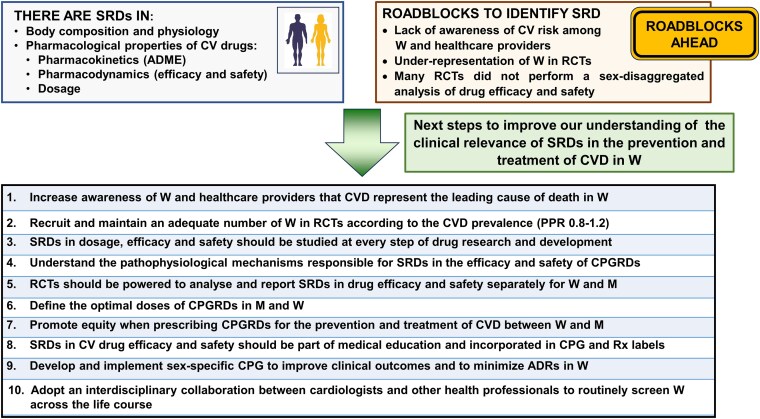
Main roadblocks to identify sex-related differences in the pharmacokinetics, pharmacodynamics (efficacy and safety), dosage of cardiovascular drugs, and main steps to improve our understanding of the clinical relevance of sex-related differences in the prevention and treatment of cardiovascular diseases in women. ADME, absorption, distribution, metabolism, and excretion; ADRs, adverse drug reactions; CPG, clinical practice guidelines; CPGRDs, clinical practice guidelines-recommended cardiovascular drugs; CV, cardiovascular; CVD, cardiovascular disease; M, men; PPR, participation-to-prevalence ratio; RCTs, randomized clinical trials; SRDs, sex-related differences; W, women.

*Anything's hard to find if you go around looking for it with your eyes shut* (Shelagh Delaney, 1938–2011)

## Introduction

Cardiovascular diseases (CVDs) remain the leading cause of death and accounts for 35% of deaths in women annually, i.e. more than all cancers combined.^1^ For decades, the risk of CVDs in women was underestimated by physicians because of the misperception that they were ‘protected’, which together with the differences in clinical presentation, and the limited awareness of the risk of CVDs among women, primary care physicians (PCPs) and cardiologists led to the underutilization of clinical practice guidelines (CPG)-recommended cardiovascular drugs (CPGRDs).^1-3^

Interestingly, there are important sex-related differences (SRDs) in the following: (i) aetiology, pathophysiology, and risk factors that affect the presentation, diagnosis, treatment, and clinical outcomes of CVDs; (ii) body composition and physiology (cyclic fluctuations in endogenous sex hormone levels during menstrual cycle/pregnancy/menopause); (iii) pharmacokinetics (absorption, distribution, biotransformation, and elimination), and (iv) pharmacodynamics (efficacy and safety) of cardiovascular drugs.^3-6^ Thus, it shouldn't be a surprise that women and man may present differences in how they respond to some cardiovascular drugs and may need different doses of these drugs.

Unfortunately, and despite its clinical relevance, the information from randomized controlled trials (RCTs) on SRDs on the efficacy, safety, and dosage of cardiovascular drugs is rather limited, sometimes controversial, and generally considered of little clinical relevance. Therefore, the role of SRDs is poorly recognised, slowly translated into changes in clinical practice, and CPG recommend (with few exceptions) the same treatment and doses for man and women despite the important differences in drug safety (see in the following), and sex-specific treatment guidelines are not considered necessary. However, in the era of precision medicine, it is difficult to understand how a personalized healthcare can be achieved without considering these SRDs. Additionally, the poor understanding of the mechanisms and clinical relevance of SRDs increase sex-related inequities in the prevention and treatment of CVDs and can result in less effective, or even harmful, treatments for women.^3,7,8^ Conversely, a better understanding of possible SRDs regarding the dosage, efficacy, and safety of CPGRDs represents the first step towards designing effective personalized cardiovascular drug therapy, reducing sex-based disparities, and achieving equitable care for women with CVDs.^7^

The objective of this brief narrative review is to discuss the reasons for the limited information available on SRDs in the efficacy, safety, and dosage of cardiovascular drugs, the clinical consequences of such differences, whether the doses of cardiovascular drugs may be different in women, the differences in CPGRD prescription rates, and to provide recommendations to close the gaps in our understanding of the mechanisms responsible for SRDs in drug efficacy and safety in order to improve CVD prevention and treatment in women. In many publications, the terms ‘sex’ (biological attributes, including physical features, chromosomes, gene expression, hormones and anatomy, categorized as female or male) and ‘gender’ (socially constructed roles, behaviours, activities, and identities that a given society considers appropriate for women, men, and gender-diverse people) are used as synonyms. Because gender identity is not confined to the sex assigned at birth, nor is it static, but can vary across societies and change over time, and few RCTs have considered sex (or gender) beyond binary variables, in this review we will refer to sex-related differences throughout.

## Methods

Guided by a professional information specialist, we searched publications on sex-related differences in cardiovascular pharmacotherapy. Search terms (medical subject headings), data sources (databases), and strategy terms used to effectively identify relevant information are summarized in Supplementary material online, *Table S1*. We also ran searches on different cardiovascular groups of drugs. We identified clinical trials, reviews, posthoc analysis, and meta-analysis published in English language with a focus on data originating from Western countries. A full-text screen was performed to assess whether studies included both men and women and if interaction for sex was tested and whether sex-stratified results were presented.

### The first obstacle: the risk of CVD is underestimated by both women and physicians

Traditionally, CVD was considered a ‘man's disease’, which contributed to the wrong perception that women were less at risk. Indeed, the first step towards personalized medicine is to increase the awareness of CVD risk among women and healthcare providers. A Women's Heart Alliance survey confirmed that only 45% of women recognized CVD as their greatest health threat; CVD was rated as the top concern by only 39% of primary care physicians (PCPs); only 22% of PCPs and 42% of cardiologists felt well prepared to assess CVD risk in women, 16% of PCPs and 22% of cardiologists implemented the American Heart Association (AHA) guidelines for CVD risk assessment in women, and 49% of PCPs and 59% of cardiologists reported that their medical training prepared them to assess female patients’ CVD risk^9^; and finally, nearly 70% of postgraduate medical trainees reported no or minimal training on sex-based medical concepts.^7^ In Canada, only 26% of PCPs and cardiologists believe they were able to effectively support women in understanding their CVD risks, and even fewer (10%–24%) felt they were effective in providing advice to women on preventing and managing CVD risk factors.^10^ Surprisingly, a report from the AHA confirmed that the awareness of CVDs to be the leading cause of death among women declined from 2009 (65%) to 2019 (44%).^7^ Therefore, there is much work ahead to increase the awareness of CVD risk among women and healthcare providers to ensure prompt diagnosis, referral, treatment, and to improve clinical outcomes.^9^ The development of educational programmes for women and healthcare providers and a better collaboration between cardiologists and other health professionals (particularly obstetricians/gynaecologists) are important steps to increase the awareness about CVD risks in women. Only when women understand that CVDs represent the main cause of death, they will be empowered to take the more appropriate measures for CVD prevention and treatment.

### The reasons for the limited evidence of SRDs in drug efficacy

Several SRDs have been described in the dosage, pharmacokinetics, and pharmacodynamics (*Table 1*) and particularly in the safety profile (*Table 2*) of some cardiovascular drugs, but they are generally considered to have little clinical relevance and, therefore, rarely translate into changes in clinical practice. What is surprising is the limited information available on the SRDs of many CPGRDs that have been on the market for decades [i.e. angiotensin-converting enzyme inhibitors (ACEIs), angiotensin receptor blockers (ARBs), mineralocorticoid receptor antagonists (MRAs), β-blockers, nitrates, antithrombotics] or that have recently been marketed and are strongly recommended in CPG.

**Table 1:** Sex-related differences in the pharmacokinetics and pharmacodynamics of guideline-recommended cardiovascular drugs

Drug class	Differences in pharmacokinetics/pharmacodynamics between women and men
Angiotensin-converting enzyme inhibitors	W were under-represented in pivotal HF trials and sex interactions were not provided HFrEF: improvement in survival appeared to be greater in M (37%) than in W (22%) ATLAS trial: M might benefit more from higher doses of lisinopril, whereas lower doses were effective in W HFrEF: W may have the lowest risk of death or HFH at half the guideline-recommended doses compared with M W with asymptomatic LV systolic dysfunction did not achieve a mortality benefit when treated with ACEI
Antiarrhythmics	Weight adjustment for loading doses of digoxin and Class I and III antiarrhythmic drugs are recommended Procainamide: higher plasma levels (30%) in W due to a lower BMI and Vd Lidocaine: significantly higher free plasma levels in W receiving OC (oestrogens reduce α1-acid glycoprotein levels)
Anticoagulants	UFH is cleared more rapidly in M than in W and W had higher heparin levels and aPTT values than M Post-MI: W treated with UFH achieve higher aPTT levels than M, and aPTT >70 s increased the risk of death, stroke, bleeding, and reinfarction ACS (ESSENCE and TIMI 11B trials): significantly greater benefit of enoxaparin over UFH in W, but not in M Higher anti-Xa activity of dalteparin in W than in M (*P* < 0.001) Noncardiac arterial procedures: W reached significantly higher levels of anticoagulation than M Bivaluridin (REPLACE-2, ACUITY, HORIZONS-AMI trials): more pronounced benefit in reducing 1-year mortality in W than in M
Antiplatelets (except aspirin)	Exposure to ticagrelor and AR-C124910XX is ∼40%–60% higher W than in M, but no dose adjustment is recommended Patients undergoing PCI (29 RCTs; *n* = 99 591; 25.2% W): W present a higher risk of MACCE and bleeding events when newer P2Y12 inhibitors are used MI (5 RCTs; *n* = 79 613; 30% W): clopidogrel-reduced MACE was driven by a reduction in MI in W and by a reduction in MI, stroke and all-cause mortality in M GP IIb/IIIa inhibitors in NST-ACS (6 RCTs; *n* = 31 492; 35% W): treatment benefit in M but not in W (*P* < 0.0001); no SRDs when patients were stratified according to troponin levels The net clinical benefit of antiplatelets tends to be generally smaller in W than in M
Angiotensin receptor blockers	W were under-represented in hypertension and HF trials and sex-specific analysis were not provided W with hypertension or HF are more likely to receive ARBs HFrEF: W may have the lowest risk of death or HFH at half the guideline-recommended doses compared with M HEAAL trial: M appeared to benefit more from high doses of losartan; W responded similarly to low or high doses
Aspirin	Greater oral bioavailability, larger Vd, slower Cl due reduced glycine conjugation and longer half-life in W than in M Higher prevalence of aspirin resistance in W than in M because of increased baseline platelet reactivity Aspirin may be less effective at inhibiting platelet aggregation in W than in M with a history of ischaemic stroke or TIA MI prevention (23 trials; *n* = 113 494; 50% W): trials recruiting predominantly M showed the largest reduction (38%) in nonfatal MI, while trials recruiting mostly W failed to demonstrate any benefits
Beta-blockers	Higher exposure of CYP2D6-dependent β−blockers (carvedilol, metoprolol, nebivolol, propranolol) in W due to higher oral bioavailability, lower Vd, and slower metabolism. Post-MI: propranolol exposure was significantly higher in W than in M Larger decrease in heart rate and blood pressure, in W than in M on similar doses Similar metoprolol exposure after 25 mg in elderly W, 50 mg in elderly M, and 100 mg in adult M; reduce the dose in W (50%) RCTs were underpowered to assess SRDs in patients with HFpEF or HFrEF HFrEF: W may have the lowest risk of death or HFH at half the CPG-recommended doses compared with M CIBIS II trial: bisoprolol significantly decreased all-cause, CV, and pump failure deaths in W than in M with HFrEF
Calcium channel blockers	W present faster Cl and lower exposure of nifedipine and verapamil, due to the lower BMI, higher activity of CYP3A4 and lower activity of P-gp Amlodipine and verapamil clearance decreases in W with aging; amlodipine produces more pronounced BP reduction in elderly W. However, major RCTs did not found SRDs in hypertensive patients HOT trial: fewer MI in the lowest diastolic BP target group (<80–85 mmHg) in W, but not in M
Digoxin	Higher SDC in W due to reduced Vd and lower renal Cl HFrEF: pivotal trials (DIG, PROVED, RADIANCE) did not perform sex-specific analysis
Diuretics (thiazide and loop)	No study has evaluated possible SRDs in patients with hypertension of HF Lower Cl and higher exposure (40%–50%) of torasemide, but no dose adjustment is recommended Thiazides improve stroke and MACE in W and total and specific mortality, all coronary events, all strokes, and MACE in M
Direct oral anticoagulants	Dabigatran exposure is ∼30% higher in W but no dose adjustment is indicated Major RCTs were not designed to conduct sex-specific analyses, but doses were adjusted by body weight and renal function, which implies some correction in W ARISTOTLE trial: among patients with AF and previous history of stroke/TIA, W have a lower risk of recurrent stroke and all-cause and CV death than M (both *P* < 0.0001) Nationwide Taiwanese cohort: greater risk reduction of ischaemic stroke W as compared with M (*P* = 0.04) Meta-analysis of 4 RCTs (37% W): M were more protected from stroke/SEE and W from major bleeding events AF (5 RCTs, 66 389 patients, 37.8% W): W treated with DOAC were at higher risk of stroke and systemic embolism than M Compared with warfarin, DOAC use was associated with a lower risk of ICH and all-cause mortality in W but not in M Acute VTE: in RCTs all-cause mortality was not reported by sex
ET-1 receptor antagonists	Six RCTs (*n* = 1130; 26% W): W experienced significantly greater benefits in terms of change in 6 MWD than M
GLP-1 receptor agonists	T2D (7 CVOTs; *n* = 56 004): GLP-1RA significantly reduce MACE in M, but not in W, as compared with placebo T2D: greater effectiveness of GLP-1RA in W than M
Isosorbide dinitrate	Higher drug exposure in W probably due to their lower body weight
Isosorbide dinitrate/hydralazine	Isosorbide dinitrate/hydralazine seemed to have a more pronounced mortality benefit in women, but there was no significant treatment interaction by sex
MRA^a^	Serum aldosterone levels are directly related with LV concentric remodelling in W but not in M AA W (but not AA M, or white W) on spironolactone had lower BP and less uncontrolled hypertension HFpEF: in a non-prespecified subanalysis of TOPCAT-Americas, spironolactone reduced all-cause mortality in W, but not in M AMI and LV dysfunction: a trend towards greater benefit for 30-days all-cause mortality in W treated with eplerenone
Sacubitril-valsartan^a^	HFpEF (PARAGON trial, 51.7% W): sacubitril/valsartan significantly reduced CV and HFH in W but not in M (P interaction = 0.017) due to a greater reduction in HFH
SGLT2 inhibitors	EMPEROR-Reduced trial: larger benefit with empagliflozin vs. placebo for the composite of CV death or HFH in W (HR 0.59) than in M (HR 0.80) HF (5 RCTs; *n* = 21 948; 35.7% W): SGLT2 inhibitors reduced the composite of CV death and HFH, but the benefit was less pronounced in W T2D (3 CVOT; *n* = 34 322): SGLT2I reduced MACE in M, but not in W Meta-regression analysis (26 RCTs; *n* = 22 256; 58% W): significant reduction in all-cause mortality when the percentage of females was ≤50% but not when it was >50%
Statins	Statins have not been adequately tested in W, especially in primary prevention trials W are less likely to receive high-intensity statin therapy and to reach LDL-C levels <100 mg/dL compared with M
Thrombolytic drugs	Pooled analysis of three RCTs (*n* = 2179; 44% W): W with acute ischaemic stroke benefit from rtPA more than M Non-ST-elevation ACS undergoing PCI: the benefit with abciximab is greater in M than in W STEMI undergoing PCI: abciximab significantly reduced ischaemic events and 30 day and 1-year mortality in W, but not in M Contradictory results regarding the risk of morbidity and mortality in W and M in the setting of thrombolysis
Warfarin	Enhanced dose–response to warfarin in W, due to lower mean BMI and SRDs in warfarin metabolism via P450 enzymes Significantly higher free plasma levels in W; they require lower doses of warfarin than M to maintain the INR Meta-analysis (6 RCTs; *n* = 63 602; 28% W): W with AF on warfarin were at higher residual risk of CVA/SE than M (*P* = 0.001) W treated with warfarin spend more time outside the therapeutic range than M

References are presented in Supplementary material online, *Table S2*.

AA, African American; ACEI, angiotensin-converting enzyme inhibitors; ACS, acute coronary syndrome; ADRs, adverse drug reactions; AF, atrial fibrillation; AMI, acute myocardial infarction; aPPT, activated partial thromboplastin time; ARB, angiotensin AT1 receptor blockers; BMI, body mass index; BP, blood pressure; Cl, clearance; CPG, clinical practice guidelines; crCl, creatinine clearance; CV, cardiovascular; CVA/SE, cerebrovascular accident/systemic embolism; CVOT, cardiovascular outcome trial; CYP, cytochrome P450; DOAC, direct oral anticoagulants; ET-1, endothelin-1; GLP-1RAs, glucagon-like peptide-1 receptor agonists; HF, heart failure; HFH, heart failure hospitalization; HFpEF/HFrEF, heart failure with preserved/reduced ejection fraction; HR, hazard ratio; ICH, intracerebral haemorrhage; INR, international normalised ratio; LDL-C, low-density lipoprotein-cholesterol; LV, left ventricular; M, men; MACCE, major adverse cardiac and cerebrovascular events; MACE, major cardiovascular events; MI, myocardial infarction; MRA, mineralocorticoid receptor antagonists; 6MWD, 6-minute walking distance; NST-ACS, non-ST segment elevation ACS; OC, oral contraceptive; PCI, percutaneous coronary intervention; PD, pharmacodynamic; P-gp, P-glycoprotein; RCTs, randomized clinical trials; rtPA, recombinant tissue plasminogen activator; sCr, serum creatinine; SDC, serum digoxin concentrations; SEE, systemic embolism; SGLT2, sodium-glucose cotransporter-2; SRDs, sex-related differences; STEMI, ST-elevation MI; TD, thiazide diuretic; T2D, Type 2 diabetes; TIA, transient ischaemic attack; UFH, unfractionated heparin; Vd, volume of distribution; VTE, venous thromboembolism; W, women.

^a^They can cause foetal harm and teratogenic effects: avoid in W during childbearing years; contraindicated during pregnancy.

**Table 2:** Sex-related differences in adverse drug reactions of cardiovascular drugs

Drug class	Differences in adverse drug reactions between women and men
Angiotensin-converting enzyme inhibitors^a^	Cough, dysgeusia, skin rash, increase in sCr, gastrointestinal upset and angioedema are more frequent in W. W are less persistent on ACEI than M in all age groups. W discontinue ACEI due to cough, M because of hypotension
Antiarrhythmics	W present a higher risk for developing QT-related arrhythmias; up to two thirds of torsades de pointes occurred in W Procainamide-induced lupus erythematosus is more common in W
Anticoagulants	Increased risk of bleeding in W than in M. W seemed more prone to be hospitalized with anaemia Low molecular weight heparin-induced thrombocytopenia is more frequent in W than M
Antiplatelets	Increased risk of bleeding in W than in M. Bleeding remain higher in W after dose adaptation to BMI and sCr W are more likely to receive higher doses of GP IIb/IIIa inhibitors and are at higher risk of bleeding than M Cangrelor increases the risk of GUSTO moderate bleeding in W but not in M (*P*_interaction_ = 0.04)
Angiotensin receptor blockers^a^	No SRDs in hyperkalaemia, or hypotension but the incidence of angioedema was higher in M In W, replace ACEI by ARB to reduce the risk of ADRs W appear to be more persistent to ARB than to ACEI, probably because they produced less ADRs
Aspirin	Increased bleeding risk in W than in M The risk of hospitalizations for bleeding due to salicylates and NSAID-related ulcer complications is higher in M
Beta-blockers	ADRs caused by CYP2D6-dependent β-blockers are more frequent in W; they may benefit from lower doses than M Metoprolol and propranolol produce a greater reduction in blood pressure and heart rate during exercise in W than in M
Calcium channel blockers	Peripheral oedema, potentially leading to decreased adherence is more frequent in W Flushing, headache and ankle oedema are more common in W, particularly in elderly W
Digoxin	Higher mortality in W than in M when SDC ≥1.2 ng/mL (*P*_interaction_ = 0.034). SDC ≤0.9 ng/mL are recommended in W with HFeEF to reduce mortality W are at higher risk of cardiac hospitalizations
Diuretics (thiazide and loop)	W experience more hypo-osmolarity and hospitalizations due to hyponatremia and hypokalaemia W are less likely to experience hyperuricaemia and gout in response to thiazide diuretics than M
Direct oral anticoagulants	AF: major bleeding was less frequent in W than in M VTE (17 RCTs, *n* = 25 789; 37.6–43.7% W): W had a higher incidence of major bleedings plus clinically relevant minor bleedings than M
GLP-1 receptor agonists	W experienced significantly higher incidence of ADRs than M
Isosorbide dinitrate	Doses should be based on body weight
Isosorbide dinitrate/ hydralazine	Headache, dizziness and systemic lupus erythematosus are more common in W
Ivabradine	RCTs did not report sex-specific ADR data. Vigibase: more non-serious ADRs in W
Mineralocorticoid receptor antagonists^a^	HFrEF: higher drug discontinuation due to hyperkalaemia, renal impairment, gynaecomastia in M than in W. Eplerenone: produced greater increases in sCr in W HFrEF: only one out of 18 RCTs reported sex-specific ADR data HFpEF: start MRA if sCr <2.5 mg/dL in M or <2.0 mg/dL in W, and serum potassium <5.0 mEq/L
Sacubitril-valsartan^a^	W are more likely to develop hypotension and angioedema. ADRs were not analysed by sex in RCTs
SGLT2 inhibitors	Higher rates of diabetic ketoacidosis and genitourinary infections in W and acute renal failure in M
Statins	The risk of statin-induced myalgia/myopathy and new-onset diabetes is highest in older W with low BMI W are more likely to discontinue statin therapy because of an ADR than M
Thrombolytic drugs	AMI: W present a higher risk of death, reinfarction, stroke, and haemorrhagic stroke than M Bleeding risk is only partly reduced after dose adjustments for BMI and sCr, suggesting the involvement of PD mechanisms
Warfarin	Higher risk of bleeding in W than M W need less warfarin per week than M; dose requirements decrease greatly with age, particularly in elderly W

References are presented in Supplementary material online, *Table S3*.

ACEIs, angiotensin-converting enzyme inhibitors; ADRs, adverse drug reactions; AF, atrial fibrillation; AMI, acute myocardial infarction; ARB, angiotensin AT1 receptor blockers; BMI, body mass index; CYP, cytochrome P450; DIG, Digitalis Investigation Group; GLP-1, glucagon-like peptide-1 receptor agonists; GP, glycoprotein; GUSTO, Global Utilization of Streptokinase and t-PA for Occluded Coronary Arteries; HFpEF/HFrEF, heart failure with preserved/reduced ejection fraction; M, men; MRA, mineralocorticoid receptor antagonists; NSAID, non-steroidal anti-inflammatory drugs; RCTs, randomized clinical trials; sCr, serum creatinine; SDC, serum digoxin concentrations; SRDs, sex-related differences; VTE, venous thromboembolism; W, women.

^a^They can cause foetal harm and teratogenic effects: avoid in W during childbearing years; contraindicated during pregnancy.

This scarce information is the result of the following:

#### 1. Historical under-representation of women in RCTs

Generally making up only ≈30% of enrolled patients, i.e. women were represented at a lower proportion relative to their prevalence in the underlying population (*Table 3*). This lower representation is also evident as investigators, first authors, and members in the executive committees of RCTs and independently associated with a lower recruitment of women as trial participants.^7^ Prevalence-corrected estimates for the participation of women can be calculated using the participation-to-prevalence ratio (PPR). A PPR between 0.8 and 1.2 reflects similar representation of women in the RCT and disease population, while a PPR <0.8 or >1.2 indicates that women were under-represented or over-represented in the RCT, respectively.^11^

**Table 3:** Representation of women and sex-specific analysis of drug efficacy and safety in cardiovascular drug trials

Study	Results
**A. Representation of women in RCTs**	
SBA in the Cochrane reviews	In 258 reviews, W comprised only 27% of the pooled population
NHLBI-sponsored CV RCTs from 1997 to 2006	In 19 trials, mean enrolment of W was 27%
2007 AHA guidelines for CVD prevention	In 156 trials (*n* = 801 198), W representation was highest in hypertension (44%), DM (40%) and stroke (38%), lowest in HF (29%), CAD (25%) and hyperlipidaemia (28%) trials
RCTs published in nine prominent medical journals in 2009	Median enrolment of W in 56 RCTs was 37%; in nine studies, less than 20% of W were enrolled
RCTs cited in the ACC/AHA guidelines for AF (2006), HF (2009), and ACS (2011)	In 653 trials (*n* = 1 336 922), W represented 33% in AF and 29% in ACS and HF trials, which was lower than that of US registries of AF (55%), ACS (42%) and HF (47%)
Publications in the three leading medical journals (1997–2009)	325 CV trials recruiting more than 1.2 million patients. Enrolment rates of W: 27% in trials of CAD, 27% in HF and 31% in arrhythmia. Between 1997 and 2009 W enrolment rate increased from 27% to 32%
ESC-HF-LT registry	W were only 28.8% of the registry patients (*n* = 12 440)
RCTs and observational studies published in 11 peer-reviewed journals in 2013	In 264 RCTs and observational studies, W represented 29% of the participants
RCTs in HF trials published between 1985 and 2016	Analysis of 22 RCTs (*n* = 72 898). In HFrEF trials W represented 20%–32% of participants; in HFpEF trials, W represented 40%–60% of participants
Most cited RCTs of Cardiology between 1996 and 2015	The median percentage of W in trials was 28.6% (22.2–40.5%) and only 14% of trials (*n* = 70) enrolled >50% W
Review of sex differences in cardiovascular epigenetics	In 75 publications, 24 (86%) studied males, and 4 (14%) studied females
RCTs supporting FDA approval of 36 drugs from 2005 to 2015	W represented 34% of 174 709 participants. The PPR by CVD was ≥0.8 for AF (0.8–1.1), hypertension (0.9), and PAH (1.4) trials; PPR was <0.8 for HF (0.5–0.6), CAD (0.6), and ACS/MI (0.6) trials
Review of HF trials published between 2001 and 2016	118 trials (*n* = 215 508). HFpEF trials enrolled a higher proportion of W (56%) compared with HFrEF (24%) or AHF (32%). Corresponding weighted proportions in epidemiologic studies were 62%, 29%, and 50%, respectively.
Pivotal RCTs with DOAC in AF	In 5 RCTs (*n* = 66 389 patients), W represented 37.8% of the participants
CV RCTs published in high-IF journals between 1986 and 2015	In 598 trials (*n* = 2 965 314), W enrolment varied from 37% for non-CAD, 30% for CAD, 28% for HF and 28% for arrhythmia trials, significantly lower than the expected proportion in disease populations. W enrolment increased over time from 21% to 33%
RCTs cited in the ESC 2016 HF guidelines	Across 23 trials (*n* = 101 564 participants) W represented 25.2% of participants
CV clinical trials published in six major medical and CV journal over two decades	In 616 RCTs published between 2000 and 2015, the proportion of W in CV trials increased from 28.5% to 35.8% (*P* < 0.001), but varied among different CVD: from 25.5% in CAD and 27.3% in HF, to 51.9% in hypertension
Pivotal RCTs supporting FDA approval of 35 new cardiometabolic drugs (2008–2017)	W represented 36% of participants (*n* = 296 163). They were under-represented in CAD (PPR 0.52), HF (0.58), and ACS 0.68) trials, and overrepresented in PAH trials (1.35). W enrolment did not increase over this study period
Systematic review of 740 CVD trials (2010–2017)	W represented 38.2% of participants (*n* = 862 652). W were reasonably represented in hypertension (PPR 0.82) and PAH trials (1.33), but under-represented in arrhythmia (0.78), CAD (0.67), stroke (0.73), ACS (0.66) and HF (0.48) trials
RCTs with lipid-lowering drugs (1990–2018)	In 60 RCTs (*n* = 485 409; 28.5% W), W were under-represented in lipid RCTs of DM (PPR, 0.74), HF (0.27), stable CAD (0.48), and ACS (0.51). W enrolment increased from 1990–1994 vs. 2015–2018 (19.5% vs. 33.6%)
Inclisiran in patients with hypercholesterolaemia	ORION-10 and ORION-11 trials (*n* = 3184): only 935 patients were W (29%, PPR 0.63)
HFrEF trials published in high-IF journals (2000–2019)	In 317 RCTs (*n* = 183 097), W represented 25.5% of patients. W were underenrolled in 71.6% of trials; 40.5% of trials enrolled ≤20% of W. Enrolment did not increase significantly between 2000–2019.
Representation in stroke trials (1990–2020)	Among 281 trials (*n* = 588 887), W were under-represented (37,4%), particularly in trials of intracerebral haemorrhage (PPR 0.73)
Sacubitril–vasartan in HFrEF trials	Meta-analysis of 8 RCTs (*n* = 8981): only 32.2% were W
Antiplatelet and anticoagulant landmark trials (1988–2019)	39 trials (*n* = 447 496) with aspirin, clopidogrel, ticagrelor, prasugrel, warfarin, apixaban, and rivaroxaban. W represented 30.1% of participants
HF trials (1986–2020)	In 30 RCTs (*n* = 118 282 participants), W represented 24.1% of participants
HF RCTs published between 2000 and 2020	In 224 HF RCTs (*n* = 228 801) published in journals with an IF ≥10, W represented 28.2% of patients
US trials of common vascular diseases (2008–2020)	97 trials (*n* = 41 622). W represented 27.5% of patients and were under-represented for all studied conditions: CAS (PPR 0.73), PAD (0.65), TAA/AAA (0.59) and TBAD. W participation did not improve since 2008
Antihypertensive trials until May 2020^75^	In 2046 studies with the five major antihypertensive drugs (*n* = 1 348 172), W represented 38.1% of patients
Late-Breaking CV RCTs presented at the 2021 ACC/AHA/ESC meetings	In 68 trials, inclusion of W ranged from 0% to 71%, with a mean of 35.2%
2023 ACS guidelines of the ESC	W represent 20%–30% of patients in RCTs of ACS
Publications cited in the 2019 ESC guideline on chronic coronary syndromes	In 108 articles published between 1991 and 2019 (*n* = 1 613 000), only 26.8% (432 284) were W
SGLT2 inhibitors and HF RCTs published between 2018–2024	In 43 RCTs (*n* = 27 703), the overall proportion of W enrolled in the studies was 35.6%
**B. Sex-specific analysis of drug efficacy and safety**	
Cochrane Reviews related to treatment of CVD	In 196 trials, only 33% performed a SBA of outcomes. In trials that performed a SBA, 20% reported significant sex-related differences in CV-related outcomes
2007 AHA guidelines for CVD prevention	In 156 RCTs, sex-specific results were discussed in only 31% of primary trial publications
RCTs published in nine prominent medical journals in 2009	In 86 trials, 75% of the studies did not report any outcomes by sex
SBA in 38 Cochrane systematic reviews on CVD from 2001 to 2007	Only two reviews reported any sex research gaps; only one quarter included a rationale as to why any subgroup analyses by sex were or were not completed. None met all the appraisal tool criteria.
RCTs published in high-IF journals (2008–2013)	In 57 randomly selected federally-funded RCTs (43% W), the minority of trials (22%) that did analyse sex differences did not discuss or reflect upon these, or dismissed significant findings
RCTs performed in Canada in 2013–2014	100 RCTs: 6% performed a SBA; 4% reported sex-disaggregated data; one discussed the implications of the findings for clinical practice
Analysis of 133 CaRs and 555 CoRs from 2016 to 2017	In the results section, less than 30% of reviews reported on sex. 37% of CaRs and 75% of CoRs provided a descriptive report of sex and 63% and 25% reported analytic approaches for exploring sex (subgroup analyses or presented sex-disaggregated)
Review of sex differences in cardiovascular epigenetics	In 75 publications only 13 (17%) stratified some of their data according to sex
Sex-sensitive review behind current HF CPG	HF RCTs cited in CPG in Europe, US, and Canada were not powered to detect SRDs, to test the benefit in W, or to identify a drug that would only be effective in W
RCTs cited in the ESC 2016 HF guidelines	Of 155 trials (*n* = 153 945), only 11 (7%) reported sex-specific ADR data
	23 RCTs (*n* = 101 564; 25% W): 48% reported sex-specific efficacy data, and only two studies (9%) presented sex-specific information about ADRs
RCTs with lipid-lowering therapies (1990–2018)	In 60 RCTs (485 409 participants, 28.5% W), only 53.0% of these trials reported outcomes by sex, with no significant improvement over time
	In 60 RCTs (*n* = 485 409; 28.5% W), W were under-represented in lipid RCTs of DM (PPR 0.74), HF (0.27), stable CAD (0.48), and ACS (0.51). W enrolment increased from 1990–1994 vs. 2015–2018 (19.5% vs. 33.6%)
Novel cardiometabolic drug approvals from 2008 to 2017	143 trials (*n* = 296 163): 57 CV and 86 DM (296 163 participants; 36% W). W were under-represented in trials of CAD (PPR 0.52), HF (0.58), and ACS (0.68), but overrepresented in PAH trials (1.35)
HF RCTs published in nine high-IF journals between 2008 and 2017	Of 261 HF trials, 107 (41%) reported subgroup analyses, credibility of subgroup claims was generally low across all strengths of claims
RCTs cited by the 2018 ESH-ESC guidelines	33 RCTs with >1000 subjects (305 249 participants; 44, 4% W). Only 11 trials reported the results according to sex; 7 did it in a subsequent publication devoted to planned subgroup analysis
RCTs presented between 2010 and 2017 in the ESC/AHA/ACC sessions and published in high-IF journals	In 63 RCTs, sex-specific efficacy endpoints were reported for 34.5% of trials in 2010 and 23.5% in 2017; sex-specific safety outcomes in 11.1% and 8.6%, respectively
Sex-specific reporting in HF RCTs published between 2000 and 2020	In 224 RCTs (*n* = 228 801), no trial reported sex-disaggregated screening, consent or withdrawal rates or sex-specific ADRs; 75 RCTs (33.4%) presented sex subgroup analysis, and 63 (28.3%) reported sex-treatment interaction
Trials on antihypertensive drugs until May 2020	Among 2046 studies (*n* = 1 348 172 adults; 30.1% W), only 75 (3.7%) studies performed sex stratification, and this was the highest between 2011 and 2020 (7.2%).
Late-Breaking CV RCTs presented at the 2021 ACC/AHA/ESC meetings	The average PPR was 0.76 for all trials, but varied based on subspecialty, with a statistical difference between interventional cardiology and HF (0.65 vs. 0.88).
Acute care trials published in high-IF journals	88 trials (75 ICU and 13 cardiology trials; *n* = 111 428; 34.2% W). Only 23 (26.1%) trials reported a SBA
References cited in the five major CPG of hypertension	Among 331 trials, 81% reported the sex of participants, and 22% a PPR of 0.8–1.2; 3% of trials stratified baseline characteristics by sex, and 20% considered sex during analysis through statistical adjustment or stratification. Only 0.6% stratified ADRs by sex.
Publications cited in the 2019 ESC guideline on chronic coronary syndromes	Of 108 trials published between 1991 and 2019, only three incorporated sex-sensitive designs. Posthoc, sex-specific analyses were found only in 13% of the publications

References are presented in Supplementary material online, *Table S4*.

ACC, American College of Cardiology; ACS, acute coronary syndrome; ADRs, adverse drug reactions; AF, atrial fibrillation; AHA, American Heart Association; AHF, acute heart failure; CAD, coronary artery disease; CaRs, Campbell reviews; CAS, carotid artery stenosis; CoRs, Cochrane reviews; CPG, clinical practice guidelines; CV, cardiovascular; CVD, cardiovascular disease; DM, diabetes mellitus; DOAC, Direct oral anticoagulants; ESC, European Society of Cardiology; ESC-HF-LT, European Society of Cardiology-Heart Failure Long-Term Registry; ESH, European Society of Hypertension; FDA, US Food and Drug Administration; HF, heart failure; HFrEF/HFpEF, heart failure with reduced/preserved ejection fraction; ICU, intensive care unit; IF, impact factor; M, men; MI, myocardial infarction; NHLBI, National Heart, Lung, and Blood Institute; PAD, peripheral arterial disease; PAH, pulmonary arterial hypertension; PPR, participation-to-prevalence ratio (a PPR of 0.8–1.2 suggests an adequate representation of W in trials relative to disease population; a PPR <0.8 under-representation, and a PPR >1.2 over-representation). RCTs, randomized clinical trials; SBA, sex-based analysis; SGLT2: SGLT2: sodium-glucose cotransporter-2; SRDs, sex-related differences; TAA/AAA, thoracic/abdominal aortic aneurysms; TBAD, type B aortic dissections; W, women.

In the 156 RCTs cited by the 2007 women's prevention guidelines, representation of women was highest among trials in hypertension (44%), diabetes (40%), and stroke (38%) and lowest for heart failure (HF) (29%), coronary artery disease (CAD, 25%), and hyperlipidaemia (28%).^12^ However, women accounted for 53%, 50%, 51%, 49% and 46% of all individuals with hypertension, diabetes, HF, hyperlipidaemia, and CAD, respectively. A 2014 study examined women representation in 653 trials cited in the 2009–2011 American College of Cardiology (ACC)/AHA guidelines for atrial fibrillation (AF), HF, and acute coronary syndrome (ACS).^13^ Women representation was 33% in AF and 29% in ACS and HF trials, which is lower than that of US registries (55%, 42% and 47%, respectively). In a review of 589 RCTs published in high-quality journals between 1986 and 2015, mean women enrolment in pharmacological trials was 31%, but decreased from 37% for non-CAD vascular trials to 30% for CAD trials and 28% for HF and arrhythmia trials.^14^

In an analysis of trials supporting U.S. Food and Drug Administration (FDA) approval of 36 drugs from 2005 to 2015, women represented 34% of the population. The calculated PPR by CVD area was within or above the desirable range for pulmonary arterial hypertension (PAH, PPR 1.4), AF (PPR 0.8–1.1), and hypertension (0.9), but the PPR was <0.8 in HF, CAD, and ACS trials, where women represented only 24%, 24% and 28% of participants, respectively.^11^ In another systematic review of 740 trials registered in ClinicalTrials.gov supporting the approval of cardiovascular drugs between 2010 and 2017, women represented 38.2% of the population,^15^ and in an analysis of pivotal cardio-metabolic RCT, which supported FDA approval of 35 new molecular entities between 2008 and 2017, women represented 36% of participants.^16^

Despite women comprise ∼50% of all HF patients, they represented only 28.8% of patients in the ESC-HF-LT registry (*Table 3*). In a systematic search of 118 HF trials published between 2001 and 2016, heart failure with preserved ejection fraction (HFpEF) trials enrolled a higher proportion of women (56%) than HFrEF (24%) or acute HF (32%) trials (both *P* < 0.001). Again, these percentages were lower than the corresponding weighted proportions of women in epidemiologic studies (62%, 29%, and 50%, respectively).^17^ Importantly, women represented 20%–32% of the population in HFrEF landmark RCTs performed with ACEI, ARB, MRA, sacubitril–valsartan, and β-blockers which were the basis for the present recommendations of CPG.^6,18^ In 281 stroke trials recruiting 588 886 participants, women represented 37.4% of the population (mean PPR 0.84 for the total population and 0.73 in intracerebral haemorrhage trials)^19^ and in recent pivotal RCTs investigating the effects of sodium-glucose cotransporter-2 inhibitors (SGLT2I) in patients with HFrEF or direct anticoagulants in patients with AF, where women only represented 35%–38% of the participants (*Table 3*). Finally, underenrolment of women persists for CVD with higher prevalence [HFpEF, myocardial infarction (MI) associated with non-obstructive coronary arteries, spontaneous coronary artery dissection, fibromuscular dysplasia, stress-induced cardiomyopathy (Takotsubo Syndrome)] or unique considerations (peripheral arterial disease and abdominal aortic aneurysms) in women.^5^

#### 2. Many cardiovascular RCTs do not report drug efficacy and safety in a sex-disaggregated manner

(see *Table 3*), which severely limits the interpretation and generalizability of RCTs findings to women as well to men and sex-specific scientific evidence. In the 2007 women's prevention guidelines, sex-specific results were discussed in only 31% of primary trial publications^12^ and in a review of 56 federally-funded RCTs published in nine high-impact medical journals in 2009 (37% women), 75% of trials did not report any outcome by sex, included sex as a factor in modelling or provided a rationale for disregarding sex as a potential influence.^20^ In 100 Canadian RCTs, only 6% conducted a subgroup analysis across sex and 4% reported sex-disaggregated data.^21^ In another review of 224 HF RCTs published between 2000 and 2020 in high-impact journals (28.2% women), no RCT reported sex-specific screening, consent, withdrawal rates, or adverse effects and less than one-third of trials reported a sex subgroup analysis of the treatment effect on the primary outcome.^22^ Similarly, in another 88 acute care trials (75 in intensive care units, 13 cardiology trials; 34.2% women) published in leading medical journals in 2014, 2018, and 2020, women represented 37% of participants and only 23 (26%) reported a sex-based analysis, but both women representation and sex-based analysis did not improve over time.^23^ Furthermore, in a random sample of 38 Cochrane systematic reviews on CVD, only two reported any sex research gaps and one quarter the rationale as to why any subgroup analyses by sex were or not completed.^24^ Finally, in an analysis of 133 Campbell reviews and 555 Cochrane reviews, <30% of the reviews reported on sex in the results section, and only 14% of Campbell and 15% of Cochrane reviews reported on sex in the discussion section.^25^

##### 2.1. Sex analysis in the RCTs supporting guideline recommendations

The 2011 AHA guidelines for the prevention of CVD in women provided recommendations for the secondary prevention of MI and HF, but no sex-treatment interactions were analysed in the quoted RCTs.^26^ Of 108 trials, published between 1991 and 2019 (1.6 million participants, 26.8% women), quoted to support the recommendations in the 2019 European Society of Cardiology (ESC) guidelines on chronic coronary syndromes, only three studies incorporated sex-sensitive designs, five contained sex-related statements and none were sex-specific, and the term sex did not appear in 84% of the publications.^27^ Another review of HF clinical guidelines in Europe, United States, and Canada based on RCTs which often include ≤30% of women concluded that they did not reflect the sexual dimorphism and trials were not powered to detect sex–drug interactions, test the benefit in the subgroup of women, or identify a drug that would only be effective in women.^18^ Other study compared sex-specific reporting of efficacy and safety outcomes for trials of cardiovascular drugs presented at the major clinical sessions of the ESC/AHA/ACC before (2010) and after (2017) publication of statements of these societies emphasizing the importance of sex-specific reporting of RCT data.^28^ Sex-specific efficacy endpoints were reported for 34.5% and 23.5% and sex-specific safety outcomes for 11.1% and 8.6% of RCTs presented in 2010 and 2017, respectively.

Furthermore, an analysis of 331 trials informing the five major hypertension CPG on the treatment of arterial hypertension, 22% reported a PPR of 0.8–1.2, 3% stratified baseline characteristics by sex, and 20% considered sex during analysis through statistical adjustment or stratification and although 32% of studies reported adverse drug reactions (ADRs), only 0.6% stratified ADRs by sex.^29^ In another analysis of 2046 studies (1 348 172 patients, 38.1% women) evaluating the effect of antihypertensive drugs until May 2020, only 75 (3.7%) performed sex stratification.^30^ These results are not a surprise, because despite the relatively higher participation of women in antihypertensive trials, most RCTs supporting CPG recommendations were published before 2000, did not provide sex-stratified findings, and possible SRDs in pharmacokinetics were unavailable for drugs approved prior to this date in the online FDA database.^31^

It is disheartening to confirm that despite the specific recommendations and guidelines from administrations and international cardiology societies (ESC, AHA, ACC), representation of women fell below a PPR of 0.8 for trials in HF, CAD, and ACS, and knowledge gaps on SRDs in the efficacy and safety of some cardiovascular drugs result from the absence of sex-specific data reporting in many pivotal RCTs.^28^ In fact, sex-specific analysis of drug efficacy was performed in <30% of the RCTs and <10% stratified ADRs by sex and no significant improvement has been made in recent years. Nevertheless, most CPG for CVD conclude that there are no SRDs in the efficacy and safety and recommend that men and women should receive the same doses of cardiovascular drugs. As RCTs are the most important source of evidence for CPG recommendations, improvement in reporting of sex-specific data is critical to provide prescribers with solid sex-specidic recommendations for the clinical daily practice. Thus, regulatory agencies, funding bodies, and scientific societies and journals should require researchers to present the results of RCTs on the efficacy and safety of cardiovascular drugs stratified by sex so that sex-specific recommendations can be made in the future

#### 3. Clinical consequences

Translation of evidence from RCTs into clinical practice occurs when the proportion of women nearly equals the prevalence of the disease in women (PPR between 0.8 and 1.2). Thus, the lower participation of women and the limited sex-specific analysis and reporting of drug efficacy and safety in cardiovascular RCTs have important clinical consequences that are summarized in *Table 4*.^11,18^

**Table 4:** Consequences of women under-representation and the lack of sex-specific analysis and report of cardiovascular drug efficacy and safety in RCTs

1. Severely limited our ability to recognize the existence SRDs even if they exist and to understand their clinical relevance
2. RCTs where the inclusion of W does not mirror the prevalence in the general population (PPR <0.8) have important consequences:
They suffer from sample selection bias as W are considered as a subgroup
Do not reproduce real-world practice and are not representative of the population to treat
Results cannot be extrapolated to the population that was not adequately represented, i.e. women
Although many trials concluded that there are no SRDs, the efficacy and safety of some CV drugs remains uncertain in W
3. CPG based on RCTs recruiting mainly middle-aged men frequently conclude that there are no SRDs
Recommend (with few exceptions) the same treatment and doses for both man and women despite the important SRDs in drug safety
Recommendations may be more suitable for M, leading to suboptimal care for W with CVD
Clinicians do not have evidence-based information to guide W healthcare, potentially leading to suboptimal outcomes, or alternatively, exposing them to frequent adverse drug reactions
4. The limited information on SRDs is the result of RCTs unpowered to detect such differences, rather than a true absence of differences
Hinders the ability to develop personalized sex-specific strategies for the prevention and treatment of CVD and the formulation of policies/programmes to reduce inequities in healthcare in W with CVD

References are presented in Supplementary material online, *Table S5*.

CPG, clinical practice guidelines; CV, cardiovascular; CVD, cardiovascular disease; M, men; PPR, participation-to-prevalence ratio; RCTs, randomized clinical trials; SRDs, sex-related differences; W, women.

Trialists should adopt strategies to ensure that the trial is adequately powered to detect statistically significant SRDs; populations should accurately match the sex distribution of CVD; sex stratification should be incorporated as a prespecified subgroup analysis; sex-specific exclusion or inclusion criteria should be justified; sex-specific risk factors and cardiovascular endpoints relevant to women should be included; outcome measurements biased by sex should be eliminated; and results from RCTs should analyse and report drug efficacy and safety separately for women and men.^16,32^ Therefore, the limited information on SRDs of cardiovascular drug efficacy and safety is the result of RCTs underpowered to detect such differences, rather than a true absence of differences.^6^

### Where the evidence for SRDs came from?

For many drugs, data on SRDs did not come from prospective RCTs supporting the approval of cardiovascular drugs, but from retrospective subgroup and posthoc analyses, or from meta-analyses of trials recruiting women with different baseline characteristics.^6,11,18^

Although subgroup analyses in RCTs are used to determine SRDs, they are often poorly conducted and reported. There are several reasons to suspect a lack of validity in subgroup claims including lack of prespecification; inadequate power (false negatives); randomization was not stratified based on sex; lack of adjustment for confounders (age, baseline risk, sex-specific risk factors, comorbidities); multiple comparisons (increased propensity to false positives); analyses were based on published aggregate data, not individual patient-level data; population heterogeneity; and use of *P*-values instead of formal interaction tests. Unless these points are taken into consideration, bias is frequent if not inevitable.^33,34^ Furthermore, most sex-specific analyses were simply descriptive and the mechanisms responsible for the possible SRDs were not analysed.

In a review of HF RCT published in nine high-impact journals from 2008 to 2017, 107 of the 261 RCTs reported subgroup analyses, but the credibility of subgroup claims was generally low across all strengths of claims and one-third of trials claiming subgroup effects failed to report a test of interaction.^35^ In the Treatment of Preserved Cardiac Function Heart Failure with an Aldosterone Antagonist Trial (TOPCAT) trial, enrolling patients with HFpEF (52% women), there were no SRD for the primary outcome [cardiovascular death, aborted cardiac arrest, or heart failure hospitalization (HFH)], but in a non-prespecified subanalysis, a lower risk of all-cause mortality was reported in women, but not in men (*P*_interaction_ = 0.024), despite there were no SRDs in non-CV mortality, CV hospitalization, or non-CV hospitalization.^36^ Surprisingly, this subanalysis supported the recommendation of spironolactone for the treatment of HFpEF. Thus, results from subgroup analyses should be interpreted with caution as they may not serve as guidance for personalized care until they can be replicated in subsequent studies.^33,34^

Results of a meta-analysis are useful when individual trials are not adequately powered to examine events in subgroups, and for many cardiovascular drugs, evidence supporting the lack of SRDs derived from meta-analysis. However, many meta-analyses were not prespecified (to minimize potential bias) or adjusted for multiplicity (to avoid spurious conclusions), and it can be argued that RCTs are not poolable because of important clinical heterogeneity in trial design, inclusion criteria, baseline characteristics, endpoints, treatment regimens, and follow-up. Furthermore, because randomization was not stratified based on sex in individual trials, one cannot exclude the potential impact of confounding on the overall results. It should also be mentioned that by increasing the numbers of women in the meta-analyses the precision of a point estimate might increase, but the accuracy or validity of the treatment effect might not necessarily be improved, and the biases of individual studies might be magnified rather than mitigated when pooled together.^2,33^

A recent meta-analysis of three RCTs recruiting patients with different HF phenotypes (HFrEF: RALES and EMPHASIS-HF trials; HFpEF: TOPCAT-Americas subpopulation), where women represented 27%, 22% and 50% of the population, respectively, concluded that there were no SRDs in the effects of MRAs on cardiovascular death or HFH.^37^ Clearly, this meta-analysis does not provide evidence that there are no SRDs in the effects of MRA in patients with HF.

### There are significant SRDs in drug safety

Women have a higher risk for developing ADRs and cardiovascular drugs appear to account for the most pronounced differences in ADRs between men and women.^4,38-40^ Moreover, ADRs can be more severe (leading to hospitalization) or different in women than in men. SRDs in ADRs produced by cardiovascular drugs are summarized in *Table 2*.

The higher incidence of ADRs in women may result from SRDs in the following:

(1) Drug pharmacokinetics. Women present lower body weight/size, plasma volume, organ blood flow and volume of distribution, higher body fat content, increased activity of cytochromes P450 2B6, 3A4, and 2D6 that are responsible for the metabolism of many cardiovascular drugs and decreased activity of *P*-glycoprotein, and lower renal clearance than men.^4,6^ These differences may explain why the administration of fixed doses, not corrected for body weight or estimated glomerular filtration rate (eGFR), may result in higher drug exposure and ADRs in women than in men.^4,6,38^ Weight-adjusted dosing is recommended for drugs with narrow therapeutic index (e.g. antiarrhythmics, digoxin, antithrombotics) to avoid an increase in the incidence of ADRs. Indeed, the CPG for the management of non-ST-elevation ACS recommended careful attention to weight and eGFR when antithrombotic drugs are prescribed to reduce bleeding risk in women.^41^

An analysis of 300 new drug applications reviewed by the FDA between 1994 and 2000 showed that 31% of studies reported SRDs >20% in pharmacokinetics and >40% in 11 drugs.^42^ Similarly, among 58 sex-nonspecific new molecular entities approved by the FDA between 2000 and 2002, 25 presented SRDs in pharmacokinetics, efficacy, or ADRs.^43^ Another recent study of 86 FDA-approved drugs confirmed that SRDs in pharmacokinetics leading to increased drug exposure predicted sex-specific ADRs in women in 88% of cases, but not in men.^31^ However, despite the existence of SRDs in drug pharmacokinetics, no sex-specific recommendations were included to adjust doses in their prescription labels, and the online FDA database did not provide information on SRDs in pharmacokinetics for drugs approved prior to the year 2000.^31^

(2). Drug pharmacodynamics, related to changes in receptor expression/affinity, signal transduction pathways, genetic factors, or sexual hormonal levels.^5,44^

(3). Women are older, present more comorbidities at the time of CVD diagnosis [chronic kidney disease, musculoskeletal diseases (arthritis, osteoporosis), headaches, anxiety, depressive disorders] and use more medications, prescribed or not (over-the-counter, herbal medicines, supplements, vitamins) than men, leading to complex medication regimens (polypharmacy) which increase the risk of ADRs and drug–drug interactions.^45,46^ These differences, together with those in the response to antithrombotics, may explain the increase in bleeding events in women than in men.^4,47^ Other reasons for the higher use of medicines in women include physiological events (e.g. menstruation, menopause) and that they pay more attention to chronic comorbidities than men.

(4). Both in global postmarketing surveillance programmes and the WHO Global Individual Case Safety Report database (VigiBase) women report more ADRs than men, regardless of age category or drug group.^38^

The higher incidence of ADRs in women negatively affect drug adherence and dose titration, so they are more likely to discontinue life-saving CPGRDs leading to an increase in emergency room visits, hospitalizations, and overall use of healthcare services compared to men.^4,6,40,46^ Despite all this evidence, an analysis of HF drugs recommended in the ESC 2016 HF guidelines (which included 25.2% of women) identified 155 trials. Of these, only 11 (7%) reported ADRs data separately for women and men.^40^ Additionally, in 331 studies cited in the five major CPG on hypertension, only 2 (0.6%) stratified ADRs by sex.^29^ In another review of 224 HF RCTs published between 2000 and 2020 in journals with an impact factor ≥10, no trial reported sex-specific ADRs. These results underline the scarcity of ADRs data stratified by sex. Thus, it is not a surprise that SRDs in drug safety are not incorporated in CPG. Probably, the most effective strategy to minimize the higher incidence of ADRs in women is the development and implementation of sex-specific pharmacological guidelines.

### Which are the right doses of cardiovascular drugs in women?

For decades, preclinical research was based on male-only models and Phase I dose-finding studies were performed in small size (12–24 healthy men) trials powered to detect large differences, but not clinically relevant SRDs. The underutilization of female cells/animals, the use of nonpredictive preclinical models, the limited recruitment of women in RCTs and the inadequate sex-based analysis of clinical data led to the conclusion that SRDs were of limited clinical interest. Indeed, sex differences in pharmacokinetics and pharmacodynamics are currently not considered in CPG, which recommend up-titration to target doses of cardiovascular drugs that are similar in both men and women.^1^ Thus, sex-based personalized adjustments are not integrated in daily clinical practice. However, two questions remain unanswered: are women receiving the optimal doses of cardiovascular drugs? And could they benefit from lower doses than those recommended in the CPG to reduce the incidence of ADRs?

Nowadays, there is evidence of SRDs in pharmacokinetics, efficacy and, particularly, safety of some cardiovascular drugs (i.e. digoxin, loop diuretics, metoprolol, propranolol, sacubitril–valsartan, verapamil) (*Tables 1* and *2*). Indeed, metoprolol doses resulting in similar drug exposure were 100 mg for young men, 50 mg for elderly men and 25 mg for elderly women; thus, a 50% dose reduction for metoprolol was recommended in women.^48^ Furthermore, two trials comparing high doses vs. low doses of losartan or lisinopril in patients with HFrEF found that men might benefit more from higher doses, while lower doses were effective in women (*Table 1*). Of note, a posthoc analysis of the European BIOSTAT-CHF found that in men with HFrEF, the lowest hazards of death or HF hospitalisation occurred at 100% of the CPG-recommended dose of ACEIs, ARBs, or β-blockers, while women showed ∼30% lower risk at only 50% of the recommended doses, with no further decrease in risk at the CPG-recommended doses.^49^ These results, which were confirmed in patients from the ASIAN-HF registry, suggested that women with HFrEF might need lower doses of ACEI/ARB and β-blockers than men, and brings into question what the true optimal medical therapy is for women vs. men. However, all this information is not included in Rx labels or in the CPG.

The current recommendation that women and men should receive the same doses of CPGRDs (‘one-size-fits-all approach’) based on RCTs where women were under-represented, neglects that women may respond differently to cardiovascular drugs than men and ignores the important SRDs on drug safety. Further prospective, sex-specific dose-finding studies are needed to define the optimal doses of CPGRDs in women with CVD and whether sex-specific dose adjustments for body weight or eGFR can reduce the higher incidence of ADRs in women. Administrations and scientific societies should support RCTs to define the optimal doses of many ‘old CV drugs’ that are currently cheap generics in women.

### SRDs in the prescription of guideline-recommended drugs

The prevention and treatment of CVDs is performed using different classes of drugs in men and women, but this practice is not fully explained due to differences in comorbidities. Although recent improvements have been made, women are still less likely to receive CPGRDs for the prevention and treatment of CVDs, and/or drugs are prescribed at lower doses than men with a similar risk, which may contribute to persistent symptoms or suboptimal outcomes. Women are also less likely than men to be referred to a cardiologist, and from a general cardiologist to a tertiary care centre where trial recruitment is taking place.^1,7,8^

In general, women are more likely to be prescribed diuretics, digoxin, and ARB, but less likely ACEIs, MRAs, β-blockers, antiplatelets, statins, or calcium channel blockers than men even after adjusting for different variables^4,6^ (*Table 5*). When antihypertensive, glucose-lowering, or cholesterol-lowering drugs are prescribed, treatment is less aggressive in women, making it more difficult to achieve the recommended target levels for blood pressure, haemoglobin A1C, or low-density lipoprotein-cholesterol. Additionally, women are also less likely than men to be on combination therapy^46,50^ and to receive the monitoring recommended by CPG for diabetes, CAD, or HF.^46^ These SRDs in the prescription of CPGRDs for the prevention and treatment of CVD represent a major health problem and attacks the principle of equitable healthcare.^1,3^ Therefore, effective strategies are needed to address inequities in the prevention and treatment of CVD in women and develop healthcare pathways tailored for women.^1,3,7^ But equitable healthcare cannot be achieved without awareness of healthcare professionals of sex-specific issues (e.g. premature menopause, pregnancy-related disorders) in addition to well-established risk factors for CVDs and better interdisciplinary coordination between cardiologists, PCPs, obstetricians, gynaecologists, and other relevant health professionals who currently operate in silos of care to screen for CVD risk factors and implement risk-reducing strategies across woman’s life course.

**Table 5:** Sex-related differences in the prescription of cardiovascular drugs

Cardiovascular disease	Differences between women and men
Acute coronary syndromes	W were less likely to be prescribed β−blockers, ACEI or ARB, statins, and antithrombotics (heparins, dual antiplatelet therapy, thrombolysis) and less commonly received aspirin, ACEI, and statins at discharge
Antihypertensive drugs	W were prescribed more thiazide and loop diuretics, β−blockers and ARB, but less frequently ACEI, calcium channel blockers or β−blockers than M despite comparable blood pressure levels. The use of 2–3 antihypertensives is less common in W
Atrial fibrillation	W used more often rate-control drugs (β−blockers and digoxin) and M rhythm-control drugs (Class I and III antiarrhythmics) W were significantly less likely than M to use any oral anticoagulant overall and at all levels of CHA_2_DS_2_-VASc score and DOAC were more frequently underdosed despite they have a higher risk of fatal or disabling stroke than M
Coronary artery disease	W were significantly less likely to be treated with antithrombotics, β−blockers and lipid-lowering drugs (statins) than M W less likely to receive CPGRDs for CAD or MI
Cardiogenic shock	W were less likely to receive CPGRDs (aspirin, P2Y_12_ receptor inhibitors, β−blockers, ACEI/ARB, statins) or any parenteral anticoagulation during the hospitalization and were less likely to be discharged on CPGRDs
Diabetes and CVD	W were less likely to receive CPGRDs for patients with diabetes and cardiovascular conditions
Diabetes and HF	W received more β−blockers, ARBs, and diuretics, but less ACEI/ARB than M
DOAC	W were more frequently treated with low-dose dabigatran compared with M
Heart failure	HFrEF: W received more diuretics, ARB and digoxin, but were less frequently treated with ACEI, MRA or β-blockers and, when prescribed, doses were lower than those for M HFmrEF and HFpEF: lower prescription rate of ACEI/ARB in W HFpEF: W received more diuretics and MRA, but were less likely to receive digoxin or β-blockers Acute HF: W were less likely to receive vasoactive therapy and β-blockers and ACEI/ARB or MRA at discharge
Hyperlipidaemia	W with hypercholesterolaemia are less likely to receive high-intensity statin therapy and to reach LDL-C targets than M
Hypertension and CAD	Swedish Primary Care Cardiovascular Database: W received more ARB and MRA; ACEI were more frequently prescribed in M
Hypertension and CKD	The overall use of ACEI, β-blockers and calcium channel blockers was lower, while the use of ARB was higher in W than in M
Warfarin	Meta-analysis of 28 observational studies: W were significantly less likely to receive warfarin than M
Patients with established CVD	Meta-analysis of 43 studies (2 264 600 participants; 28% W): W were less likely to be prescribed aspirin, statins or ACEI, but were more likely to receive diuretics. No SRDs in the prescription of β-blockers and calcium channel blockers

References are presented in Supplementary material online, *Table S6*.

ACEIs, angiotensin-converting enzyme inhibitors; ARBs, angiotensin receptor blockers; CAD, coronary artery disease; CKD, chronic kidney disease; CPGRDs, clinical practice guidelines-recommended drugs; CVD, cardiovascular disease; DOAC, direct oral anticoagulants; HFmrEF/HFpEF/HFrEF, heart failure with mildly reduced/preserved/reduced ejection fraction; LDL-C, low-density lipoprotein-cholesterol; M, men; MI, myocardial infarction; MRA, mineralocorticoid receptor antagonists; SRDs, sex-related differences; W, women.

Although the reasons for the SRDs in the prescription of CPGRDs are beyond the scope of this article, they may be related, among others, to insufficient awareness of cardiovascular risk among women, PCPs, and cardiologists; differences in physicians’ interpretation of women's symptoms and time of treatment with respect to the progression of CVD; limited training to assess CVD risk and use of guideline assessment in women; limited information on the efficacy of some cardiovascular drugs in women; higher incidence of ADRs in women; and physicians’ sex, as male physicians used significantly less medications and lower doses in women.^9,46^

A recent analysis of 24 CPG produced by the ESC between 2018 and 2023 found that only 11 of 23 CPG included a section dedicated to SRDs; SRDs were cited in the ‘key points’ section in only two CPG; the lack of sufficient information about SRDs was reported in the ‘gaps in knowledge’ section in six CPG; articles with titles related to sex differences made up only 1.5% of total citations; and sometimes, information on SRDs appeared in the appendix but not in the main text.^51^ Thus, it was concluded that although SRDs play a significant role in most clinical scenarios, ESC guidelines still do not sufficiently account for this, and issues relating to sex differences in CV drug efficacy and safety are still poorly represented in CPG.

Despite all these criticisms, it is important to recognize that in the last decade, the leading international cardiology societies (ESC, AHA, ACC) have published specific recommendations and guidelines that emphasize the importance of sex-specific reporting of RCTs findings.^2,3,7,28,52^ Following the publication of the ESC Gender Policy document in 2022,^53^ the most recent ESC guidelines emphasize the importance of increasing women’s representation in future RCTs; patient recruitment according to real-world populations to ensure the generalizability of RCT findings; sex-stratified analyses to better inform the optimal management of CVD in women; and ensuring that women included in ongoing RCTs receive CPGRDs like men. CPG also include short sections or subsections dedicated to SRDs, and sentences on sex/gender medicine are more frequent in ‘key points’ and ‘gaps of knowledge’.^51^ However, it is necessary to take a few years to understand and evaluate the impact of the ESC Gender Policy on the current situation.

### Limitations of this review

Limitations of this review are summarized in Supplementary material online, *Table S7*.

## Conclusions

The limited information on SRDs about dosage, efficacy, and safety of cardiovascular drugs is due to the poor awareness among patients, healthcare providers, and policymakers regarding CVDs that represent the leading cause of death among women, under-representation of women, and lack of sex-specific analysis and reporting of cardiovascular drug efficacy and safety in many RCTs. Therefore, at the present time, it seems unlikely to conclude that there are no SRDs in the efficacy and safety of GRHFDs. Thus, and despite recent advances in many areas of cardiovascular research, CVDs in women remain understudied, under-recognized, underdiagnosed, and undertreated globally.^1,8^ Thus, sex-specific strategies are urgently needed to provide optimal healthcare for women. The main gaps in knowledge and future steps to improve our understanding of SRDs and optimize the prevention and treatment of CVD for women are summarized in *Table 6* and the *Graphical abstract*. The limited data on the clinical relevance of SRDs should be the stimulus for basic and clinical research to generate the scientific evidence needed to fill our current knowledge gaps, so that prescribers can make informed decisions based on sound scientific data. A better understanding of the mechanisms underlying SRDs in pharmacokinetics, efficacy, and safety of CPGRDs is the first step to develop sex-specific therapeutic strategies. The final aim is to ensure that both men and women receive the most effective and safe personalized pharmacotherapy for the prevention and treatment of CVDs.

**Table 6:** Gaps in knowledge and steps to improve our understanding of SRDs in dosing, efficacy and safety of CV drugs

Present gaps	Next steps
Poor awareness of CVD	Develop educational programmes among patients, healthcare providers, and policymakers to increase awareness that CVD represent the leading cause of death among W
The mechanisms underlying the SRDs remains uncertain	Evaluate the potential of SRDs in CV drug efficacy and safety at each step of drug development A deeper understanding of the mechanisms behind these SRDs is needed to provide the best clinical healthcare and minimize the higher incidence of ADRs in women Increase funding supporting basic and clinical research of SRDs
Under-representation of W in CV RCTs	Develop effective strategies (including digital tools) to improve recruitment and retention of W in CV RCTs Identify and remove barriers and unnecessary exclusion criteria limiting women enrolment
Study design does not allow to report SDRs	Prospective RCTs should be specifically designed to analyse and report potential differences in dosing, efficacy, and safety of CV drugs for W and M separately, and to establish appropriate management strategies for women. Outcomes particularly important for W should be considered in the design of RCTs Prioritize research funding in CVD specific to W or predominantly affecting W Regulatory agencies, scientific journals, and funding agencies should require the analysis and report of sex-stratified results
Underuse of CPGRDs in women	Increasing the prescription and adherence to CPGRDs for the prevention and treatment of CVD represents the most efficient way to improve clinical outcomes in W
Define the optimal doses	Early drug development programmes should define sex-appropriate dosing Support prospective RCTs to define the optimal doses of CPGRDs in W and M
Poor translation of SRDs into clinical practice	Evidence-based SRDs should be considered when selecting the drug and dosage SRDs in CV drug efficacy and safety should be incorporated in CPG, medical education, drug labels, and websites
Lack of sex-specific CPG	Developing and implementing sex-specific CPG for CV prevention and treatment is an effective strategy to improve clinical outcomes and minimize the higher incidence of ADRs in W
Poor communication between healthcare providers	Prevention and management of CVD in W require a multidisciplinary care approach involving cardiologists and other health professionals (obstetricians and gynaecologists, among others) throughout a woman's life
Improve education	Education on SRDs in cardiovascular drug efficacy and safety should be embedded in the medical curriculum and reinforced in continuing medical education programmes

References are presented in Supplementary material online, *Table S8*.

ADRs, adverse drug reactions; CV, cardiovascular; CVD, cardiovascular disease; CPG, clinical practice guidelines; CPGRDs, CPG-recommended drugs; RCTs, randomized clinical trials; SRDs, sex-related differences; W, women.

## Supplementary Material

pvaf057_Supplementary_Data

## Data Availability

There are no new data associated with this article.

## References

[1] Vogel B, Acevedo M, Appelman Y, Bairey Merz CN, Chieffo A, Figtree GA, Guerrero M, Kunadian V, Lam CSP, Maas AHEM, Mihailidou AS, Olszanecka A, Poole JE, Saldarriaga C, Saw J, Zühlke L, Mehran R. The Lancet women and cardiovascular disease commission: reducing the global burden by 2030. Lancet 2021;397:2385–2438. 34010613 10.1016/S0140-6736(21)00684-X

[2] Mosca L, Benjamin EJ, Berra K, Bezanson JL, Dolor RJ, Lloyd-Jones DM, Newby LK, Piña IL, Roger VL, Shaw LJ, Zhao D, Beckie TM, Bushnell C, D’Armiento J, Kris-Etherton PM, Fang J, Ganiats TG, Gomes AS, Gracia CR, Haan CK, Jackson EA, Judelson DR, Kelepouris E, Lavie CJ, Moore A, Nussmeier NA, Ofili E, Oparil S, Ouyang P, Pinn VW, Sherif K, Smith SC, Sopko G, Chandra-Strobos N, Urbina EM, Vaccarino V, Wenger NK. Effectiveness-based guidelines for the prevention of cardiovascular disease in women—2011 update: a guideline from the American Heart Association. J Am Coll Cardiol 2011;57:1404–1423. 21388771 10.1016/j.jacc.2011.02.005 PMC3124072

[3] Shaw LJ, Pepine CJ, Xie J, Mehta PK, Morris AA, Dickert NW, Ferdinand KC, Gulati M, Reynolds H, Hayes SN, Itchhaporia D, Mieres JH, Ofili E, Wenger NK, Bairey Merz CN. Quality and equitable health care gaps for women: attributions to sex differences in cardiovascular medicine. J Am Coll Cardiol 2017;70:373–388. 28705320 10.1016/j.jacc.2017.05.051

[4] Tamargo J, Rosano G, Walther T, Duarte J, Niessner A, Kaski JC, Ceconi C, Drexel H, Kjeldsen K, Savarese G, Torp-Pedersen C, Atar D, Lewis BS, Agewall S. Gender differences in the effects of cardiovascular drugs. Eur Heart J Cardiovasc Pharmacother 2017;3:163–182. 28329228 10.1093/ehjcvp/pvw042

[5] EUGenMed Cardiovascular Clinical Study Group; Regitz-Zagrosek V, Oertelt-Prigione S, Prescott E, Franconi F, Gerdts E, Foryst-Ludwig A, Maas AH, Kautzky-Willer A, Knappe-Wegner D, Kintscher U, Ladwig KH, Schenck-Gustafsson K, Stangl V. Gender in cardiovascular diseases: impact on clinical manifestations, management, and outcomes. Eur Heart J 2016;37:24–34. 26530104 10.1093/eurheartj/ehv598

[6] Delpón E, Tamargo J. Cardiovascular therapy in women with hypertension or heart failure. In: Gerdts E Maas AH, eds. Manual of Cardiovascular Disease in Women. Cham, Switzerland: Springer Nature Switzerland AG; 2024. p. 419–443.

[7] Wenger NK, Lloyd-Jones DM, Elkind MSV, Fonarow GC, Warner JJ, Alger HM, Cheng S, Kinzy C, Hall JL, Roger VL. Call to action for cardiovascular disease in women: epidemiology, awareness, access, and delivery of equitable health care: a presidential advisory from the American Heart Association. Circulation 2022;145:e1059–e1071. 35531777 10.1161/CIR.0000000000001071 PMC10162504

[8] Burgess SN . Understudied, under-recognized, underdiagnosed, and undertreated: sex-based disparities in cardiovascular medicine. Circ Cardiovasc Interv 2022;15:e011714. 35067073 10.1161/CIRCINTERVENTIONS.121.011714

[9] Bairey Merz CN, Andersen H, Sprague E, Burns A, Keida M, Walsh MN, Greenberger P, Campbell S, Pollin I, McCullough C, Brown N, Jenkins M, Redberg R, Johnson P, Robinson B. Knowledge, attitudes, and beliefs regarding cardiovascular disease in women: the women’s heart alliance. J Am Coll Cardiol 2017;70:123–132. 28648386 10.1016/j.jacc.2017.05.024

[10] McDonnell LA, Turek M, Coutinho T, Nerenberg K, Margerie Md, Perron S, Reid RD, Pipe AL. Women’s heart health: knowledge, beliefs, and practices of Canadian physicians. J Womens Health (Larchmt) 2018;27:72–82. 28605313 10.1089/jwh.2016.6240

[11] Scott PE, Unger EF, Jenkins MR, Southworth MR, McDowell T-Y, Geller RJ, Elahi M, Temple RJ, Woodcock J. Participation of women in clinical trials supporting FDA approval of cardiovascular drugs. J Am Coll Cardiol 2018;71:1960–1969. 29724348 10.1016/j.jacc.2018.02.070

[12] Melloni C, Berger JS, Wang TY, Gunes F, Stebbins A, Pieper KS, Dolor RJ, Douglas PS, Mark DB, Newby LK. Representation of women in randomized clinical trials of cardiovascular disease prevention. Circ Cardiovasc Qual Outcomes 2010;3:135–142. 20160159 10.1161/CIRCOUTCOMES.110.868307

[13] Sardar MR, Badri M, Prince CT, Seltzer J, Kowey PR. Underrepresentation of women, elderly patients, and racial minorities in the randomized trials used for cardiovascular guidelines. JAMA Intern Med 2014;174:1868–1870. 25264856 10.1001/jamainternmed.2014.4758

[14] Gong IY, Tan NS, Ali SH, Lebovic G, Mamdani M, Goodman SG, Ko DT, Laupacis A, Yan AT. Temporal trends of women enrollment in major cardiovascular randomized clinical trials. Can J Cardiol 2019;35:653–660. 31030866 10.1016/j.cjca.2019.01.010

[15] Jin X, Chandramouli C, Allocco B, Gong E, Lam CSP, Yan LL. Women’s participation in cardiovascular clinical trials from 2010 to 2017. Circulation 2020;141:540–548. 32065763 10.1161/CIRCULATIONAHA.119.043594

[16] Khan MS, Shahid I, Siddiqi TJ, Khan SU, Warraich HJ, Greene SJ, Butler J, Michos ED. Ten-year trends in enrollment of women and minorities in pivotal trials supporting recent US food and drug administration approval of novel cardiometabolic drugs. J Am Heart Assoc 2020;9:e015594. 32427023 10.1161/JAHA.119.015594 PMC7428976

[17] Tahhan AS, Vaduganathan M, Greene SJ, Fonarow GC, Fiuzat M, Jessup M, Lindenfeld JA, O’Connor CM, Butler J. Enrollment of older patients, women, and racial and ethnic minorities in contemporary heart failure clinical trials: a systematic review. JAMA Cardiol 2018;3:1011–1019. 30140928 10.1001/jamacardio.2018.2559

[18] Levinsson A, Dubé M-P, Tardif J-C, de Denus S. Sex, drugs, and heart failure: a sex-sensitive review of the evidence base behind current heart failure clinical guidelines. ESC Heart Fail 2018;5:745–754. 29916560 10.1002/ehf2.12307 PMC6165928

[19] Carcel C, Harris K, Peters SAE, Sandset EC, Balicki G, Bushnell CD, Howard VJ, Reeves MJ, Anderson CS, Kelly PJ, Woodward M. Representation of women in stroke clinical trials: a review of 281 trials involving more than 500,000 participants. Neurology 2021;97:e1768–e1774. 34645708 10.1212/WNL.0000000000012767

[20] Geller SE, Koch A, Pellettieri B, Carnes M. Inclusion, analysis, and reporting of sex and race/ethnicity in clinical trials: have we made progress? J Womens Health (Larchmt) 2011;20:315–320. 21351877 10.1089/jwh.2010.2469 PMC3058895

[21] Welch V, Doull M, Yoganathan M, Jull J, Boscoe M, Coen SE, Marshall Z, Pardo JP, Pederson A, Petkovic J, Puil L, Quinlan L, Shea B, Rader T, Runnels V, Tudiver S. Reporting of sex and gender in randomized controlled trials in Canada: a cross-sectional methods study. Res Integr Peer Rev 2017;2:15. 29451565 10.1186/s41073-017-0039-6 PMC5803639

[22] Au M, Whitelaw S, Khan MS, Mamas MA, Mbuagbaw L, Mulvagh SL, Voors AA, Van Spall HGC. A systematic review of sex-specific reporting in heart failure clinical trials: trial flow and results. JACC Adv 2022;1:100079. 38939721 10.1016/j.jacadv.2022.100079 PMC11198397

[23] Granton D, Rodrigues M, Raparelli V, Honarmand K, Agarwal A, Friedrich JO, Perna B, Spaggiari R, Fortunato V, Risdonne G, Kho M, VanderKaay S, Chaudhuri D, Gomez-Builes C, D'Aragon F, Wiseman D, Lau VI, Lin C, Reid J, Trivedi V, Prakash V, Belley-Cote E, Al Mandhari M, Thabane L, Pilote L, Burns KEA. Sex and gender-based analysis and diversity metric reporting in acute care trials published in high-impact journals: a systematic review. BMJ Open 2024;14:e081118. 10.1136/bmjopen-2023-081118 PMC11103199 38719297

[24] Doull M, Runnels VE, Tudiver S, Boscoe M. Appraising the evidence: applying sex- and gender-based analysis (SGBA) to Cochrane systematic reviews on cardiovascular diseases. J Womens Health (Larchmt) 2010;19:997–1003. 20384450 10.1089/jwh.2009.1626

[25] Petkovic J, Trawin J, Dewidar O, Yoganathan M, Tugwell P, Welch V. Sex/gender reporting and analysis in Campbell and Cochrane systematic reviews: a cross-sectional methods study. Syst Rev 2018;7:113. 30068380 10.1186/s13643-018-0778-6 PMC6090880

[26] DeFilippis EM, Van Spall HGC. Is it time for sex-specific guidelines for cardiovascular disease? J Am Coll Cardiol 2021;78:189–192. 34238440 10.1016/j.jacc.2021.05.012

[27] Bastian-Pétrel K, Rohmann JL, Oertelt-Prigione S, Piccininni M, Gayraud K, Kelly-Irving M, Bajos N. Sex and gender bias in chronic coronary syndromes research: analysis of studies used to inform the 2019 European Society of Cardiology guidelines. Lancet Reg Health Eur 2024;45:101041. 39279866 10.1016/j.lanepe.2024.101041 PMC11402417

[28] Schreuder MM, Boersma E, Kavousi M, Visser LE, Roos-Hesselink JW, Versmissen J, Roeters van Lennep JE. Reporting of sex-specific outcomes in trials of interventions for cardiovascular disease: has there been progress? Maturitas 2021;144:1–3. 33358200 10.1016/j.maturitas.2020.09.007

[29] Gulamhusein N, Turino Miranda K, Dumanski SM, González Bedat MC, Ulasi I, Conjeevaram A, Ahmed SB. Sex- and gender-based reporting in antihypertensive medication literature informing hypertension guidelines. J Am Heart Assoc 2024;13:e030613. 38420762 10.1161/JAHA.123.030613 PMC10944031

[30] Mohseni-Alsalhi Z, Vesseur MAM, Wilmes N, Laven SAJS, Meijs DAM, van Luik EM, Vaes EWP, Dikovec CJR, Wiesenberg J, Almutairi MF, Janssen EBNJ, de Haas S, Spaanderman MEA, Ghossein-Doha C. The representation of females in studies on antihypertensive medication over the years: a scoping review. Biomedicines 2023;11:1435. 37239106 10.3390/biomedicines11051435 PMC10216827

[31] Zucker I, Prendergast BJ. Sex differences in pharmacokinetics predict adverse drug reactions in women. Biol Sex Differ 2020;11:32. 32503637 10.1186/s13293-020-00308-5 PMC7275616

[32] Zannad F, Berwanger O, Corda S, Cowie MR, Gamra H, Gibson CM, Goncalves A, Hucko T, Khunti K, Kostrubiec M, Kraus BJ, Linde C, Lüscher TF, Mafham M, Mindham R, Ortega RF, Prescott E, Thabane L, Yancy C, Ziegler A, Van Spall HGC. How to make cardiology clinical trials more inclusive. Nat Med 2024;30:2745–2755. 39402268 10.1038/s41591-024-03273-3

[33] Sun X, Briel M, Busse JW, You JJ, Akl EA, Mejza F, Bala MM, Bassler D, Mertz D, Diaz-Granados N, Vandvik PO, Malaga G, Srinathan SK, Dahm P, Johnston BC, Alonso-Coello P, Hassouneh B, Walter SD, Heels-Ansdell D, Bhatnagar N, Altman DG, Guyatt GH. Credibility of claims of subgroup effects in randomised controlled trials: systematic review. BMJ 2012;344:e1553. 22422832 10.1136/bmj.e1553

[34] Burke JF, Sussman JB, Kent DM, Hayward RA. Three simple rules to ensure reasonably credible subgroup analyses. BMJ 2015;351:h5651. 26537915 10.1136/bmj.h5651 PMC4632208

[35] Khan MS, Khan MAA, Irfan S, Siddiqi TJ, Greene SJ, Anker SD, Sreenivasan J, Friede T, Tahhan AS, Vaduganathan M, Fonarow GC, Butler J. Reporting and interpretation of subgroup analyses in heart failure randomized controlled trials. ESC Heart Fail 2021;8:26–36. 33254286 10.1002/ehf2.13122 PMC7835611

[36] Merrill M, Sweitzer NK, Lindenfeld J, Kao DP. Sex differences in outcomes and responses to spironolactone in heart failure with preserved ejection fraction: a secondary analysis of TOPCAT trial. JACC Heart Fail 2019;7:228–238. 30819379 10.1016/j.jchf.2019.01.003 PMC6817333

[37] Rossello X, Ferreira JP, Pocock SJ, McMurray JJV, Solomon SD, Lam CSP, Girerd N, Pitt B, Rossignol P, Zannad F. Sex differences in mineralocorticoid receptor antagonist trials: a pooled analysis of three large clinical trials. Eur J Heart Fail 2020;22:834–844. 32077220 10.1002/ejhf.1740

[38] Watson S, Caster O, Rochon PA, Ruijter H. Reported adverse drug reactions in women and men: aggregated evidence from globally collected individual case reports during half a century. EClinicalMedicine 2019;17:100188. 31891132 10.1016/j.eclinm.2019.10.001 PMC6933269

[39] Rodenburg EM, Stricker BHC, Visser LE. Sex-related differences in hospital admissions attributed to adverse drug reactions in The Netherlands. Br J Clin Pharmacol 2011;71:95–104. 21143505 10.1111/j.1365-2125.2010.03811.x PMC3018030

[40] Bots SH, Groepenhoff F, Eikendal ALM, Tannenbaum C, Rochon PA, Regitz-Zagrosek V, Miller VM, Day D, Asselbergs FW, den Ruijter HM. Adverse drug reactions to guideline-recommended heart failure drugs in women: a systematic review of the literature. JACC Heart Fail 2019;7:258–266. 30819382 10.1016/j.jchf.2019.01.009

[41] Amsterdam EA, Wenger NK, Brindis RG, Casey DE, Ganiats TG, Holmes DR, Jaffe AS, Jneid H, Kelly RF, Kontos MC, Levine GN, Liebson PR, Mukherjee D, Peterson ED, Sabatine MS, Smalling RW, Zieman SJ. 2014 AHA/ACC guideline for the management of patients with non-ST-elevation acute coronary syndromes: a report of the American College of Cardiology/American Heart Association Task Force on Practice Guidelines. J Am Coll Cardiol 2014;64:e139–e228. 25260718 10.1016/j.jacc.2014.09.017

[42] Anderson GD . Sex and racial differences in pharmacological response: where is the evidence? Pharmacogenetics, pharmacokinetics, and pharmacodynamics. J Womens Health (Larchmt) 2005;14:19–29. 15692274 10.1089/jwh.2005.14.19

[43] Yang Y, Carlin AS, Faustino PJ, Motta MIP, Hamad ML, He R, Watanuki Y, Pinnow EE, Khan MA. Participation of women in clinical trials for new drugs approved by the food and drug administration in 2000–2002. J Womens Health (Larchmt) 2009;18:303–310. 19243271 10.1089/jwh.2008.0971

[44] Tamargo J, Agarwall NR, Tamargo M. Pharmacotherapy considerations in men and women: cardiovascular mediciations. In: Aggarwal NR Wood MJ, eds. Sex Differences in Cardiac Diseases. Pathophysiology, Presentation, Diagnosis and Treatment. Cambridge, USA: Elsevier; 2021. p. 591–641.

[45] Temkin SM, Barr E, Moore H, Caviston JP, Regensteiner JG, Clayton JA. Chronic conditions in women: the development of a national institutes of health framework. BMC Womens Health 2023;23:162. 37024841 10.1186/s12905-023-02319-x PMC10077654

[46] Manteuffel M, Williams S, Chen W, Verbrugge RR, Pittman DG, Steinkellner A. Influence of patient sex and gender on medication use, adherence, and prescribing alignment with guidelines. J Womens Health (Larchmt) 2014;23:112–119. 24206025 10.1089/jwh.2012.3972

[47] Capodanno D, Patel A, Dharmashankar K, Ferreiro JL, Ueno M, Kodali M, Tomasello SD, Capranzano P, Seecheran N, Darlington A, Tello-Montoliu A, Desai B, Bass TA, Angiolillo DJ. Pharmacodynamic effects of different aspirin dosing regimens in type 2 diabetes mellitus patients with coronary artery disease. Circ Cardiovasc Interv 2011;4:180–187. 21386092 10.1161/CIRCINTERVENTIONS.110.960187

[48] Eugene AR . Gender based dosing of metoprolol in the elderly using population pharmacokinetic modeling and simulations. Int J Clin Pharmacol Toxicol 2016;5:209–215. 27468378 PMC4959610

[49] Santema BT, Ouwerkerk W, Tromp J, Sama IE, Ravera A, Regitz-Zagrosek V, Hillege H, Samani NJ, Zannad F, Dickstein K, Lang CC, Cleland JG, Ter Maaten JM, Metra M, Anker SD, van der Harst P, Ng LL, van der Meer P, van Veldhuisen DJ, Meyer S, Lam CSP, Voors AA, Richards AM, Lam CSP, Anand I, Hung CL, Ling LH, Liew HB, Narasimhan C, Ngarmukos T, Park SW, Reyes E, Siswanto BB, Shimizu W, Zhang S. Identifying optimal doses of heart failure medications in men compared with women: a prospective, observational, cohort study. Lancet 2019;394:1254–1263. 31447116 10.1016/S0140-6736(19)31792-1

[50] Mosca L, Hammond G, Mochari-Greenberger H, Towfighi A, Albert MA; American Heart Association Cardiovascular Disease and Stroke in Women and Special Populations Committee of the Council on Clinical Cardiology, Council on Epidemiology and Prevention, Council on Cardiovascular Nursing, Council on High Bloo. Fifteen-year trends in awareness of heart disease in women: results of a 2012 American Heart Association national survey. Circulation 2013;127:1254–1263, e1–29. 23429926 10.1161/CIR.0b013e318287cf2f PMC3684065

[51] Piani F, Baffoni L, Strocchi E, Borghi C. Gender-specific medicine in the European Society of Cardiology Guidelines from 2018 to 2023: where are we going? J Clin Med 2024;13:4026. 39064064 10.3390/jcm13144026 PMC11278282

[52] Mehta LS, Beckie TM, DeVon HA, Grines CL, Krumholz HM, Johnson MN, Lindley KJ, Vaccarino V, Wang TY, Watson KE, Wenger NK; American Heart Association Cardiovascular Disease in Women and Special Populations Committee of the Council on Clinical Cardiology, Council on Epidemiology and Prevention, Council on Cardiovascular and Stroke Nursing, and Council on Quality of Care and Outcomes Research. Acute myocardial infarction in women: a scientific statement from the American Heart Association. Circulation 2016;133:916–947. 26811316 10.1161/CIR.0000000000000351

[53] ESC Gender Policy . https://www.escardio.org/static-file/Escardio/About%20the%20ESC/Documents/European%20Society%20of%20Cardiology%20Gender%20Policy.pdf (Accessed 6 May 2025).

