# Supplementary material for: Sex-related differences in cardiovascular pharmacotherapy: fiction or fact? Why can’t we see the evidence?
Source: Eur Heart J Cardiovasc Pharmacother. 2025 Aug 4;11(7):638–52. doi: 10.1093/ehjcvp/pvaf057 (PMC12582654; doi:10.1093/ehjcvp/pvaf057)
Supplement: pvaf057_Supplementary_Data [file pvaf057_supplementary_data.doc]

**Supplemental Table 1. Search terms, data sources and strategy terms**

| **Variables** | **Inclusion** |
| --- | --- |
| Medical subject heading/Key words | "sex”, “gender", “adverse reactions”, “cardiovascular drugs”, “cardiovascular disease”, “cardiovascular pharmacotherapy”, “clinical practice guidelines”, “clinical trials”, “differences”, “dose differences”, “meta-analysis”, “pharmacodynamics”, “pharmacokinetics” and “treatment disparities” |
| Publications included | Randomized clinical trials, post-hoc analysis, systematic reviews, meta-analyses, clinical guidelines |
| Publications excluded | Abstracts, conference proceedings, case reports, non-peer-reviewed |
| Databases | PubMed, Embase, Cochrane Library of Clinical Trials, Web of Science |
| Language | English language articles |
| Years of publication | Between January 1, 2000, and December 1, 2020 |
| Country of origin | Western countries |

**Supplemental Table 2. Sex-related differences in the pharmacokinetics and pharmacodynamics of guideline-recommended cardiovascular drugs**

| **Drug class** | **Differences in pharmacokinetics/pharmacodynamics between women and men** |
| --- | --- |
| Angiotensin- converting enzyme inhibitors | - W were underrepresented in pivotal HF trials and sex interactions were not provided^1–3^ - HFrEF: improvement in survival appeared to be greater in M (37%) than in W (22%)^4,5^ - ATLAS trial: M might benefit more from higher doses of lisinopril, whereas lower doses were effective in W^6^ - HFrEF: W may have the lowest risk of death or HFH at half the guideline-recommended doses compared with M^7^ - W with asymptomatic LV systolic dysfunction did not achieve a mortality benefit when treated with ACEI^5^ |
| Antiarrhythmics | - Weight adjustment for loading doses of digoxin and class I and III antiarrhythmic drugs are recommended^8,9^ - Procainamide: higher plasma levels (30%) in W due to a lower BMI and Vd^10^ - Lidocaine: significantly higher free plasma levels in W receiving OC (oestrogens reduce α1-acid glycoprotein levels)^11^ |
| Anticoagulants | - UFH is cleared more rapidly in M than in W^12,13^ and W had higher heparin levels and aPTT values than M^14^ - Post-MI: W treated with UFH achieve higher aPTT levels than M, and aPTT >70 sec increased the risk of death, stroke, bleeding, and reinfarction^15^ - ACS (ESSENCE and TIMI 11B trials): significantly greater benefit of enoxaparin over UFH in W, but not in M^16^ - Higher anti-Xa activity of dalteparin in W than in M (P<0.001)^17^ - Non-cardiac arterial procedures: W reached significantly higher levels of anticoagulation than M^18^ - Bivaluridin (REPLACE-2, ACUITY, HORIZONS-AMI trials): more pronounced benefit in reducing 1-year mortality in W than in M^19,20^ |
| Antiplatelets (except aspirin) | - Exposure to ticagrelor and AR-C124910XX is ~ 40–60 % higher W in than in M, but no dose adjustment is recommended^21^ - Patients undergoing PCI (29 RCT; n=99,591; 25.2% W): W present a higher risk of MACCE and bleeding events when newer P2Y12 inhibitors are used^22^ - MI (5 RCT; n=79,613; 30% W): clopidogrel-reduced MACE was driven by a reduction in MI in W and by a reduction in MI, stroke and all-cause mortality in M^23^ - GP IIb/IIIa inhibitors in NST-ACS (6 RCT; n=31,492; 35% W): treatment benefit in M but not in W (P<0.0001); no SRD when patients were stratified according to troponin levels^24^ - The net clinical benefit of antiplatelets tends to be generally smaller in W than in M^25^ |
| Angiotensin receptor blockers | - W were underrepresented in hypertension and HF trials and sex-specific analysis were not provided^1,3,26^ - W with hypertension or HF are more likely to receive ARB^2,3,27^ - HFrEF: W may have the lowest risk of death or HFH at half the guideline-recommended doses compared with M^7^ - HEAAL trial: M appeared to benefit more from high-doses of losartan; W responded similarly to low or high doses^28^ |
| Aspirin | - Greater oral bioavailability, larger Vd, slower Cl due reduced glycine conjugation and longer half-life in W than in M^29,30^ - Higher prevalence of aspirin resistance in W than in M because of increased baseline platelet reactivity^31–33^ - Aspirin may be less effective at inhibiting platelet aggregation in W than in M with a history of ischemic stroke or TIA^34^ - MI prevention (23 trials; n=113,494; 50% W): trials recruiting predominantly M showed the largest reduction (38%) in non-fatal MI, while trials recruiting mostly W failed to demonstrate any benefits^35^ |
| Beta-blockers | - Higher exposure of CYP2D6-dependent β-blockers (carvedilol, metoprolol, nebivolol, propranolol) in W due to higher oral bioavailability, lower Vd and slower metabolism^36^. Post-MI: propranolol exposure was significantly higher in W than in M^37^ - Larger decrease in heart rate and blood pressure, in W than in M on similar doses^38,39^ - Similar metoprolol exposure after 25 mg in elderly W, 50 mg in elderly M, and 100 mg in adult M; reduce the dose in W (50%)^40^ - RCT were underpowered to assess SRD in patients with HFpEF^1^ or HFrEF^41,42^ - HFrEF: W may have the lowest risk of death or HFH at half the CPG-recommended doses compared with M^7^ - CIBIS II trial: bisoprolol significantly decreased all-cause, CV, and pump failure deaths in W than in M with HFrEF^43^ |
| Calcium channel blockers | - W present faster Cl and lower exposure of nifedipine and verapamil, due to the lower BMI, higher activity of CYP3A4 and lower activity of P-gp^38,44–47^ - Amlodipine and verapamil clearance decreases in W with aging; amlodipine produces more pronounced BP reduction in elderly W^46,48^. However, major RCT did not found SRD in hypertensive patients^2^ - HOT trial: fewer MI in the lowest diastolic BP target group (<80-85 mmHg) in W, but not in M^49^ |
| Digoxin | - Higher SDC in W due to reduced Vd and lower renal Cl^50,51^ - HFrEF: pivotal trials (DIG, PROVED, RADIANCE) did not perform sex-specific analysis^3^ |
| Diuretics (thiazide and loop) | - No study has evaluated possible SRD in patients with hypertension of HF^52–56^ - Lower Cl and higher exposure (40-50%) of torasemide, but no dose adjustment is recommended^57^ - Thiazides improve stroke and MACE in W and total and specific mortality, all coronary events, all strokes, and MACE in M^58^ |
| Direct oral anticoagulants | - Dabigatran exposure is ~30% higher in W but no dose adjustment is indicated^59^ - Major RCT were not designed to conduct sex-specific analyses, but doses were adjusted by body weight and renal function, which implies some correction in W^38,60^ - ARISTOTLE trial: among patients with AF and previous history of stroke/TIA, W have a lower risk of recurrent stroke and all-cause and CV death than M (both P<0.0001)^61^ - Nationwide Taiwanese cohort: greater risk reduction of ischemic stroke W as compared with M (P=0.04)^62^ - Meta-analysis of 4 RCT (37% W): M were more protected from stroke/SEE and W from major bleeding events^63^ - AF (5 RCT, 66,389 patients, 37.8% W): W treated with DOAC were at higher risk of stroke and systemic embolism than M^64^ - Compared with warfarin, DOAC use was associated with a lower risk of ICH and all-cause mortality in W but not in M^65^ - Acute VTE: in RCT all-cause mortality was not reported by sex^66,67^ |
| ET-1 receptor antagonists | - 6 RCT (n=1,130; 26% W): W experienced significantly greater benefits in terms of change in 6MWD than M^68^ |
| GLP-1 receptor agonists | - T2D (7 CVOTs; n=56,004): GLP-1RA significantly reduce MACE in M, but not in W, as compared with placebo^69^ - T2D: greater effectiveness of GLP-1RA in W than M^70^ |
| Isosorbide dinitrate | - Higher drug exposure in W probably due to their lower body weight^71^ |
| Isosorbide dinitrate/  hydralazine | - Isosorbide dinitrate/hydralazine seemed to have a more pronounced mortality benefit in women, but there was no significant treatment interaction by sex^72^ |
| MRA* | - Serum aldosterone levels are directly related with LV concentric remodeling in W but not in M^73^ - AA W (but not AA M, or white W) on spironolactone had lower BP and less uncontrolled hypertension^74^ - HFpEF: in a non-pre-specified subanalysis of TOPCAT-Americas, spironolactone reduced all-cause mortality in W, but not in M^75^ - AMI and LV dysfunction: a trend towards greater benefit for 30-days all-cause mortality in W treated with eplerenone^76^ |
| Sacubitril-valsartan* | - HFpEF (PARAGON trial, 51.7% W): sacubitril/valsartan significantly reduced CV and HFH in W but not in M due to a greater reduction in HFH^77,78^ |
| SGLT2 inhibitors | - EMPEROR-Reduced trial: larger benefit with empagliflozin vs placebo for the composite of CV death or HFH in W (HR 0.59) than in M (HR 0.80)^79^ - HF (5 RCT; n= 21,948; 35.7% W): SGLT2 inhibitors reduced the composite of CV death and HFH, but the benefit was less pronounced in W^80^ - T2D (3 CVOT; n= 34,322): SGLT2I reduced MACE in M, but not in W^69^ - Meta-regression analysis (26 RCT; n=22,256; 58% W): significant reduction in all-cause mortality when the percentage of females was ≤50% but not when it was >50%^81^ |
| Statins | Statins have not been adequately tested in W, especially in primary prevention trials^82,83^W are less likely to receive high-intensity statin therapy and to reach LDL-C levels <100 mg/dL compared with M^84–87^ |
| Thrombolytic drugs | - Pooled analysis of 3 RCT (n=2179; 44% W): W with acute ischemic stroke benefit from rtPA more than M^88^ - Non-ST elevation ACS undergoing PCI: the benefit with abciximab is greater in M than in W^89^ - STEMI undergoing PCI: abciximab significantly reduced ischemic events, 30 day and 1 year mortality in W, but not in M^90^ - Contradictory results garding the risk of morbidity and mortality in W and M in the setting of thrombolysis^91–93^ |
| Warfarin | - Enhanced dose-response to warfarin in W, due to lower mean BMI and SRD in warfarin metabolism via P450 enzymes^38,94^ - Significantly higher free plasma levels in W; they require lower doses of warfarin than M to maintain the INR^95–97^ - Meta-analysis (6 RCT; n=63,602; 28% W): W with AF on warfarin were at higher residual risk of CVA/SE than M (P=0.001)^98^ - W treated with warfarin spend more time outside the therapeutic range than M^95^ |

Abbreviations. AA: African American. ACEI: angiotensin-converting enzyme inhibitors. ACS: acute coronary syndrome. ADR: adverse drug reactions. AF: atrial fibrillation. AMI: acute myocardial infarction. aPPT: activated partial thromboplastin time. ARB: angiotensin AT1 receptor blockers. BMI: body mass index. BP: blood pressure. Cl: clearance. CPG: clinical practice guidelines. crCl: creatinine clearance. CV: cardiovascular. CVA/SE: cerebrovascular accident/systemic embolism. CVOT: cardiovascular outcome trial. CYP: cytochrome P450. DOAC: direct oral anticoagulants. ET-1: endothelin-1. GLP-1RAs: glucagon-like peptide-1 receptor agonists. HF: heart failure. HFH: heart failure hospitalization. HFpEF/HFrEF: heart failure with preserved/reduced ejection fraction. HR: hazard ratio. ICH: intracerebral hemorrhage. INR: international normalised ratio. LDL-C: low-density lipoprotein-cholesterol. LV: left ventricular. M: men. MACCE: major adverse cardiac and cerebrovascular events. MACE: major cardiovascular events MI: myocardial infarction. MRA: mineralocorticoid receptor antagonists. 6MWD: 6-minute walking distance. NST-ACS: non-ST segment elevation ACS. OC: oral contraceptive. PCI: percutaneous coronary intervention. PD: pharmacodynamic. P-gp: P-glycoprotein. RCT: randomized clinical trials. rtPA: recombinant tissue plasminogen activator. sCr: serum creatinine. SDC: serum digoxin concentrations. SEE: systemic embolism. SGLT2: sodium-glucose cotransporter-2. SRD: sex-related differences. STEMI: ST-elevation MI. TD: thiazide diuretic. T2D: type 2 diabetes. TIA: transient ischemic attack. UFH: unfractionated heparin. Vd: volume of distribution. VTE: venous thromboembolism. W: women.

**Supplemental Table 3. Sex-related differences in adverse drug reactions of cardiovascular drugs**

| **Drug class** | **Differences in adverse drug reactions between women and men** |
| --- | --- |
| Angiotensin- converting enzyme inhibitors* | - Cough, dysgeusia, skin rash, increase in sCr, gastrointestinal upset and angioedema are more frequent in W^99–101^. W are less persistent on ACEI than M in all age groups^38,102^. W discontinue ACEI due to cough, M because of hypotension |
| Antiarrhythmics | - W present a higher risk for developing QT-related arrhythmias; up to two thirds of torsades de pointes occurred in W^8,9,103,104^ - Procainamide-induced lupus erythematosus is more common in W^105^ |
| Anticoagulants | - Increased risk of bleeding in W than in M^19,31,95,106^. W seemed more prone to be hospitalized with anaemia^52^ - Low molecular weight heparin-induced thrombocytopenia is more frequent in W than M^107^ |
| Antiplatelets | - Increased risk of bleeding in W than in M^25,31^. Bleeding remain higher in W after dose adaptation to BMI and sCr^108^ - W are more likely to receive higher doses of GP IIb/IIIa inhibitors and are at higher risk of bleeding than M^108–110^ - Cangrelor increases the risk of GUSTO moderate bleeding in W but not in M (P_interaction_=0.04)^111^ |
| Angiotensin receptor blockers* | - No SRD in hyperkalaemia, or hypotension^3^ but the incidence of angioedema was higher in M^112^ - In W, replace ACEI by ARB to reduce the risk of ADR^2,3^ - W appear to be more persistent to ARB than to ACEI, probably because they produced less ADR^102^ |
| Aspirin | - Increased bleeding risk in W than in M^113,114^ - The risk of hospitalizations for bleeding due to salicylates^52^ and NSAID-related ulcer complications is higher in M^115,116^ |
| Beta-blockers | - ADR caused by CYP2D6-dependent β-blockers are more frequent in W; they may benefit from lower doses than M^36^ - Metoprolol and propranolol produce a greater reduction in blood pressure and heart rate during exercise in W than in M^39,117^ |
| Calcium channel blockers | - Peripheral edema, potentially leading to decreased adherence is more frequent in W^48,53,118^ - Flushing, headache and ankle edema are more common in W, particularly in elderly W^48,118^ |
| Digoxin | - Higher mortality in W than in M when SDC ≥1.2 ng/mL (P_interaction_=0.034)^50,51^. SDC ≤0.9 ng/mL are recommended in W with HFeEF to reduce mortality^119^ - W are at higher risk of cardiac hospitalizations^52,120^ |
| Diuretics (thiazide and loop) | - W experience more hypo-osmolarity and hospitalizations due to hyponatremia and hypokalemia^52,53,56,121,122^ - W are less likely to experience hyperuricemia and gout in response to thiazide diuretics than M^2,123^ |
| Direct oral anticoagulants | - AF: major bleeding was less frequent in W than in M^63,64,98,124^ - VTE (17 RCT, n= 25,789; 37.6-43.7% W): W had a higher incidence of major bleedings plus clinically relevant minor bleedings than M^66,67^ |
| GLP-1 receptor agonists | - W experienced significantly higher incidence of ADR than M^70^ |
| Isosorbide dinitrate | - Doses should be based on body weight^71^ |
| Isosorbide dinitrate/ hydralazine | - Headache, dizziness and systemic lupus erythematosus are more common in W^125,126^ |
| Ivabradine | - RCT did not report sex-specific ADR data^127^. Vigibase: more non-serious ADR in W^3^ |
| Mineralocorticoid receptor antagonists* | - HFrEF: higher drug discontinuation due to hyperkalaemia, renal impairment, gynecomastia in M than in W^3,53,128^. Eplerenone: produced greater increases in sCr in W^75,129^ - HFrEF: only 1 out of 18 RCT reported sex-specific ADR data^128^ - HFpEF: start MRA if sCr <2.5 mg/dL in M or <2.0 mg/dL in W, and serum potassium <5.0 mEq/L^130^ |
| - Sacubitril-valsartan* | - W are more likely to develop hypotension131 and angioedema132. ADR were not analysed by sex in RCT2,3 |
| - SGLT2 inhibitors | - Higher rates of diabetic ketoacidosis and genitourinary infections in W133,134 and acute renal failure in M70 |
| - Statins | The risk of statin-induced myalgia/myopathy and new-onset diabetes is highest in older W with low BMI^84,85,135–137^W are more likely to discontinue statin therapy because of an ADR than M^84,85,136^ |
| Thrombolytic drugs | - AMI: W present a higher risk of death, reinfarction, stroke, and haemorrhagic stroke than M^31,89,91,92,138,139^ - Bleeding risk is only partly reduced after dose adjustement for BMI and sCr, suggesting the involvement of PD mechanisms^140^ |
| Warfarin | - Higher risk of bleeding in W than M^141,142^ - W need less warfarin per week than M; dose requirements decrease greatly with age, particularly in elderly W^95^ |

^*^ They can cause foetal harm and teratogenic effects: avoid in W during childbearing years; contraindicated during pregnancy

Abbreviations. ACEI: angiotensin-converting enzyme inhibitors. ADR: adverse drug reactions. AF: atrial fibrillation. AMI: acute myocardial infarction. ARB: angiotensin AT1 receptor blockers. BMI: body mass index. CYP: cytochrome P450. DIG: Digitalis Investigation Group. GLP-1: glucagon-like peptide-1 receptor agonists. GP: glycoprotein. GUSTO: Global Utilization of Streptokinase and t-PA for Occluded Coronary Arteries. HFpEF/HFrEF: heart failure with preserved/reduced ejection fraction. M: men. MRA: mineralocorticoid receptor antagonists. NSAID: non-steroidal anti-inflmammatory drugs. RCT: randomized clinical trials. sCr: serum creatinine. SDC: serum digoxin concentrations. SRD: sex-related differences. VTE: venous thromboembolism. W: women.

**Supplemental Table 4. Representation of women and sex-specific analysis of drug efficacy and safety in cardiovascular drug trials**

| **Study** | **Results** |
| --- | --- |
| **A. Representation of women in RCT** | |
| SBA in the Cochrane reviews^143^ | In 258 reviews, W comprised only 27% of the pooled population |
| NHLBI-sponsored CV RCT from 1997 to 2006^144^ | In 19 trials, mean enrollment of W was 27% |
| 2007 AHA guidelines for CVD prevention^60^ | In 156 trials (n=801,198)**,** W representation was highest in hypertension (44%), DM (40%) and stroke (38%), lowest in HF (29%), CAD (25%) and hyperlipidemia (28%) trials |
| RCT published in 9 prominent medical journals in 2009^145^ | Median enrollment of W in 56 RCT was 37%; in 9 studies, less than 20% of W were enrolled |
| RCT cited in the ACC/AHA guidelines for AF (2006), HF (2009), and ACS (2011)^146^ | In 653 trials (n=1,336,922), W represented 33% in AF and 29% in ACS and HF trials, which was lower than that of US registries of AF (55%), ACS (42%) and HF (47%) |
| Publicationsin the three leading medical journals (1997-2009)^147^ | 325 CV trials recruiting more than 1.2 million patients. Enrollment rates of W: 27% in trials of CAD, 27% in HF and 31% in arrhythmia. Between 1997 and 2009 W enrollment rate increased from 27% to 32% |
| ESC-HF-LT registry^148^ | W were only 28.8% of the registry patients (n=12,440) |
| RCT and observational studies published in 11 peer-reviewed journals in 2013^149^ | In 264 RCT and observational studies, W represented 29% of the participants |
| RCT in HF trials published between 1985 and 2016^1^ | Analysis of 22 RCT (n=72,898). In HFrEF trials W represented 20-32% of participants; in HFpEF trials, W represented 40-60% of participants |
| Most-Cited RCT of Cardiology between 1996 and 2015^150^ | The median percentage of W in trials was 28.6% (22.2%–40.5%) and only 14% of trials (n=70) enrolled >50% W |
| Review of sex differences in cardiovascular epigenetics^151^ | In 75 publications, 24 (86%) studied males, and 4 (14%) studied females |
| RCT supporting FDA approval of 36 drugs from 2005 to 2015^152^ | W represented 34% of 174,709 participants. The PPR by CVD was ≥0.8 for AF (0.8-1.1), hypertension (0.9), and PAH (1.4) trials; PPR was <0.8 for HF (0.5-0.6), CAD (0.6), and ACS/MI (0.6) trials |
| Review of HF trials published between 2001 and 2016^153^ | 118 trials (n=215,508). HFpEF trials enrolled a higher proportion of W (56%) compared with HFrEF (24%) or AHF (32%). Corresponding weighted proportions in epidemiologic studies were 62%, 29%, and 50%, respectively. |
| Pivotal RCT with DOAC in AF^64^ | In 5 RCT (n=66,389 patients) W represented 37.8% of the participants |
| CV RCT published in high-IF journals between 1986 and 2015^154^ | In 598 trials (n=2,965,314), W enrollment varied from 37% for non-CAD, 30% for CAD, 28% for HF and 28% for arrhythmia trials, significantly lower than the expected proportion in disease populations. W enrollment increased over time from 21% to 33% |
| RCT cited in the ESC 2016 HF guidelines^127^ | Across 23 trials (n=101,564 participants) W represented 25.2% of participants |
| CV clinical trials published in 6 major medical and CV journal over 2 decades^155^ | In 616 RCT published between 2000 and 2015, the proportion of W in CV trials increased from 28.5% to 35.8% (*P* < 0.001), but varied among different CVD: from 25.5% in CAD and 27.3% in HF, to 51.9% in hypertension |
| Pivotal RCT supporting FDA approval of 35 new cardiometabolic drugs (2008-2017)^156^ | W represented 36% of participants (n=296,163). They were underrepresented in CAD (PPR 0.52), HF (0.58), and ACS 0.68) trials, and overrepresented in PAH trials (1.35). W enrollment did not increase over this study period |
| Systematic review of 740 CVD trials (2010-2017)^157^ | W represented 38.2% of participants (n=862,652). W were reasonably represented in hypertension (PPR 0.82) and PAH trials (1.33), but underrepresented in arrhythmia (0.78), CAD (0.67), stroke (0.73), ACS (0.66) and HF (0.48) trials |
| RCT with lipid-lowering drugs (1990-2018)^158^ | In 60 RCT (n=485,409; 28.5% W). W were underrepresented in lipid RCTs of DM (PPR, 0.74), HF (0.27), stable CAD (0.48), and ACS (0.51). W enrollment increased from 1990-1994 vs 2015-2018 (19.5% vs 33.6%) |
| Inclisiran in patients with hypercholesterolemia^159^ | ORION-10 and ORION-11 trials (n=3,184): only 935 patients were W (29%, PPR 0.63) |
| HFrEF trials published in high-IF journals (2000-2019)^160^ | In 317 RCT (n=183,097), W represented 25.5% of patients. W were under-enrolled in 71.6% of trials; 40.5% of trials enrolled ≤20% of W. Enrollment did not increase significantly between 2000-2019. |
| Representation in stroke trials (1990-2020)^161^ | Among 281 trials (n=588,887), W were underrepresented (37,4%), particularly in trials of intracerebral hemorrhage (PPR 0.73) |
| Sacubitril-vasartan in HFrEF trials^131^ | Meta-analysis of 8 RCT (n= 8,981): only 32.2% were W |
| Antiplatelet and anticoagulant landmark trials (1988-2019)^90^ | 39 trials (n=447,496) with aspirin, clopidogrel, ticagrelor, prasugrel, warfarin, apixaban, and rivaroxaban. W represented 30.1% of participants |
| HF trials (1986-2020)^90^ | In 30 RCT (n=118,282 participants) W represented 24.1% of participants |
| HF RCT published between 2000 and 2020^162^ | In 224 HF RCT (n=228,801) published in journals with an IF ≥10, W represented 28.2% of patients |
| US trials of common vascular diseases (2008-2020)^163^ | 97 trials (n=41,622). W represented 27.5% of patients and were under-represented for all studied conditions: CAS (PPR 0.73), PAD (0.65), TAA/AAA (0.59) and TBAD. W participation did not improve since 2008 |
| Antihypertensive trials until May 2020^164^ | In 2046 studies with the 5 major antihypertensive drugs (n=1,348,172), W represented 38.1% of patients |
| Late-Breaking CV RCT presented at the 2021 ACC/AHA/ESC meetings^165^ | In 68 trials, inclusion of W ranged from 0% to 71%, with a mean of 35.2% . |
| 2023 ACS guidelines of the ESC^166^ | W represent 20%–30% of patients in RCTs of ACS |
| Publications cited in the 2019 ESC guideline on chronic coronary syndromes^167^ | In 108 articles published between 1991 and 2019 (n=1,613,000), only 26.8% (432,284) were W |
| SGLT2 inhibitors and HF RCT published between 2018-2024^168^ | In 43 RCT (n=27,703) the overall proportion of W enrolled in the studies was 35.6% |
| **B. Sex-specific analysis of drug efficacy and safety** | |
| Cochrane Reviews related to treatment of CVD^143^ | In 196 trials, only 33% performed a SBA of outcomes. In trials that performed SBA, 20% reported significant sex-related differences in CV-related outcomes |
| 2007 AHA guidelines for CVD prevention^60^ | In 156 RCT, sex-specific results were discussed in only 31% of primary trial publications |
| RCTs published in 9 prominent medical journals in 2009^145^ | In 86 trials, 75% of the studies did not report any outcomes by sex |
| SBA in 38 Cochrane systematic reviews on CVD from 2001 to 2007^169^ | Only 2 reviews reported any sex research gaps; only one quarter included a rationale as to why any subgroup analyses by sex were or were not completed. None met all the appraisal tool criteria. |
| RCT published in high-IF journals (2008-2013)^170^ | In 57 randomly selected federally funded RCT (43% W), the minority of trials (22%) that did analyse sex differences did not discuss or reflect upon these, or dismissed significant findings |
| RCT performed in Canada in 2013-2014^171^ | 100 RCT: 6% performed a SBA; 4% reported sex-disaggregated data; 1 discussed the implications of the findings for clinical practice |
| Analysis of 133 CaRs and 555 CoRs from 2016 to 2017^172^ | In the results section, less than 30% of reviews reported on sex. 37% of CaRs and 75% of CoRs provided a descriptive report of sex and 63% and 25% reported analytic approaches for exploring sex (subgroup analyses or presented sex-disaggregated) |
| Review of sex differences in cardiovascular epigenetics^151^ | In 75 publications only 13 (17%) stratified some of their data according to sex |
| Sex-sensitive review behind current HF CPG^1^ | HF RCT cited in CPG in Europe, US, and Canada were not powered to detect SRD, to test the benefit in W, or to identify a drug that would only be effective in W |
| RCT cited in the ESC 2016 HF guidelines^127,173^ | Of 155 trials (n=153,945), only 11 (7%) reported sex-specific ADR data |
|  | 23 RCT (n=101,564; 25% W): 48% reported sex-specific efficacy data, and only 2 studies (9%) presented sex-specific information about ADR |
| RCT with lipid-lowering therapies (1990-2018)^158^ | In 60 RCT (485,409 participants, 28.5% W). Only53.0% of these trials reported outcomes by sex, with no significant improvement over time |
|  | In 60 RCT (n=485,409; 28.5% W). W were underrepresented in lipid RCTs of DM (PPR 0.74), HF (0.27), stable CAD (0.48), and ACS (0.51). W enrollment increased from 1990-1994 vs 2015-2018 (19.5% vs 33.6%) |
| Novel cardiometabolic drug approvals from 2008 to 2017^156^ | 143 trials (n=296,163): 57 CV and 86 DM (296,163 participants; 36% W). W were underrepresented in trials of CAD (PPR 0.52), HF (0.58), and ACS (0.68), but overrepresented in PAH trials (1.35) |
| HF RCT published in 9 high-IF journals between 2008 and 2017^174^ | Of 261 HF trials, 107 (41%) reported subgroup analyses, credibility of subgroup claims was generally low across all strengths of claims |
| RCT cited by the 2018 ESH-ESC guidelines^175^ | 33 RCT with >1,000 subjects (305,249 participants; 44,38% W). Only 11 trials reported the results according to sex; 7 did it in a subsequent publication devoted to planned subgroup analysis |
| RCT presented between 2010 and 2017 in the ESC/AHA/ACC sessions and published in high-IF journals^176^ | In 63 RCT, sex-specific efficacy endpoints were reported for 34.5% of trials in 2010 and 23.5% in 2017; sex-specific safety outcomes in 11.1% and 8.6%, respectively |
| Sex-specific reporting in HF RCT published between 2000 and 2020^162^ | In 224 RCT (n=228,801), no trial reported sex-disaggregated screening, consent or withdrawal rates or sex-specific ADR; 75 RCT (33.4%) presented sex subgroup analysis, and 63 (28.3%) reported sex-treatment interaction |
| Trials on antihypertensive drugs until May 2020^177^ | Among 2,046 studies (n=1,348,172 adults; 30.1% W), only 75 (3.7%) studies performed sex stratification, and this was the highest between 2011 and 2020 (7.2%). |
| Late-Breaking CV RCT presented at the 2021 ACC/AHA/ESC meetings^165^ | The average PPR was 0.76 for all trials, but varied based on subspecialty, with a statistical difference between interventional cardiology and HF (0.65 vs. 0.88). |
| Acute care trials published in high-IF journals^178^ | 88 trials (75 ICU and 13 cardiology trials; n=111,428; 34.2% W). Only 23 (26.1%) trials reported a SBA |
| References cited in the 5 major CPG of hypertension^179^ | Among 331 trials, 81% reported the sex of participants, and 22% a PPR of 0.8-1.2; 3% of trials stratified baseline characteristics by sex, and 20% considered sex during analysis through statistical adjustment or stratification. Only 0.6% stratified ADR by sex. |
| Publications cited in the 2019 ESC guideline on chronic coronary syndromes^167^ | Of 108 trials published between 1991 and 2019, only 3 incorporated sex-sensitive designs. Post-hoc, sex-specific analyses were found only in 13% of the publications |

Abbreviations. ACC: American College of Cardiology. ACS: acute coronary syndrome. ADR: adverse drug reactions. AF: atrial fibrillation. AHA: American Heart Association. AHF: acute heart failure. CAD: coronary artery disease. CaRs: Campbell reviews. CAS: carotid artery stenosis. CoRs: Cochrane reviews. CPG: clinical practice guidelines. CV: cardiovascular. CVD: cardiovascular disease. DM: diabetes mellitus. DOAC. Direct oral anticoagulants. ESC: European Society of Cardiology. ESC-HF-LT: European Society of Cardiology-Heart Failure Long-Term Registry. ESH: European Society of Hypertension. FDA: US Food and Drug Administration. HF: heart failure. HFrEF/HFpEF: heart failure with reduced/preserved ejection fraction. ICU: intensive care unit. IF: impact factor. M: men. MI: myocardial infarction. NHLBI: National Heart, Lung, and Blood Institute. PAD: peripheral arterial disease. PAH: pulmonary arterial hypertension. PPR: participation-to-prevalence ratio (a PPR of 0.8–1.2 suggests an adequate representation of W in trials relative to disease population; a PPR <0.8 under-representation, and a PPR >1.2 over-representation). RCT: randomized clinical trials. SBA: sex-based analysis. SGLT2: SGLT2: sodium-glucose cotransporter-2. SRD: sex-related differences. TAA/AAA: thoracic/abdominal aortic aneurysms. TBAD: type B aortic dissections. W. women.

**Supplemental Table 5. Consequences of women under-representation and lack of sex-specific analysis and report of drug efficacy and safety in RCT with cardiovascular drugs**

| 1. Severely limited our ability to recognize the existence of SRD even if they exist and to understand their clinical relevance^26,38,145,180,181^ |
| --- |
| 1. RCT where the inclusion of W does not mirror the prevalence in the general population (PPR <0.8) have important consequences^152,157,182–185^: |
| - They suffer from sample selection bias as W are considered as a subgroup |
| - Do not reproduce real-world practice and are not representative of the population to treat |
| - Results obtained mainly in middle-aged men cannot be extrapolated to the population that was not adequately represented, i.e. W^1,3,145,150,152,186^ |
| - The efficacy and safety of some CV drugs remains uncertain in W |
| - Reduce the ability to indentify sex-specific differences in important outcomes |
| 3. CPG based on RCT recruiting mainly middle-aged men frequently conclude that there are no SRD |
| - Recommend (with few exceptions) the same treatment and doses for both M and W despite the important SRD in drug safety |
| - Recommendations may be more suitable for M, leading to suboptimal care for W with CVD |
| - Clinicians do not have evidence-based information to guide W healthcare, potentially leading to suboptimal outcomes, or alternatively, exposing them to frequent adverse drug reactions |
| 1. Hinders the ability to develop sex-specific strategies that could improve CPG recommendations for the prevention and treatment of CVD and to reduce inequities in healthcare in W^162,182,183,187,188^ |
| 1. The limited information on SRD is the result of RCT unpowered to detect such differences, rather than a true absence of differences |

Abbreviations. CPG: clinical practice guidelines. CV: cardiovascular. CVD: cardiovascular disease. M: men. MI: myocardial infarction. PPR: participation-to-prevalence ratio. RCT: randomized clinical trials. SRD: sex -related differences. W: women.

**Supplemental Table 6. Sex-related differences in the prescription of cardiovascular drugs**

| **Disease/drug** | **Differences between women and men** |
| --- | --- |
| Acute coronary syndromes | - W were less likely to be prescribed β-blockers, ACEI or ARB, statins, and antithrombotics (heparins, dual antiplatelet therapy, thrombolysis) and less commonly received aspirin, ACEI, and statins at discharge^189–195^ |
| Antihypertensive drugs | - W were prescribed more thiazide and loop diuretics, β-blockers and ARB, but less frequently ACEI, calcium channel blockers or β-blockers than M despite comparable blood pressure levels^2,196–202^. The use of 2-3 antihypertensives is less common in W^198^ |
| Atrial fibrillation | - W used more often rate-control drugs (β-blockers and digoxin) and M rhythm-control drugs (class I and III antiarrhythmics)^203,204^ - W were significantly less likely than M to use any oral anticoagulant overall and at all levels of CHA_2_DS_2_-VASc score and DOAC were more frequently underdosed despite they have a higher risk of fatal or disabling stroke than M^205–207^ |
| Coronary artery disease | - W were significantly less likely to be treated with antithrombotics, β-blockers and lipid-lowering drugs (statins) than M^189,208^ - W less likely to receive CPGRD for CAD or MI^209^ |
| Cardiogenic shock | - W were less likely to receive CPGRD ([aspirin](https://www.sciencedirect.com/topics/medicine-and-dentistry/acetylsalicylic-acid), P2Y_12_ receptor inhibitors, β-blockers, ACEI/ARB, [statin](https://www.sciencedirect.com/topics/pharmacology-toxicology-and-pharmaceutical-science/statin-protein)s) or any parenteral [anticoagulation](https://www.sciencedirect.com/topics/medicine-and-dentistry/anticoagulation) during the hospitalization and were less likely to be discharged on CPGRD^210^ |
| Diabetes and CVD | - W were less likely to receive CPGRD for patients with diabetes and cardiovascular conditions^209^ |
| Diabetes and HF | - W received more β-blockers, ARBs, and diuretics, but less ACEI/ARB than M^198^ |
| DOAC | - W were more frequently treated with low-dose dabigatran compared with M^211^ |
| Heart failure | - HFrEF: W received more diuretics, ARB and digoxin, but were less frequently treated with ACEI, MRA or β-blockers and, when prescribed, doses were lower than those for M^3,209,212–217^ - HFmrEF and HFpEF: lower prescription rate of ACEI/ARB in W^218^ - HFpEF: W received more diuretics and MRA, but were less likely to receive digoxin or β-blockers^218,219^ - Acute HF: W were less likely to receive vasoactive therapy and β-blockers and ACEI/ARB or MRA at discharge^220^ |
| Hyperlipidemia | - W with hypercholesterolaemia are less likely to receive high-intensity statin therapy and to reach LDL-C targets than M^221–224^ |
| Hypertension and CAD | - Swedish Primary Care Cardiovascular Database: W received more ARB and MRA; ACEI were more frequently prescribed in M^197^ |
| Hypertension and CKD | - The overall use of ACEI, β-blockers and calcium channel blockers was lower, while the use of ARB was higher in W than in M^225^ |
| Warfarin | - Meta-analysis of 28 observational studies: W were significantly less likely to receive warfarin than M^226^ |
| Patients with established CVD | - Meta-analysis of 43 studies (2,264,600 participants; 28% W): W were less likely to be prescribed aspirin, statins or ACEI, but were more likely to receive diuretics. No SRD in the prescription of β-blockers and calcium channel blockers^227^ |

Abbreviations. ACEI: angiotensin-converting enzyme-inhibitors. ARB: angiotensin receptor blockers. CAD: coronary artery disease. CKD: chronic kidney disease. CPGRD: clinical practice guidelines recommended drugs. CVD: cardiovascular disease. DOAC: direct oral anticoagulants. HFmrEF/HFpEF/HFrEF: heart failure with midly reduced/preserved/reduced ejection fraction. LDL-C: low-density lipoprotein-cholesterol. M: men. MI: myocardial infarction. MRA: mineralocorticoid receptor antagonists. SRD: sex-related differences. W: women.

**Supplemental Table 7**. Main limitations of this narrative review

| 1. Limited information from prospective RCT: information came mainly from subgroup and post-hoc analysis and meta-analyses |
| --- |
| 1. In many RCT:  - Women were underrepresented (selection bias) and trials underpowered to perform sex-specific analyses - Randomization was not stratified based on sex or gender and used both terms intercangeably - No information was available on baseline characteristics (menopausal status, contraceptive use, hormone replacement therapy), risk factors, or relevant comorbidities unique for W - Efficacy and safety of cardiovascular drugs were not reported in a sex-disaggregated manner |
| 1. Analysis were based on published aggregate data, not individual patient-level data, which would have allowed for better adjustment for confounding factors |
| 1. Some subanalysis present important limitations:  - Non-pre-specified, inadequate statistical power (false negatives), lack of adjustment for confounders, multiple comparisons (false positives), and lack of formal interaction testing |
| 1. Some systematic meta-analysis:  - Were non-pre-specified or adjusted for multiplicity to avoid spurious conclusions - RCT may not be poolable because of significant heterogeneity in study design, baseline characteristics, drug types, follow-up and outcome measures (risk of misleading interpretations) |
| 1. The search was limited to publications in English from Western countries (which may limit the generalizability) and to articles published in prominent medical journals (potential publication bias) |

Abbreviations. RCT: randomized clinical trials. W: women.

**Supplemental Table 8. Gaps in knowledge and steps to improve our understanding of SRD in dosing, pharmacokinetics and pharmacodynamics (efficacy and safety) of CV drugs^183–185,228–235^**

| **Present gaps** | **Next steps** |
| --- | --- |
| Poor awareness of CVD | - Develop educational programmes among patients, health-care providers, and policymakers to increase awareness that CVD represent the leading cause of death among W |
| The mechanisms underlying the SRD remains uncertain | - Evaluate the potential of SRD in CV drug efficacy and safety at each step of drug development - A deeper understanding of the mechanisms behind these SRD is needed to provide the best clinical health-care and minimize the higher incidence of ADR in women - Increase funding supporting basic and clinical research of SRD |
| Underrepresentation of W in CV RCT | - Develop effective strategies (including digital tools) to improve recruitment and retention of W in CV RCT - Identify and remove barriers and unnecessary exclusion criteria limiting women enrollment |
| Study design does not allow to report SDR | - Prospective RCT should be specifically designed to analyse and report potential differences in dosing, efficacy, and safety of CV drugs for W and M separately, and to establish appropriate management strategies for women. - Outcomes particularly important for W should be considered in the design of RCT - Prioritize research funding in CVD specific to W or predominantly affecting W - Regulatory agencies, scientific journals, and funding agencies should require the analysis and report of sex-stratified results· |
| Underuse of CPGRD in women | - Increasing the prescription and adherence to CPGRD for the prevention and treatment of CVD represents the most efficient way to improve clinical outcomes in W |
| Define the optimal doses | - Early drug development programs should define sex-appropriate dosing - Support prospective RCT to define the optimal doses of CPGRD in W and M |
| Poor translation of SRD into clinical practice | - Evidence-based SRD should be considered when selecting the drug and dosage - SRD in CV drug efficacy and safety should be incorporated in CPG, medical eductation, drug labels, and websites |
| Lack of sex-specific CPG | - Developing and implementing sex-specific CPG for CV prevention and treatment is an effective strategy to improve clinical outcomes and minimize the higher incidence of ADR in W |
| Poor communication between health-care providers | - Prevention and management of CVD in W require a multidisciplinary care approach involving cardiologists and other health professionals (obstetricians and gynecologists, among others) throughout a woman's life |
| Improve education | - Education on SRD in cardiovascular drug efficacy and safety should be embedded in the medical curriculum and reinforced in continuing medical education programs |

Abbreviations. ADR: adverse drug reactions. CV: cardiovascular. CVD: cardiovascular disease. CPG: clinical practice guidelines. CPGRD: clinical practice guidelines recommended drugs. RCT: randomized clinical trials. SRD: sex-related differences. W: women.

**Referencias**

1. Levinsson A, Dubé M-P, Tardif J-C, Denus S de. Sex, drugs, and heart failure: a sex-sensitive review of the evidence base behind current heart failure clinical guidelines. *ESC Heart Fail* 2018;**5**:745–754.

2. Tamargo J, Caballero R, Mosquera ED. Sex and gender differences in the treatment of arterial hypertension. *Expert Rev Clin Pharmacol* 2023;**16**:329–347.

3. Tamargo J, Caballero R, Delpón E. Sex-related differences in the pharmacological treatment of heart failure. *Pharmacol Ther* 2022;**229**:107891.

4. Garg R, Yusuf S. Overview of randomized trials of angiotensin-converting enzyme inhibitors on mortality and morbidity in patients with heart failure. Collaborative Group on ACE Inhibitor Trials. *JAMA* 1995;**273**:1450–1456.

5. Shekelle PG, Rich MW, Morton SC, Atkinson CSW, Tu W, Maglione M, Rhodes S, Barrett M, Fonarow GC, Greenberg B, Heidenreich PA, Knabel T, Konstam MA, Steimle A, Warner Stevenson L. Efficacy of angiotensin-converting enzyme inhibitors and beta-blockers in the management of left ventricular systolic dysfunction according to race, gender, and diabetic status: a meta-analysis of major clinical trials. *J Am Coll Cardiol* 2003;**41**:1529–1538.

6. Packer M, Poole-Wilson PA, Armstrong PW, Cleland JG, Horowitz JD, Massie BM, Rydén L, Thygesen K, Uretsky BF. Comparative effects of low and high doses of the angiotensin-converting enzyme inhibitor, lisinopril, on morbidity and mortality in chronic heart failure. ATLAS Study Group. *Circulation* 1999;**100**:2312–2318.

7. Santema BT, Ouwerkerk W, Tromp J, Sama IE, Ravera A, Regitz-Zagrosek V, Hillege H, Samani NJ, Zannad F, Dickstein K, Lang CC, Cleland JG, Ter Maaten JM, Metra M, Anker SD, Harst P van der, Ng LL, Meer P van der, Veldhuisen DJ van, Meyer S, Lam CSP, ASIAN-HF investigators, Voors AA. Identifying optimal doses of heart failure medications in men compared with women: a prospective, observational, cohort study. *Lancet* 2019;**394**:1254–1263.

8. Peters RW, Gold MR. The influence of gender on arrhythmias. *Cardiol Rev* 2004;**12**:97–105.

9. Schwartz PJ, Woosley RL. Predicting the Unpredictable: Drug-Induced QT Prolongation and Torsades de Pointes. *J Am Coll Cardiol* 2016;**67**:1639–1650.

10. Koup JR, Abel RB, Smithers JA, Eldon MA, Vries TM de. Effect of age, gender, and race on steady state procainamide pharmacokinetics after administration of procanbid sustained-release tablets. *Ther Drug Monit* 1998;**20**:73–77.

11. Routledge PA, Stargel WW, Kitchell BB, Barchowsky A, Shand DG. Sex-related differences in the plasma protein binding of lignocaine and diazepam. *Br J Clin Pharmacol* 1981;**11**:245–250.

12. Jick H, Slone D, Borda IT, Shapiro S. Efficacy and toxicity of heparin in relation to age and sex. *N Engl J Med* 1968;**279**:284–286.

13. Cipolle RJ, Seifert RD, Neilan BA, Zaske DE, Haus E. Heparin kinetics: variables related to disposition and dosage. *Clin Pharmacol Ther* 1981;**29**:387–393.

14. Campbell NR, Hull RD, Brant R, Hogan DB, Pineo GF, Raskob GE. Different effects of heparin in males and females. *Clin Invest Med* 1998;**21**:71–78.

15. Granger CB, Hirsch J, Califf RM, Col J, White HD, Betriu A, Woodlief LH, Lee KL, Bovill EG, Simes RJ, Topol EJ. Activated partial thromboplastin time and outcome after thrombolytic therapy for acute myocardial infarction: results from the GUSTO-I trial. *Circulation* 1996;**93**:870–878.

16. Cohen M, Antman EM, Gurfinkel EP, Radley D, ESSENCE (Efficacy and Safety of Subcutaneous Enoxaparin in Non-Q-wave Coronary Events) and TIMI (Thrombolysis in Myocardial Infarction) 11B Investigators. Enoxaparin in unstable angina/non-ST-segment elevation myocardial infarction: treatment benefits in prespecified subgroups. *J Thromb Thrombolysis* 2001;**12**:199–206.

17. Toss H, Wallentin L, Siegbahn A. Influences of sex and smoking habits on anticoagulant activity in low-molecular-weight heparin treatment of unstable coronary artery disease. *Am Heart J* 1999;**137**:72–78.

18. Roosendaal LC, Wiersema AM, Smit JW, Doganer O, Blankensteijn JD, Jongkind V. Editor’s Choice - Sex Differences in Response to Administration of Heparin During Non-Cardiac Arterial Procedures. *Eur J Vasc Endovasc Surg* 2022;**64**:557–565.

19. Ng VG, Baumbach A, Grinfeld L, Lincoff AM, Mehran R, Stone GW, Lansky AJ. Impact of Bleeding and Bivalirudin Therapy on Mortality Risk in Women Undergoing Percutaneous Coronary Intervention (from the REPLACE-2, ACUITY, and HORIZONS-AMI Trials). *Am J Cardiol* 2016;**117**:186–191.

20. Chacko M, Lincoff AM, Wolski KE, Cohen DJ, Bittl JA, Lansky AJ, Tsuchiya Y, Betriu A, Yen MH, Chew DP, Cho L, Topol EJ. Ischemic and bleeding outcomes in women treated with bivalirudin during percutaneous coronary intervention: a subgroup analysis of the Randomized Evaluation in PCI Linking Angiomax to Reduced Clinical Events (REPLACE)-2 trial. *Am Heart J* 2006;**151**:1032.e1-7.

21. Teng R. Ticagrelor: Pharmacokinetic, Pharmacodynamic and Pharmacogenetic Profile: An Update. *Clin Pharmacokinet* 2015;**54**:1125–1138.

22. Agbaedeng TA, Noubiap JJ, Roberts KA, Chew DP, Psaltis PJ, Amare AT. Sex-Based Outcomes of Dual-Antiplatelet Therapy After Percutaneous Coronary Intervention: A Pairwise and Network Meta-Analysis. *Drugs* 2024;**84**:685–701.

23. Berger JS, Bhatt DL, Cannon CP, Chen Z, Jiang L, Jones JB, Mehta SR, Sabatine MS, Steinhubl SR, Topol EJ, Berger PB. The relative efficacy and safety of clopidogrel in women and men a sex-specific collaborative meta-analysis. *J Am Coll Cardiol* 2009;**54**:1935–1945.

24. Boersma E, Harrington RA, Moliterno DJ, White H, Théroux P, Van de Werf F, Torbal A de, Armstrong PW, Wallentin LC, Wilcox RG, Simes J, Califf RM, Topol EJ, Simoons ML. Platelet glycoprotein IIb/IIIa inhibitors in acute coronary syndromes: a meta-analysis of all major randomised clinical trials. *Lancet* 2002;**359**:189–198.

25. Patti G, De Caterina R, Abbate R, Andreotti F, Biasucci LM, Calabrò P, Cioni G, Davì G, Di Sciascio G, Golia E, Golino P, Malatesta G, Mangiacapra F, Marcucci R, Nusca A, Parato VM, Pengo V, Prisco D, Pulcinelli F, Renda G, Ricottini E, Ruggieri B, Santilli F, Sofi F, Zimarino M, Working Group on Thrombosis of the Italian Society of Cardiology. Platelet function and long-term antiplatelet therapy in women: is there a gender-specificity? A ‘state-of-the-art’ paper. *Eur Heart J* 2014;**35**:2213–2223b.

26. Seeland U, Regitz-Zagrosek V. Sex and gender differences in cardiovascular drug therapy. *Handb Exp Pharmacol* 2012:211–236.

27. Rabi DM, Khan N, Vallee M, Hladunewich MA, Tobe SW, Pilote L. Reporting on sex-based analysis in clinical trials of angiotensin-converting enzyme inhibitor and angiotensin receptor blocker efficacy. *Can J Cardiol* 2008;**24**:491–496.

28. Ferreira JP, Konstam MA, McMurray JJV, Butler J, Girerd N, Rossignol P, Sharma A, Voors AA, Lam CSP, Packer M, Zannad F. Dosing of losartan in men versus women with heart failure with reduced ejection fraction: the HEAAL trial. *Eur J Heart Fail* 2021;**23**:1477–1484.

29. Miners JO, Grgurinovich N, Whitehead AG, Robson RA, Birkett DJ. Influence of gender and oral contraceptive steroids on the metabolism of salicylic acid and acetylsalicylic acid. *Br J Clin Pharmacol* 1986;**22**:135–142.

30. Ho PC, Triggs EJ, Bourne DW, Heazlewood VJ. The effects of age and sex on the disposition of acetylsalicylic acid and its metabolites. *Br J Clin Pharmacol* 1985;**19**:675–684.

31. Capodanno D, Angiolillo DJ. Impact of race and gender on antithrombotic therapy. *Thromb Haemost* 2010;**104**:471–484.

32. Harrison MJ, Weisblatt E. A sex difference in the effect of aspirin on ‘spontaneous’ platelet aggregation in whole blood. *Thromb Haemost* 1983;**50**:773–774.

33. Gum PA, Kottke-Marchant K, Poggio ED, Gurm H, Welsh PA, Brooks L, Sapp SK, Topol EJ. Profile and prevalence of aspirin resistance in patients with cardiovascular disease. *Am J Cardiol* 2001;**88**:230–235.

34. Cavallari LH, Helgason CM, Brace LD, Viana MAG, Nutescu EA. Sex difference in the antiplatelet effect of aspirin in patients with stroke. *Ann Pharmacother* 2006;**40**:812–817.

35. Yerman T, Gan WQ, Sin DD. The influence of gender on the effects of aspirin in preventing myocardial infarction. *BMC Med* 2007;**5**:29.

36. Thürmann PA, Haack S, Werner U, Szymanski J, Haase G, Drewelow B, Reimann IR, Hippius M, Siegmund W, May K, Hasford J. Tolerability of beta-blockers metabolized via cytochrome P450 2D6 is sex-dependent. *Clin Pharmacol Ther* 2006;**80**:551–553.

37. Walle T, Byington RP, Furberg CD, McIntyre KM, Vokonas PS. Biologic determinants of propranolol disposition: results from 1308 patients in the Beta-Blocker Heart Attack Trial. *Clin Pharmacol Ther* 1985;**38**:509–518.

38. Tamargo J, Rosano G, Walther T, Duarte J, Niessner A, Kaski JC, Ceconi C, Drexel H, Kjeldsen K, Savarese G, Torp-Pedersen C, Atar D, Lewis BS, Agewall S. Gender differences in the effects of cardiovascular drugs. *Eur Heart J Cardiovasc Pharmacother* 2017;**3**:163–182.

39. Luzier AB, Killian A, Wilton JH, Wilson MF, Forrest A, Kazierad DJ. Gender-related effects on metoprolol pharmacokinetics and pharmacodynamics in healthy volunteers. *Clin Pharmacol Ther* 1999;**66**:594–601.

40. Eugene AR. Gender based Dosing of Metoprolol in the Elderly using Population Pharmacokinetic Modeling and Simulations. *Int J Clin Pharmacol Toxicol* 2016;**5**:209–215.

41. Aimo A, Vergaro G, Barison A, Maffei S, Borrelli C, Morrone D, Cameli M, Palazzuoli A, Ambrosio G, Coiro S, Savino K, Cerbai E, Marcucci R, Pedrinelli R, Padeletti L, Passino C, Emdin M. Sex-related differences in chronic heart failure. *Int J Cardiol* 2018;**255**:145–151.

42. Fukuta H, Goto T, Kamiya T. Effects of beta-blocker withdrawal in patients with heart failure with preserved ejection fraction: A protocol for systematic review and meta-analysis. *PLoS One* 2023;**18**:e0294347.

43. Simon T, Mary-Krause M, Funck-Brentano C, Jaillon P. Sex differences in the prognosis of congestive heart failure: results from the Cardiac Insufficiency Bisoprolol Study (CIBIS II). *Circulation* 2001;**103**:375–380.

44. Krecic-Shepard ME, Barnas CR, Slimko J, Jones MP, Schwartz JB. Gender-specific effects on verapamil pharmacokinetics and pharmacodynamics in humans. *J Clin Pharmacol* 2000;**40**:219–230.

45. Krecic-Shepard ME, Park K, Barnas C, Slimko J, Kerwin DR, Schwartz JB. Race and sex influence clearance of nifedipine: results of a population study. *Clin Pharmacol Ther* 2000;**68**:130–142.

46. Schwartz JB, Capili H, Wainer IW. Verapamil stereoisomers during racemic verapamil administration: effects of aging and comparisons to administration of individual stereoisomers. *Clin Pharmacol Ther* 1994;**56**:368–376.

47. Kang D, Verotta D, Krecic-Shepard ME, Modi NB, Gupta SK, Schwartz JB. Population analyses of sustained-release verapamil in patients: effects of sex, race, and smoking. *Clin Pharmacol Ther* 2003;**73**:31–40.

48. Kloner RA, Sowers JR, DiBona GF, Gaffney M, Wein M. Sex- and age-related antihypertensive effects of amlodipine. The Amlodipine Cardiovascular Community Trial Study Group. *Am J Cardiol* 1996;**77**:713–722.

49. Kjeldsen SE, Warnold I, Hansson L, HOT Study Group. Hypertension Optimal Treatment Study Group. Influence of gender on prevention of myocardial infarction by antihypertensives and acetylsalicylic acid: the HOT study. *J Gend Specif Med* 2000;**3**:35–38.

50. Rathore SS, Curtis JP, Wang Y, Bristow MR, Krumholz HM. Association of serum digoxin concentration and outcomes in patients with heart failure. *JAMA* 2003;**289**:871–878.

51. Adams KF, Patterson JH, Gattis WA, O’Connor CM, Lee CR, Schwartz TA, Gheorghiade M. Relationship of serum digoxin concentration to mortality and morbidity in women in the digitalis investigation group trial: a retrospective analysis. *J Am Coll Cardiol* 2005;**46**:497–504.

52. Rodenburg EM, Stricker BHC, Visser LE. Sex-related differences in hospital admissions attributed to adverse drug reactions in the Netherlands. *Br J Clin Pharmacol* 2011;**71**:95–104.

53. Rydberg DM, Mejyr S, Loikas D, Schenck-Gustafsson K, Euler M von, Malmström RE. Sex differences in spontaneous reports on adverse drug events for common antihypertensive drugs. *Eur J Clin Pharmacol* 2018;**74**:1165–1173.

54. Musini VM, Nazer M, Bassett K, Wright JM. Blood pressure-lowering efficacy of monotherapy with thiazide diuretics for primary hypertension. *Cochrane Database Syst Rev* 2014:CD003824.

55. Chapman MD, Hanrahan R, McEwen J, Marley JE. Hyponatraemia and hypokalaemia due to indapamide. *Med J Aust* 2002;**176**:219–221.

56. Sharabi Y, Illan R, Kamari Y, Cohen H, Nadler M, Messerli FH, Grossman E. Diuretic induced hyponatraemia in elderly hypertensive women. *J Hum Hypertens* 2002;**16**:631–635.

57. Werner U, Werner D, Heinbüchner S, Graf B, Ince H, Kische S, Thürmann P, König J, Fromm MF, Zolk O. Gender is an important determinant of the disposition of the loop diuretic torasemide. *J Clin Pharmacol* 2010;**50**:160–168.

58. Gueyffier F, Boutitie F, Boissel JP, Pocock S, Coope J, Cutler J, Ekbom T, Fagard R, Friedman L, Perry M, Prineas R, Schron E. Effect of antihypertensive drug treatment on cardiovascular outcomes in women and men. A meta-analysis of individual patient data from randomized, controlled trials. The INDANA Investigators. *Ann Intern Med* 1997;**126**:761–767.

59. Sanford M, Plosker GL. Dabigatran etexilate. *Drugs* 2008;**68**:1699–1709.

60. Melloni C, Berger JS, Wang TY, Gunes F, Stebbins A, Pieper KS, Dolor RJ, Douglas PS, Mark DB, Newby LK. Representation of women in randomized clinical trials of cardiovascular disease prevention. *Circ Cardiovasc Qual Outcomes* 2010;**3**:135–142.

61. Vinereanu D, Stevens SR, Alexander JH, Al-Khatib SM, Avezum A, Bahit MC, Granger CB, Lopes RD, Halvorsen S, Hanna M, Husted S, Hylek EM, Mărgulescu AD, Wallentin L, Atar D. Clinical outcomes in patients with atrial fibrillation according to sex during anticoagulation with apixaban or warfarin: a secondary analysis of a randomized controlled trial. *Eur Heart J* 2015;**36**:3268–3275.

62. Liao J-N, Huang Y-S, Tsai C-T, Kuo L, Chen S-J, Tuan T-C, Chen T-J, Chen S-A, Chao T-F. Gender Differences in Patients with Atrial Fibrillation Receiving Oral Anticoagulants. *Rev Cardiovasc Med* 2024;**25**:92.

63. Proietti M, Cheli P, Basili S, Mazurek M, Lip GYH. Balancing thromboembolic and bleeding risk with non-vitamin K antagonist oral anticoagulants (NOACs): A systematic review and meta-analysis on gender differences. *Pharmacol Res* 2017;**117**:274–282.

64. Raccah BH, Perlman A, Zwas DR, Hochberg-Klein S, Masarwa R, Muszkat M, Matok I. Gender Differences in Efficacy and Safety of Direct Oral Anticoagulants in Atrial Fibrillation: Systematic Review and Network Meta-analysis. *Ann Pharmacother* 2018;**52**:1135–1142.

65. Law SWY, Lau WCY, Wong ICK, Lip GYH, Mok MT, Siu C-W, Chan EW. Sex-Based Differences in Outcomes of Oral Anticoagulation in Patients With Atrial Fibrillation. *J Am Coll Cardiol* 2018;**72**:271–282.

66. Alotaibi GS, Almodaimegh H, McMurtry MS, Wu C. Do women bleed more than men when prescribed novel oral anticoagulants for venous thromboembolism? A sex-based meta-analysis. *Thromb Res* 2013;**132**:185–189.

67. Loffredo L, Violi F, Perri L. Sex related differences in patients with acute venous thromboembolism treated with new oral anticoagulants. A meta-analysis of the interventional trials. *Int J Cardiol* 2016;**212**:255–258.

68. Gabler NB, French B, Strom BL, Liu Z, Palevsky HI, Taichman DB, Kawut SM, Halpern SD. Race and sex differences in response to endothelin receptor antagonists for pulmonary arterial hypertension. *Chest* 2012;**141**:20–26.

69. Singh AK, Singh R. Gender difference in cardiovascular outcomes with SGLT-2 inhibitors and GLP-1 receptor agonist in type 2 diabetes: A systematic review and meta-analysis of cardio-vascular outcome trials. *Diabetes Metab Syndr* 2020;**14**:181–187.

70. Raparelli V, Elharram M, Moura CS, Abrahamowicz M, Bernatsky S, Behlouli H, Pilote L. Sex Differences in Cardiovascular Effectiveness of Newer Glucose-Lowering Drugs Added to Metformin in Type 2 Diabetes Mellitus. *J Am Heart Assoc* 2020;**9**:e012940.

71. Vree TB, Dammers E, Valducci R. Sex-related differences in the pharmacokinetics of isosorbide-5-mononitrate (60 mg) after repeated oral administration of two different original prolonged release formulations. *Int J Clin Pharmacol Ther* 2004;**42**:463–472.

72. Taylor AL, Lindenfeld J, Ziesche S, Walsh MN, Mitchell JE, Adams K, Tam SW, Ofili E, Sabolinski ML, Worcel M, Cohn JN, A-HeFT Investigators. Outcomes by gender in the African-American Heart Failure Trial. *J Am Coll Cardiol* 2006;**48**:2263–2267.

73. Vasan RS, Evans JC, Benjamin EJ, Levy D, Larson MG, Sundstrom J, Murabito JM, Sam F, Colucci WS, Wilson PWF. Relations of serum aldosterone to cardiac structure: gender-related differences in the Framingham Heart Study. *Hypertension* 2004;**43**:957–962.

74. Clemmer JS, Faulkner JL, Mullen AJ, Butler KR, Hester RL. Sex-specific responses to mineralocorticoid receptor antagonism in hypertensive African American males and females. *Biol Sex Differ* 2019;**10**:24.

75. Merrill M, Sweitzer NK, Lindenfeld J, Kao DP. Sex Differences in Outcomes and Responses to Spironolactone in Heart Failure With Preserved Ejection Fraction: A Secondary Analysis of TOPCAT Trial. *JACC Heart Fail* 2019;**7**:228–238.

76. Pitt B, Remme W, Zannad F, Neaton J, Martinez F, Roniker B, Bittman R, Hurley S, Kleiman J, Gatlin M, Eplerenone Post-Acute Myocardial Infarction Heart Failure Efficacy and Survival Study Investigators. Eplerenone, a selective aldosterone blocker, in patients with left ventricular dysfunction after myocardial infarction. *N Engl J Med* 2003;**348**:1309–1321.

77. Solomon SD, McMurray JJV, Anand IS, Ge J, Lam CSP, Maggioni AP, Martinez F, Packer M, Pfeffer MA, Pieske B, Redfield MM, Rouleau JL, Veldhuisen DJ van, Zannad F, Zile MR, Desai AS, Claggett B, Jhund PS, Boytsov SA, Comin-Colet J, Cleland J, Düngen H-D, Goncalvesova E, Katova T, Kerr Saraiva JF, Lelonek M, Merkely B, Senni M, Shah SJ, Zhou J, Rizkala AR, Gong J, Shi VC, Lefkowitz MP, PARAGON-HF Investigators and Committees. Angiotensin-Neprilysin Inhibition in Heart Failure with Preserved Ejection Fraction. *N Engl J Med* 2019;**381**:1609–1620.

78. McMurray JJV, Jackson AM, Lam CSP, Redfield MM, Anand IS, Ge J, Lefkowitz MP, Maggioni AP, Martinez F, Packer M, Pfeffer MA, Pieske B, Rizkala AR, Sabarwal SV, Shah AM, Shah SJ, Shi VC, Veldhuisen DJ van, Zannad F, Zile MR, Cikes M, Goncalvesova E, Katova T, Kosztin A, Lelonek M, Sweitzer N, Vardeny O, Claggett B, Jhund PS, Solomon SD. Effects of Sacubitril-Valsartan Versus Valsartan in Women Compared With Men With Heart Failure and Preserved Ejection Fraction: Insights From PARAGON-HF. *Circulation* 2020;**141**:338–351.

79. Packer M, Anker SD, Butler J, Filippatos G, Pocock SJ, Carson P, Januzzi J, Verma S, Tsutsui H, Brueckmann M, Jamal W, Kimura K, Schnee J, Zeller C, Cotton D, Bocchi E, Böhm M, Choi D-J, Chopra V, Chuquiure E, Giannetti N, Janssens S, Zhang J, Gonzalez Juanatey JR, Kaul S, Brunner-La Rocca H-P, Merkely B, Nicholls SJ, Perrone S, Pina I, Ponikowski P, Sattar N, Senni M, Seronde M-F, Spinar J, Squire I, Taddei S, Wanner C, Zannad F, EMPEROR-Reduced Trial Investigators. Cardiovascular and Renal Outcomes with Empagliflozin in Heart Failure. *N Engl J Med* 2020;**383**:1413–1424.

80. Rivera FB, Tang VAS, De Luna DV, Lerma EV, Vijayaraghavan K, Kazory A, Shah NS, Volgman AS. Sex differences in cardiovascular outcomes of SGLT-2 inhibitors in heart failure randomized controlled trials: A systematic review and meta-analysis. *Am Heart J Plus* 2023;**26**:100261.

81. Mahmoud AN, Elgendy IY, Saad M, Elgendy AY, Barakat AF, Mentias A, Abuzaid A, Bavry AA. Does Gender Influence the Cardiovascular Benefits Observed with Sodium Glucose Co-Transporter-2 (SGLT-2) Inhibitors? A Meta-Regression Analysis. *Cardiol Ther* 2017;**6**:129–132.

82. Cangemi R, Romiti GF, Campolongo G, Ruscio E, Sciomer S, Gianfrilli D, Raparelli V. Gender related differences in treatment and response to statins in primary and secondary cardiovascular prevention: The never-ending debate. *Pharmacol Res* 2017;**117**:148–155.

83. Nanna MG, Abdullah A, Mortensen MB, Navar AM. Primary prevention statin therapy in older adults. *Curr Opin Cardiol* 2023;**38**:11–20.

84. Culver AL, Ockene IS, Balasubramanian R, Olendzki BC, Sepavich DM, Wactawski-Wende J, Manson JE, Qiao Y, Liu S, Merriam PA, Rahilly-Tierny C, Thomas F, Berger JS, Ockene JK, Curb JD, Ma Y. Statin use and risk of diabetes mellitus in postmenopausal women in the Women’s Health Initiative. *Arch Intern Med* 2012;**172**:144–152.

85. Virani SS, Woodard LD, Ramsey DJ, Urech TH, Akeroyd JM, Shah T, Deswal A, Bozkurt B, Ballantyne CM, Petersen LA. Gender disparities in evidence-based statin therapy in patients with cardiovascular disease. *Am J Cardiol* 2015;**115**:21–26.

86. Nanna MG, Wang TY, Xiang Q, Goldberg AC, Robinson JG, Roger VL, Virani SS, Wilson PWF, Louie MJ, Koren A, Li Z, Peterson ED, Navar AM. Sex Differences in the Use of Statins in Community Practice. *Circ Cardiovasc Qual Outcomes* 2019;**12**:e005562.

87. Newman CB, Preiss D, Tobert JA, Jacobson TA, Page RL, Goldstein LB, Chin C, Tannock LR, Miller M, Raghuveer G, Duell PB, Brinton EA, Pollak A, Braun LT, Welty FK, American Heart Association Clinical Lipidology, Lipoprotein, Metabolism and Thrombosis Committee, a Joint Committee of the Council on Atherosclerosis, Thrombosis and Vascular Biology and Council on Lifestyle and Cardiometabolic Health; Council on Cardiovascular Disease in the Young; Council on Clinical Cardiology; and Stroke Council. Statin Safety and Associated Adverse Events: A Scientific Statement From the American Heart Association. *Arterioscler Thromb Vasc Biol* 2019;**39**:e38–e81.

88. Kent DM, Price LL, Ringleb P, Hill MD, Selker HP. Sex-based differences in response to recombinant tissue plasminogen activator in acute ischemic stroke: a pooled analysis of randomized clinical trials. *Stroke* 2005;**36**:62–65.

89. Mehilli J, Ndrepepa G, Kastrati A, Neumann F-J, Berg J ten, Bruskina O, Dotzer F, Seyfarth M, Pache J, Kufner S, Dirschinger J, Berger PB, Schömig A. Sex and effect of abciximab in patients with acute coronary syndromes treated with percutaneous coronary interventions: results from Intracoronary Stenting and Antithrombotic Regimen: Rapid Early Action for Coronary Treatment 2 trial. *Am Heart J* 2007;**154**:158.e1-7.

90. Carland C, Hansra B, Parsons C, Lyubarova R, Khandelwal A. Adequate enrollment of women in cardiovascular drug trials and the need for sex-specific assessment and reporting. *Am Heart J Plus* 2022;**17**:100155.

91. White HD, Barbash GI, Modan M, Simes J, Diaz R, Hampton JR, Heikkilä J, Kristinsson A, Moulopoulos S, Paolasso EA. After correcting for worse baseline characteristics, women treated with thrombolytic therapy for acute myocardial infarction have the same mortality and morbidity as men except for a higher incidence of hemorrhagic stroke. The Investigators of the International Tissue Plasminogen Activator/Streptokinase Mortality Study. *Circulation* 1993;**88**:2097–2103.

92. Weaver WD, White HD, Wilcox RG, Aylward PE, Morris D, Guerci A, Ohman EM, Barbash GI, Betriu A, Sadowski Z, Topol EJ, Califf RM. Comparisons of characteristics and outcomes among women and men with acute myocardial infarction treated with thrombolytic therapy. GUSTO-I investigators. *JAMA* 1996;**275**:777–782.

93. Woodfield SL, Lundergan CF, Reiner JS, Thompson MA, Rohrbeck SC, Deychak Y, Smith JO, Burton JR, McCarthy WF, Califf RM, White HD, Weaver WD, Topol EJ, Ross AM. Gender and acute myocardial infarction: is there a different response to thrombolysis? *J Am Coll Cardiol* 1997;**29**:35–42.

94. Soldin OP, Mattison DR. Sex differences in pharmacokinetics and pharmacodynamics. *Clin Pharmacokinet* 2009;**48**:143–157.

95. Garcia D, Regan S, Crowther M, Hughes RA, Hylek EM. Warfarin maintenance dosing patterns in clinical practice: implications for safer anticoagulation in the elderly population. *Chest* 2005;**127**:2049–2056.

96. Whitley HP, Fermo JD, Chumney EC, Brzezinski WA. Effect of patient-specific factors on weekly warfarin dose. *Ther Clin Risk Manag* 2007;**3**:499–504.

97. Fang MC, Singer DE, Chang Y, Hylek EM, Henault LE, Jensvold NG, Go AS. Gender differences in the risk of ischemic stroke and peripheral embolism in atrial fibrillation: the AnTicoagulation and Risk factors In Atrial fibrillation (ATRIA) study. *Circulation* 2005;**112**:1687–1691.

98. Pancholy SB, Sharma PS, Pancholy DS, Patel TM, Callans DJ, Marchlinski FE. Meta-analysis of gender differences in residual stroke risk and major bleeding in patients with nonvalvular atrial fibrillation treated with oral anticoagulants. *Am J Cardiol* 2014;**113**:485–490.

99. Shah MR, Granger CB, Bart BA, McMurray JJ, Petrie MC, Michelson EL, Tudor GE, Swedberg K, Stevenson LW, Califf RM, Pfeffer MA. Sex-related differences in the use and adverse effects of angiotensin-converting enzyme inhibitors in heart failure: the study of patients intolerant of converting enzyme inhibitors registry. *Am J Med* 2000;**109**:489–492.

100. Vukadinović D, Vukadinović AN, Lavall D, Laufs U, Wagenpfeil S, Böhm M. Rate of Cough During Treatment With Angiotensin-Converting Enzyme Inhibitors: A Meta-Analysis of Randomized Placebo-Controlled Trials. *Clin Pharmacol Ther* 2019;**105**:652–660.

101. Borghi C, Cicero AF, Agnoletti D, Fiorini G. Pathophysiology of cough with angiotensin-converting enzyme inhibitors: How to explain within-class differences? *Eur J Intern Med* 2023;**110**:10–15.

102. Hudson M, Rahme E, Behlouli H, Sheppard R, Pilote L. Sex differences in the effectiveness of angiotensin receptor blockers and angiotensin converting enzyme inhibitors in patients with congestive heart failure--a population study. *Eur J Heart Fail* 2007;**9**:602–609.

103. Roden DM. Predicting drug-induced QT prolongation and torsades de pointes. *J Physiol* 2016;**594**:2459–2468.

104. Yap YG, Camm AJ. Drug induced QT prolongation and torsades de pointes. *Heart* 2003;**89**:1363–1372.

105. Vedove CD, Del Giglio M, Schena D, Girolomoni G. Drug-induced lupus erythematosus. *Arch Dermatol Res* 2009;**301**:99–105.

106. Moscucci M, Fox K a. A, Cannon CP, Klein W, López-Sendón J, Montalescot G, White K, Goldberg RJ. Predictors of major bleeding in acute coronary syndromes: the Global Registry of Acute Coronary Events (GRACE). *Eur Heart J* 2003;**24**:1815–1823.

107. Warkentin TE, Sheppard J-AI, Sigouin CS, Kohlmann T, Eichler P, Greinacher A. Gender imbalance and risk factor interactions in heparin-induced thrombocytopenia. *Blood* 2006;**108**:2937–2941.

108. Alexander KP, Chen AY, Newby LK, Schwartz JB, Redberg RF, Hochman JS, Roe MT, Gibler WB, Ohman EM, Peterson ED, CRUSADE (Can Rapid risk stratification of Unstable angina patients Suppress ADverse outcomes with Early implementation of the ACC/AHA guidelines) Investigators. Sex differences in major bleeding with glycoprotein IIb/IIIa inhibitors: results from the CRUSADE (Can Rapid risk stratification of Unstable angina patients Suppress ADverse outcomes with Early implementation of the ACC/AHA guidelines) initiative. *Circulation* 2006;**114**:1380–1387.

109. Boersma E, Harrington RA, Moliterno DJ, White H, Théroux P, Van de Werf F, Torbal A de, Armstrong PW, Wallentin LC, Wilcox RG, Simes J, Califf RM, Topol EJ, Simoons ML. Platelet glycoprotein IIb/IIIa inhibitors in acute coronary syndromes: a meta-analysis of all major randomised clinical trials. *Lancet* 2002;**359**:189–198.

110. Lansky AJ, Mehran R, Cristea E, Parise H, Feit F, Ohman EM, White HD, Alexander KP, Bertrand ME, Desmet W, Hamon M, Stone GW. Impact of gender and antithrombin strategy on early and late clinical outcomes in patients with non-ST-elevation acute coronary syndromes (from the ACUITY trial). *Am J Cardiol* 2009;**103**:1196–1203.

111. O’Donoghue ML, Bhatt DL, Stone GW, Steg PG, Gibson CM, Hamm CW, Price MJ, Prats J, Liu T, Deliargyris EN, Mahaffey KW, White HD, Harrington RA, CHAMPION PHOENIX Investigators. Efficacy and Safety of Cangrelor in Women Versus Men During Percutaneous Coronary Intervention: Insights From the Cangrelor versus Standard Therapy to Achieve Optimal Management of Platelet Inhibition (CHAMPION PHOENIX) Trial. *Circulation* 2016;**133**:248–255.

112. Alharbi FF, Kholod AAV, Souverein PC, Meyboom RH, Groot MCH de, Boer A de, Klungel OH. The impact of age and sex on the reporting of cough and angioedema with renin-angiotensin system inhibitors: a case/noncase study in VigiBase. *Fundam Clin Pharmacol* 2017;**31**:676–684.

113. Mauer AC, Khazanov NA, Levenkova N, Tian S, Barbour EM, Khalida C, Tobin JN, Coller BS. Impact of sex, age, race, ethnicity and aspirin use on bleeding symptoms in healthy adults. *J Thromb Haemost* 2011;**9**:100–108.

114. Ridker PM, Cook NR, Lee I-M, Gordon D, Gaziano JM, Manson JE, Hennekens CH, Buring JE. A randomized trial of low-dose aspirin in the primary prevention of cardiovascular disease in women. *N Engl J Med* 2005;**352**:1293–1304.

115. Neutel CI, Maxwell CJ, Appel WC. Differences between males and females in risk of NSAID-related severe gastrointestinal events. *Pharmacoepidemiol Drug Saf* 1999;**8**:501–507.

116. Hansen JM, Hallas J, Lauritsen JM, Bytzer P. Non-steroidal anti-inflammatory drugs and ulcer complications: a risk factor analysis for clinical decision-making. *Scand J Gastroenterol* 1996;**31**:126–130.

117. Hendriksen LC, Omes-Smit G, Koch BCP, Ikram MA, Stricker BH, Visser LE. Sex-Based Difference in the Effect of Metoprolol on Heart Rate and Bradycardia in a Population-Based Setting. *J Pers Med* 2022;**12**:870.

118. Messerli FH. Vasodilatory edema: a common side effect of antihypertensive therapy. *Am J Hypertens* 2001;**14**:978–979.

119. McDonagh TA, Metra M, Adamo M, Gardner RS, Baumbach A, Böhm M, Burri H, Butler J, Čelutkienė J, Chioncel O, Cleland JGF, Coats AJS, Crespo-Leiro MG, Farmakis D, Gilard M, Heymans S, Hoes AW, Jaarsma T, Jankowska EA, Lainscak M, Lam CSP, Lyon AR, McMurray JJV, Mebazaa A, Mindham R, Muneretto C, Francesco Piepoli M, Price S, Rosano GMC, Ruschitzka F, Kathrine Skibelund A, ESC Scientific Document Group. 2021 ESC Guidelines for the diagnosis and treatment of acute and chronic heart failure. *Eur Heart J* 2021;**42**:3599–3726.

120. Bots SH, Schreuder MM, Roeters van Lennep JE, Watson S, Puijenbroek E van, Onland-Moret NC, Ruijter HM den. Sex Differences in Reported Adverse Drug Reactions to Angiotensin-Converting Enzyme Inhibitors. *JAMA Netw Open* 2022;**5**:e228224.

121. Barber J, McKeever TM, McDowell SE, Clayton JA, Ferner RE, Gordon RD, Stowasser M, O’Shaughnessy KM, Hall IP, Glover M. A systematic review and meta-analysis of thiazide-induced hyponatraemia: time to reconsider electrolyte monitoring regimens after thiazide initiation? *Br J Clin Pharmacol* 2015;**79**:566–577.

122. Hendriksen LC, Mouissie MS, Herings RMC, Linden PD van der, Visser LE. Women have a higher risk of hospital admission associated with hyponatremia than men while using diuretics. *Front Pharmacol* 2024;**15**:1409271.

123. Igho Pemu P, Ofili E. Hypertension in women: part I. *J Clin Hypertens (Greenwich)* 2008;**10**:406–410.

124. Gosselin L, Vilcu A-M, Souty C, Steichen O, Launay T, Conte C, Saint-Salvi B, Turbelin C, Sarazin M, Blanchon T, Hanslik T, Lapeyre-Mestre M, Rossignol L. Prevalence and bleeding risk associated with the concomitant use of direct oral anticoagulants and antiarrhythmic drugs in patients with atrial fibrillation, based on the French healthcare insurance database. *Eur J Clin Pharmacol* 2023;**79**:937–945.

125. Rubin RL. Drug-induced lupus. *Expert Opin Drug Saf* 2015;**14**:361–378.

126. Vaglio A, Grayson PC, Fenaroli P, Gianfreda D, Boccaletti V, Ghiggeri GM, Moroni G. Drug-induced lupus: Traditional and new concepts. *Autoimmun Rev* 2018;**17**:912–918.

127. Bots SH, Groepenhoff F, Eikendal ALM, Tannenbaum C, Rochon PA, Regitz-Zagrosek V, Miller VM, Day D, Asselbergs FW, Ruijter HM den. Adverse Drug Reactions to Guideline-Recommended Heart Failure Drugs in Women: A Systematic Review of the Literature. *JACC Heart Fail* 2019;**7**:258–266.

128. Bots SH, Groepenhoff F, Eikendal ALM, Tannenbaum C, Rochon PA, Regitz-Zagrosek V, Miller VM, Day D, Asselbergs FW, Ruijter HM den. Adverse Drug Reactions to Guideline-Recommended Heart Failure Drugs in Women: A Systematic Review of the Literature. *JACC Heart Fail* 2019;**7**:258–266.

129. Rossignol P, Cleland JGF, Bhandari S, Tala S, Gustafsson F, Fay R, Lamiral Z, Dobre D, Pitt B, Zannad F. Determinants and consequences of renal function variations with aldosterone blocker therapy in heart failure patients after myocardial infarction: insights from the Eplerenone Post-Acute Myocardial Infarction Heart Failure Efficacy and Survival Study. *Circulation* 2012;**125**:271–279.

130. Yancy CW, Jessup M, Bozkurt B, Butler J, Casey DE, Colvin MM, Drazner MH, Filippatos GS, Fonarow GC, Givertz MM, Hollenberg SM, Lindenfeld J, Masoudi FA, McBride PE, Peterson PN, Stevenson LW, Westlake C. 2017 ACC/AHA/HFSA Focused Update of the 2013 ACCF/AHA Guideline for the Management of Heart Failure: A Report of the American College of Cardiology/American Heart Association Task Force on Clinical Practice Guidelines and the Heart Failure Society of America. *Circulation* 2017;**136**:e137–e161.

131. Nuechterlein K, AlTurki A, Ni J, Martínez-Sellés M, Martens P, Russo V, Backelin CN, Huynh T. Real-World Safety of Sacubitril/Valsartan in Women and Men With Heart Failure and Reduced Ejection Fraction: A Meta-analysis. *CJC Open* 2021;**3**:S202–S208.

132. Kostis WJ, Shetty M, Chowdhury YS, Kostis JB. ACE Inhibitor-Induced Angioedema: a Review. *Curr Hypertens Rep* 2018;**20**:55.

133. Fadini GP, Bonora BM, Avogaro A. SGLT2 inhibitors and diabetic ketoacidosis: data from the FDA Adverse Event Reporting System. *Diabetologia* 2017;**60**:1385–1389.

134. McGill JB, Subramanian S. Safety of Sodium-Glucose Co-Transporter 2 Inhibitors. *Am J Cardiol* 2019;**124 Suppl 1**:S45–S52.

135. Raparelli V, Pannitteri G, Todisco T, Toriello F, Napoleone L, Manfredini R, Basili S. Treatment and Response to Statins: Gender-related Differences. *Curr Med Chem* 2017;**24**:2628–2638.

136. Thompson PD, Panza G, Zaleski A, Taylor B. Statin-Associated Side Effects. *J Am Coll Cardiol* 2016;**67**:2395–2410.

137. Bang CN, Okin PM. Statin treatment, new-onset diabetes, and other adverse effects: a systematic review. *Curr Cardiol Rep* 2014;**16**:461.

138. Werf C van der, Kannankeril PJ, Sacher F, Krahn AD, Viskin S, Leenhardt A, Shimizu W, Sumitomo N, Fish FA, Bhuiyan ZA, Willems AR, Veen MJ van der, Watanabe H, Laborderie J, Haïssaguerre M, Knollmann BC, Wilde AAM. Flecainide therapy reduces exercise-induced ventricular arrhythmias in patients with catecholaminergic polymorphic ventricular tachycardia. *J Am Coll Cardiol* 2011;**57**:2244–2254.

139. Reynolds HR, Farkouh ME, Lincoff AM, Hsu A, Swahn E, Sadowski ZP, White JA, Topol EJ, Hochman JS, GUSTO V Investigators. Impact of female sex on death and bleeding after fibrinolytic treatment of myocardial infarction in GUSTO V. *Arch Intern Med* 2007;**167**:2054–2060.

140. Van de Werf F, Barron HV, Armstrong PW, Granger CB, Berioli S, Barbash G, Pehrsson K, Verheugt FW, Meyer J, Betriu A, Califf RM, Li X, Fox NL, ASSENT-2 Investigators. Assessment of the Safety and Efficacy of a New Thrombolytic. Incidence and predictors of bleeding events after fibrinolytic therapy with fibrin-specific agents: a comparison of TNK-tPA and rt-PA. *Eur Heart J* 2001;**22**:2253–2261.

141. Meer FJ van der, Rosendaal FR, Vandenbroucke JP, Briët E. Bleeding complications in oral anticoagulant therapy. An analysis of risk factors. *Arch Intern Med* 1993;**153**:1557–1562.

142. Pengo V, Legnani C, Noventa F, Palareti G, ISCOAT Study Group.(Italian Study on Complications of Oral Anticoagulant Therapy). Oral anticoagulant therapy in patients with nonrheumatic atrial fibrillation and risk of bleeding. A Multicenter Inception Cohort Study. *Thromb Haemost* 2001;**85**:418–422.

143. Johnson SM, Karvonen CA, Phelps CL, Nader S, Sanborn BM. Assessment of analysis by gender in the Cochrane reviews as related to treatment of cardiovascular disease. *J Womens Health (Larchmt)* 2003;**12**:449–457.

144. Kim ESH, Carrigan TP, Menon V. Enrollment of women in National Heart, Lung, and Blood Institute-funded cardiovascular randomized controlled trials fails to meet current federal mandates for inclusion. *J Am Coll Cardiol* 2008;**52**:672–673.

145. Geller SE, Koch A, Pellettieri B, Carnes M. Inclusion, analysis, and reporting of sex and race/ethnicity in clinical trials: have we made progress? *J Womens Health (Larchmt)* 2011;**20**:315–320.

146. Sardar MR, Badri M, Prince CT, Seltzer J, Kowey PR. Underrepresentation of women, elderly patients, and racial minorities in the randomized trials used for cardiovascular guidelines. *JAMA Intern Med* 2014;**174**:1868–1870.

147. Tsang W, Alter DA, Wijeysundera HC, Zhang T, Ko DT. The impact of cardiovascular disease prevalence on women’s enrollment in landmark randomized cardiovascular trials: a systematic review. *J Gen Intern Med* 2012;**27**:93–98.

148. Crespo-Leiro MG, Anker SD, Maggioni AP, Coats AJ, Filippatos G, Ruschitzka F, Ferrari R, Piepoli MF, Delgado Jimenez JF, Metra M, Fonseca C, Hradec J, Amir O, Logeart D, Dahlström U, Merkely B, Drozdz J, Goncalvesova E, Hassanein M, Chioncel O, Lainscak M, Seferovic PM, Tousoulis D, Kavoliuniene A, Fruhwald F, Fazlibegovic E, Temizhan A, Gatzov P, Erglis A, Laroche C, Mebazaa A, Heart Failure Association (HFA) of the European Society of Cardiology (ESC). European Society of Cardiology Heart Failure Long-Term Registry (ESC-HF-LT): 1-year follow-up outcomes and differences across regions. *Eur J Heart Fail* 2016;**18**:613–625.

149. Pressler SJ. Women With Heart Failure Are Disproportionately Studied as Compared With Prevalence: A Review of Published Studies from 2013. *J Cardiovasc Nurs* 2016;**31**:84–88.

150. Nguyen QD, Peters E, Wassef A, Desmarais P, Rémillard-Labrosse D, Tremblay-Gravel M. Evolution of Age and Female Representation in the Most-Cited Randomized Controlled Trials of Cardiology of the Last 20 Years. *Circ Cardiovasc Qual Outcomes* 2018;**11**:e004713.

151. Hartman RJG, Huisman SE, Ruijter HM den. Sex differences in cardiovascular epigenetics-a systematic review. *Biol Sex Differ* 2018;**9**:19.

152. Scott PE, Unger EF, Jenkins MR, Southworth MR, McDowell T-Y, Geller RJ, Elahi M, Temple RJ, Woodcock J. Participation of Women in Clinical Trials Supporting FDA Approval of Cardiovascular Drugs. *J Am Coll Cardiol* 2018;**71**:1960–1969.

153. Tahhan AS, Vaduganathan M, Greene SJ, Fonarow GC, Fiuzat M, Jessup M, Lindenfeld J, O’Connor CM, Butler J. Enrollment of Older Patients, Women, and Racial and Ethnic Minorities in Contemporary Heart Failure Clinical Trials: A Systematic Review. *JAMA Cardiol* 2018;**3**:1011–1019.

154. Gong IY, Tan NS, Ali SH, Lebovic G, Mamdani M, Goodman SG, Ko DT, Laupacis A, Yan AT. Temporal Trends of Women Enrollment in Major Cardiovascular Randomized Clinical Trials. *Can J Cardiol* 2019;**35**:653–660.

155. Sepehrvand N, Alemayehu W, Das D, Gupta AK, Gouda P, Ghimire A, Du AX, Hatami S, Babadagli HE, Verma S, Kashour Z, Ezekowitz JA. Trends in the Explanatory or Pragmatic Nature of Cardiovascular Clinical Trials Over 2 Decades. *JAMA Cardiol* 2019;**4**:1122–1128.

156. Khan MS, Shahid I, Siddiqi TJ, Khan SU, Warraich HJ, Greene SJ, Butler J, Michos ED. Ten-Year Trends in Enrollment of Women and Minorities in Pivotal Trials Supporting Recent US Food and Drug Administration Approval of Novel Cardiometabolic Drugs. *J Am Heart Assoc* 2020;**9**:e015594.

157. Jin X, Chandramouli C, Allocco B, Gong E, Lam CSP, Yan LL. Women’s Participation in Cardiovascular Clinical Trials From 2010 to 2017. *Circulation* 2020;**141**:540–548.

158. Khan SU, Khan MZ, Raghu Subramanian C, Riaz H, Khan MU, Lone AN, Khan MS, Benson E-M, Alkhouli M, Blaha MJ, Blumenthal RS, Gulati M, Michos ED. Participation of Women and Older Participants in Randomized Clinical Trials of Lipid-Lowering Therapies: A Systematic Review. *JAMA Netw Open* 2020;**3**:e205202.

159. Ray KK, Wright RS, Kallend D, Koenig W, Leiter LA, Raal FJ, Bisch JA, Richardson T, Jaros M, Wijngaard PLJ, Kastelein JJP, ORION-10 and ORION-11 Investigators. Two Phase 3 Trials of Inclisiran in Patients with Elevated LDL Cholesterol. *N Engl J Med* 2020;**382**:1507–1519.

160. Whitelaw S, Sullivan K, Eliya Y, Alruwayeh M, Thabane L, Yancy CW, Mehran R, Mamas MA, Van Spall HGC. Trial characteristics associated with under-enrolment of females in randomized controlled trials of heart failure with reduced ejection fraction: a systematic review. *Eur J Heart Fail* 2021;**23**:15–24.

161. Carcel C, Harris K, Peters SAE, Sandset EC, Balicki G, Bushnell CD, Howard VJ, Reeves MJ, Anderson CS, Kelly PJ, Woodward M. Representation of Women in Stroke Clinical Trials: A Review of 281 Trials Involving More Than 500,000 Participants. *Neurology* 2021;**97**:e1768–e1774.

162. Au M, Whitelaw S, Khan MS, Mamas MA, Mbuagbaw L, Mulvagh SL, Voors AA, Van Spall HGC. A Systematic Review of Sex-Specific Reporting in Heart Failure Clinical Trials: Trial Flow and Results. *JACC Adv* 2022;**1**:100079.

163. Mayor JM, Preventza O, McGinigle K, Mills JL, Montero-Baker M, Gilani R, Pallister Z, Chung J. Persistent under-representation of female patients in United States trials of common vascular diseases from 2008 to 2020. *J Vasc Surg* 2022;**75**:30–36.

164. Mohseni-Alsalhi Z, Vesseur MAM, Wilmes N, Laven SAJS, Meijs DAM, Luik EM van, Vaes EWP, Dikovec CJR, Wiesenberg J, Almutairi MF, Janssen EBNJ, Haas S de, Spaanderman MEA, Ghossein-Doha C. The Representation of Females in Studies on Antihypertensive Medication over the Years: A Scoping Review. *Biomedicines* 2023;**11**:1435.

165. Holtzman JN, Kaur G, Power JE, Barkhordarian M, Mares A, Goyal A, Gulati M. Underrepresentation of Women in Late-Breaking Cardiovascular Clinical Trials. *J Womens Health (Larchmt)* 2023;**32**:635–640.

166. Byrne RA, Rossello X, Coughlan JJ, Barbato E, Berry C, Chieffo A, Claeys MJ, Dan G-A, Dweck MR, Galbraith M, Gilard M, Hinterbuchner L, Jankowska EA, Jüni P, Kimura T, Kunadian V, Leosdottir M, Lorusso R, Pedretti RFE, Rigopoulos AG, Rubini Gimenez M, Thiele H, Vranckx P, Wassmann S, Wenger NK, Ibanez B, ESC Scientific Document Group. 2023 ESC Guidelines for the management of acute coronary syndromes. *Eur Heart J* 2023;**44**:3720–3826.

167. Bastian-Pétrel K, Rohmann JL, Oertelt-Prigione S, Piccininni M, Gayraud K, Kelly-Irving M, Bajos N. Sex and gender bias in chronic coronary syndromes research: analysis of studies used to inform the 2019 European Society of Cardiology guidelines. *Lancet Reg Health Eur* 2024;**45**:101041.

168. Gupta R, Umeh C, Mohta T, Vaidya A, Wolfson A, Nattiv J, Bhatia H, Kaur G, Dhawan R, Darji P, Eghreriniovo B, Sanwo E, Hotwani P, Mahdavian P, Kumar S, Tiwari B. Representation of women and racial minorities in SGLT2 inhibitors and heart failure clinical trials. *Int J Cardiol Heart Vasc* 2024;**55**:101539.

169. Doull M, Runnels VE, Tudiver S, Boscoe M. Appraising the evidence: applying sex- and gender-based analysis (SGBA) to Cochrane systematic reviews on cardiovascular diseases. *J Womens Health (Larchmt)* 2010;**19**:997–1003.

170. Phillips SP, Hamberg K. Doubly blind: a systematic review of gender in randomised controlled trials. *Glob Health Action* 2016;**9**:29597.

171. Welch V, Doull M, Yoganathan M, Jull J, Boscoe M, Coen SE, Marshall Z, Pardo JP, Pederson A, Petkovic J, Puil L, Quinlan L, Shea B, Rader T, Runnels V, Tudiver S. Reporting of sex and gender in randomized controlled trials in Canada: a cross-sectional methods study. *Res Integr Peer Rev* 2017;**2**:15.

172. Petkovic J, Trawin J, Dewidar O, Yoganathan M, Tugwell P, Welch V. Sex/gender reporting and analysis in Campbell and Cochrane systematic reviews: a cross-sectional methods study. *Syst Rev* 2018;**7**:113.

173. Bots SH, Ruijter HM den. Recommended Heart Failure Medications and Adverse Drug Reactions in Women. *Circulation* 2019;**139**:1469–1471.

174. Khan MS, Khan MAA, Irfan S, Siddiqi TJ, Greene SJ, Anker SD, Sreenivasan J, Friede T, Tahhan AS, Vaduganathan M, Fonarow GC, Butler J. Reporting and interpretation of subgroup analyses in heart failure randomized controlled trials. *ESC Heart Fail* 2021;**8**:26–36.

175. Strocchi E, Baffoni L, Bragagni A, Soldati M, >Borghi C. The attention on gender in studies on the treatment of hypertension: was it enough? *Ital J Gender-Specific Med* 2021;**7**:94–101.

176. Schreuder MM, Boersma E, Kavousi M, Visser LE, Roos-Hesselink JW, Versmissen J, Roeters van Lennep JE. Reporting of sex-specific outcomes in trials of interventions for cardiovascular disease: Has there been progress? *Maturitas* 2021;**144**:1–3.

177. Mohseni-Alsalhi Z, Vesseur MAM, Wilmes N, Laven SAJS, Meijs DAM, Luik EM van, Vaes EWP, Dikovec CJR, Wiesenberg J, Almutairi MF, Janssen EBNJ, Haas S de, Spaanderman MEA, Ghossein-Doha C. The Representation of Females in Studies on Antihypertensive Medication over the Years: A Scoping Review. *Biomedicines* 2023;**11**:1435.

178. Granton D, Rodrigues M, Raparelli V, Honarmand K, Agarwal A, Friedrich JO, Perna B, Spaggiari R, Fortunato V, Risdonne G, Kho M, VanderKaay S, Chaudhuri D, Gomez-Builes C, D’Aragon F, Wiseman D, Lau VI, Lin C, Reid J, Trivedi V, Prakash V, Belley-Cote E, Al Mandhari M, Thabane L, Pilote L, Burns KEA. Sex and gender-based analysis and diversity metric reporting in acute care trials published in high-impact journals: a systematic review. *BMJ Open* 2024;**14**:e081118.

179. Gulamhusein N, Turino Miranda K, Dumanski SM, González Bedat MC, Ulasi I, Conjeevaram A, Ahmed SB. Sex- and Gender-Based Reporting in Antihypertensive Medication Literature Informing Hypertension Guidelines. *J Am Heart Assoc* 2024;**13**:e030613.

180. Wang W, Ma Y, Huang Y, Chen H. Generalizability analysis for clinical trials: a simulation study. *Stat Med* 2017;**36**:1523–1531.

181. Mattioli AV, Nasi M, Pinti M, Palumbo C. Teaching Gender Differences at Medical School Could Improve the Safety and Efficacy of Personalized Physical Activity Prescription. *Front Cardiovasc Med* 2022;**9**:919257.

182. Mosca L, Benjamin EJ, Berra K, Bezanson JL, Dolor RJ, Lloyd-Jones DM, Newby LK, Piña IL, Roger VL, Shaw LJ, Zhao D, Beckie TM, Bushnell C, D’Armiento J, Kris-Etherton PM, Fang J, Ganiats TG, Gomes AS, Gracia CR, Haan CK, Jackson EA, Judelson DR, Kelepouris E, Lavie CJ, Moore A, Nussmeier NA, Ofili E, Oparil S, Ouyang P, Pinn VW, Sherif K, Smith SC, Sopko G, Chandra-Strobos N, Urbina EM, Vaccarino V, Wenger NK, American Heart Association. Effectiveness-based guidelines for the prevention of cardiovascular disease in women--2011 update: a guideline from the American Heart Association. *J Am Coll Cardiol* 2011;**57**:1404–1423.

183. Wenger NK, Lloyd-Jones DM, Elkind MSV, Fonarow GC, Warner JJ, Alger HM, Cheng S, Kinzy C, Hall JL, Roger VL, American Heart Association. Call to Action for Cardiovascular Disease in Women: Epidemiology, Awareness, Access, and Delivery of Equitable Health Care: A Presidential Advisory From the American Heart Association. *Circulation* 2022;**145**:e1059–e1071.

184. Burgess S, Zaman S, Towns C, Coylewright M, Cader FA. The under-representation of women in cardiovascular clinical trials: State-of-the-art review and ethical considerations. *Am Heart J* 2025;**282**:81–92.

185. Cho L, Vest AR, O’Donoghue ML, Ogunniyi MO, Sarma AA, Denby KJ, Lau ES, Poole JE, Lindley KJ, Mehran R, Cardiovascular Disease in Women Committee Leadership Council. Increasing Participation of Women in Cardiovascular Trials: JACC Council Perspectives. *J Am Coll Cardiol* 2021;**78**:737–751.

186. Wallach JD, Sullivan PG, Trepanowski JF, Steyerberg EW, Ioannidis JPA. Sex based subgroup differences in randomized controlled trials: empirical evidence from Cochrane meta-analyses. *BMJ* 2016;**355**:i5826.

187. Mehta LS, Beckie TM, DeVon HA, Grines CL, Krumholz HM, Johnson MN, Lindley KJ, Vaccarino V, Wang TY, Watson KE, Wenger NK, American Heart Association Cardiovascular Disease in Women and Special Populations Committee of the Council on Clinical Cardiology, Council on Epidemiology and Prevention, Council on Cardiovascular and Stroke Nursing, and Council on Quality of Care and Outcomes Research. Acute Myocardial Infarction in Women: A Scientific Statement From the American Heart Association. *Circulation* 2016;**133**:916–947.

188. Vogel B, Acevedo M, Appelman Y, Bairey Merz CN, Chieffo A, Figtree GA, Guerrero M, Kunadian V, Lam CSP, Maas AHEM, Mihailidou AS, Olszanecka A, Poole JE, Saldarriaga C, Saw J, Zühlke L, Mehran R. The Lancet women and cardiovascular disease Commission: reducing the global burden by 2030. *Lancet* 2021;**397**:2385–2438.

189. Koopman C, Vaartjes I, Heintjes EM, Spiering W, Dis I van, Herings RMC, Bots ML. Persisting gender differences and attenuating age differences in cardiovascular drug use for prevention and treatment of coronary heart disease, 1998-2010. *Eur Heart J* 2013;**34**:3198–3205.

190. Bugiardini R, Yan AT, Yan RT, Fitchett D, Langer A, Manfrini O, Goodman SG, Canadian Acute Coronary Syndrome Registry I and II Investigators. Factors influencing underutilization of evidence-based therapies in women. *Eur Heart J* 2011;**32**:1337–1344.

191. Redfors B, Angerås O, Råmunddal T, Petursson P, Haraldsson I, Dworeck C, Odenstedt J, Ioaness D, Ravn-Fischer A, Wellin P, Sjöland H, Tokgozoglu L, Tygesen H, Frick E, Roupe R, Albertsson P, Omerovic E. Trends in Gender Differences in Cardiac Care and Outcome After Acute Myocardial Infarction in Western Sweden: A Report From the Swedish Web System for Enhancement of Evidence-Based Care in Heart Disease Evaluated According to Recommended Therapies (SWEDEHEART). *J Am Heart Assoc* 2015;**4**:e001995.

192. Blomkalns AL, Chen AY, Hochman JS, Peterson ED, Trynosky K, Diercks DB, Brogan GX, Boden WE, Roe MT, Ohman EM, Gibler WB, Newby LK, CRUSADE Investigators. Gender disparities in the diagnosis and treatment of non-ST-segment elevation acute coronary syndromes: large-scale observations from the CRUSADE (Can Rapid Risk Stratification of Unstable Angina Patients Suppress Adverse Outcomes With Early Implementation of the American College of Cardiology/American Heart Association Guidelines) National Quality Improvement Initiative. *J Am Coll Cardiol* 2005;**45**:832–837.

193. Poon S, Goodman SG, Yan RT, Bugiardini R, Bierman AS, Eagle KA, Johnston N, Huynh T, Grondin FR, Schenck-Gustafsson K, Yan AT. Bridging the gender gap: Insights from a contemporary analysis of sex-related differences in the treatment and outcomes of patients with acute coronary syndromes. *Am Heart J* 2012;**163**:66–73.

194. Jneid H, Fonarow GC, Cannon CP, Hernandez AF, Palacios IF, Maree AO, Wells Q, Bozkurt B, Labresh KA, Liang L, Hong Y, Newby LK, Fletcher G, Peterson E, Wexler L, Get With the Guidelines Steering Committee and Investigators. Sex differences in medical care and early death after acute myocardial infarction. *Circulation* 2008;**118**:2803–2810.

195. Bots SH, Inia JA, Peters SAE. Medication Adherence After Acute Coronary Syndrome in Women Compared With Men: A Systematic Review and Meta-Analysis. *Front Glob Womens Health* 2021;**2**:637398.

196. Cadeddu C, Franconi F, Cassisa L, Campesi I, Pepe A, Cugusi L, Maffei S, Gallina S, Sciomer S, Mercuro G, Working Group of Gender Medicine of Italian Society of Cardiology. Arterial hypertension in the female world: pathophysiology and therapy. *J Cardiovasc Med (Hagerstown)* 2016;**17**:229–236.

197. Ljungman C, Kahan T, Schiöler L, Hjerpe P, Hasselström J, Wettermark B, Boström KB, Manhem K. Gender differences in antihypertensive drug treatment: results from the Swedish Primary Care Cardiovascular Database (SPCCD). *J Am Soc Hypertens* 2014;**8**:882–890.

198. Wallentin F, Wettermark B, Kahan T. Drug treatment of hypertension in Sweden in relation to sex, age, and comorbidity. *J Clin Hypertens (Greenwich)* 2018;**20**:106–114.

199. Muiesan ML, Salvetti M, Rosei CA, Paini A. Gender Differences in Antihypertensive Treatment: Myths or Legends? *High Blood Press Cardiovasc Prev* 2016;**23**:105–113.

200. Deborde T, Amar L, Bobrie G, Postel-Vinay N, Battaglia C, Tache A, Chedid A, Dhib M-M, Chatellier G, Plouin P-F, Burgun A, Azizi M, Jannot A-S. Sex differences in antihypertensive treatment in France among 17 856 patients in a tertiary hypertension unit. *J Hypertens* 2018;**36**:939–946.

201. Thoenes M, Neuberger H-R, Volpe M, Khan BV, Kirch W, Böhm M. Antihypertensive drug therapy and blood pressure control in men and women: an international perspective. *J Hum Hypertens* 2010;**24**:336–344.

202. Van der Niepen P, Verbeelen D. Gender and hypertension management: a sub-analysis of the I-inSYST survey. *Blood Press* 2011;**20**:69–76.

203. Dagres N, Nieuwlaat R, Vardas PE, Andresen D, Lévy S, Cobbe S, Kremastinos DT, Breithardt G, Cokkinos DV, Crijns HJGM. Gender-related differences in presentation, treatment, and outcome of patients with atrial fibrillation in Europe: a report from the Euro Heart Survey on Atrial Fibrillation. *J Am Coll Cardiol* 2007;**49**:572–577.

204. Friberg L, Benson L, Rosenqvist M, Lip GYH. Assessment of female sex as a risk factor in atrial fibrillation in Sweden: nationwide retrospective cohort study. *BMJ* 2012;**344**:e3522.

205. Yong CM, Tremmel JA, Lansberg MG, Fan J, Askari M, Turakhia MP. Sex Differences in Oral Anticoagulation and Outcomes of Stroke and Intracranial Bleeding in Newly Diagnosed Atrial Fibrillation. *J Am Heart Assoc* 2020;**9**:e015689.

206. Thompson LE, Maddox TM, Lei L, Grunwald GK, Bradley SM, Peterson PN, Masoudi FA, Turchin A, Song Y, Doros G, Davis MB, Daugherty SL. Sex Differences in the Use of Oral Anticoagulants for Atrial Fibrillation: A Report From the National Cardiovascular Data Registry (NCDR®) PINNACLE Registry. *J Am Heart Assoc* 2017;**6**:e005801.

207. Mentias A, Nakhla S, Desai MY, Wazni O, Menon V, Kapadia S, Vaughan Sarrazin M. Racial and Sex Disparities in Anticoagulation After Electrical Cardioversion for Atrial Fibrillation and Flutter. *J Am Heart Assoc* 2021;**10**:e021674.

208. Enriquez JR, Pratap P, Zbilut JP, Calvin JE, Volgman AS. Women tolerate drug therapy for coronary artery disease as well as men do, but are treated less frequently with aspirin, beta-blockers, or statins. *Gend Med* 2008;**5**:53–61.

209. Manteuffel M, Williams S, Chen W, Verbrugge RR, Pittman DG, Steinkellner A. Influence of patient sex and gender on medication use, adherence, and prescribing alignment with guidelines. *J Womens Health (Larchmt)* 2014;**23**:112–119.

210. Elgendy IY, Wegermann ZK, Li S, Mahtta D, Grau-Sepulveda M, Smilowitz NR, Gulati M, Garratt KN, Wang TY, Jneid H. Sex Differences in Management and Outcomes of Acute Myocardial Infarction Patients Presenting With Cardiogenic Shock. *JACC Cardiovasc Interv* 2022;**15**:642–652.

211. Avgil Tsadok M, Jackevicius CA, Rahme E, Humphries KH, Pilote L. Sex Differences in Dabigatran Use, Safety, And Effectiveness In a Population-Based Cohort of Patients With Atrial Fibrillation. *Circ Cardiovasc Qual Outcomes* 2015;**8**:593–599.

212. Baumhäkel M, Müller U, Böhm M. Influence of gender of physicians and patients on guideline-recommended treatment of chronic heart failure in a cross-sectional study. *Eur J Heart Fail* 2009;**11**:299–303.

213. Lainščak M, Milinković I, Polovina M, Crespo-Leiro MG, Lund LH, Anker SD, Laroche C, Ferrari R, Coats AJS, McDonagh T, Filippatos G, Maggioni AP, Piepoli MF, Rosano GMC, Ruschitzka F, Simić D, Ašanin M, Eicher J-C, Yilmaz MB, Seferović PM, European Society of Cardiology Heart Failure Long-Term Registry Investigators Group. Sex- and age-related differences in the management and outcomes of chronic heart failure: an analysis of patients from the ESC HFA EORP Heart Failure Long-Term Registry. *Eur J Heart Fail* 2020;**22**:92–102.

214. Norberg H, Pranic V, Bergdahl E, Lindmark K. Differences in medical treatment and clinical characteristics between men and women with heart failure - a single-centre multivariable analysis. *Eur J Clin Pharmacol* 2020;**76**:539–546.

215. Majahalme SK, Baruch L, Aknay N, Goedel-Meinen L, Hofmann M, Hester A, Prescott MF, Feliciano N, Val-HeFT Study Investigators. Comparison of treatment benefit and outcome in women versus men with chronic heart failure (from the Valsartan Heart Failure Trial). *Am J Cardiol* 2005;**95**:529–532.

216. Lenzen MJ, Rosengren A, Scholte op Reimer WJM, Follath F, Boersma E, Simoons ML, Cleland JGF, Komajda M. Management of patients with heart failure in clinical practice: differences between men and women. *Heart* 2008;**94**:e10.

217. Dewan P, Rørth R, Jhund PS, Shen L, Raparelli V, Petrie MC, Abraham WT, Desai AS, Dickstein K, Køber L, Mogensen UM, Packer M, Rouleau JL, Solomon SD, Swedberg K, Zile MR, McMurray JJV. Differential Impact of Heart Failure With Reduced Ejection Fraction on Men and Women. *J Am Coll Cardiol* 2019;**73**:29–40.

218. Russo G, Rea F, Barbati G, Cherubini A, Stellato K, Scagnetto A, Iorio A, Corrao G, Di Lenarda A. Sex-related differences in chronic heart failure: a community-based study. *J Cardiovasc Med (Hagerstown)* 2021;**22**:36–44.

219. Dewan P, Rørth R, Raparelli V, Campbell RT, Shen L, Jhund PS, Petrie MC, Anand IS, Carson PE, Desai AS, Granger CB, Køber L, Komajda M, McKelvie RS, O’Meara E, Pfeffer MA, Pitt B, Solomon SD, Swedberg K, Zile MR, McMurray JJV. Sex-Related Differences in Heart Failure With Preserved Ejection Fraction. *Circ Heart Fail* 2019;**12**:e006539.

220. Motiejūnaitė J, Akiyama E, Cohen-Solal A, Maggioni AP, Mueller C, Choi D-J, Kavoliūnienė A, Čelutkienė J, Parenica J, Lassus J, Kajimoto K, Sato N, Miró Ò, Peacock WF, Matsue Y, Voors AA, Lam CSP, Ezekowitz JA, Ahmed A, Fonarow GC, Gayat E, Regitz-Zagrosek V, Mebazaa A. The association of long-term outcome and biological sex in patients with acute heart failure from different geographic regions. *Eur Heart J* 2020;**41**:1357–1364.

221. Garcia M, Mulvagh SL, Merz CNB, Buring JE, Manson JE. Cardiovascular Disease in Women: Clinical Perspectives. *Circ Res* 2016;**118**:1273–1293.

222. Nanna MG, Wang TY, Xiang Q, Goldberg AC, Robinson JG, Roger VL, Virani SS, Wilson PWF, Louie MJ, Koren A, Li Z, Peterson ED, Navar AM. Sex Differences in the Use of Statins in Community Practice. *Circ Cardiovasc Qual Outcomes* 2019;**12**:e005562.

223. Virani SS, Woodard LD, Ramsey DJ, Urech TH, Akeroyd JM, Shah T, Deswal A, Bozkurt B, Ballantyne CM, Petersen LA. Gender disparities in evidence-based statin therapy in patients with cardiovascular disease. *Am J Cardiol* 2015;**115**:21–26.

224. Iatan I, Akioyamen LE, Ruel I, Guerin A, Hales L, Coutinho T, Brunham LR, Genest J. Sex differences in treatment of familial hypercholesterolaemia: a meta-analysis. *Eur Heart J* 2024;**45**:3231–3250.

225. Muiesan ML, Ambrosioni E, Costa FV, Leonetti G, Pessina AC, Salvetti M, Trimarco B, Volpe M, Pontremoli R, Deferrari G, Rosei EA. Sex differences in hypertension-related renal and cardiovascular diseases in Italy: the I-DEMAND study. *J Hypertens* 2012;**30**:2378–2386.

226. Baczek VL, Chen WT, Kluger J, Coleman CI. Predictors of warfarin use in atrial fibrillation in the United States: a systematic review and meta-analysis. *BMC Fam Pract* 2012;**13**:5.

227. Zhao M, Woodward M, Vaartjes I, Millett ERC, Klipstein-Grobusch K, Hyun K, Carcel C, Peters SAE. Sex Differences in Cardiovascular Medication Prescription in Primary Care: A Systematic Review and Meta-Analysis. *J Am Heart Assoc* 2020;**9**:e014742.

228. Isakadze N, Mehta PK, Law K, Dolan M, Lundberg GP. Addressing the Gap in Physician Preparedness To Assess Cardiovascular Risk in Women: a Comprehensive Approach to Cardiovascular Risk Assessment in Women. *Curr Treat Options Cardiovasc Med* 2019;**21**:47.

229. Vogel B, Baber U, Cohen DJ, Sartori S, Sharma SK, Angiolillo DJ, Farhan S, Goel R, Zhang Z, Briguori C, Collier T, Dangas G, Dudek D, Escaned J, Gil R, Han Y-L, Kaul U, Kornowski R, Krucoff MW, Kunadian V, Mehta SR, Moliterno D, Ohman EM, Sardella G, Witzenbichler B, Gibson CM, Pocock S, Huber K, Mehran R. Sex Differences Among Patients With High Risk Receiving Ticagrelor With or Without Aspirin After Percutaneous Coronary Intervention: A Subgroup Analysis of the TWILIGHT Randomized Clinical Trial. *JAMA Cardiol* 2021;**6**:1032–1041.

230. Zannad F, Berwanger O, Corda S, Cowie MR, Gamra H, Gibson CM, Goncalves A, Hucko T, Khunti K, Kostrubiec M, Kraus BJ, Linde C, Lüscher TF, Mafham M, Mindham R, Ortega RF, Prescott E, Thabane L, Yancy C, Ziegler A, Van Spall HGC. How to make cardiology clinical trials more inclusive. *Nat Med* 2024;**30**:2745–2755.

231. Van Spall HGC, Lala A, Deering TF, Casadei B, Zannad F, Kaul P, Mehran R, Pearson GD, Shah MR, Gulati M, Grines C, Volgman AS, Revkin JH, Piña I, Lam CSP, Hochman JS, Simon T, Walsh MN, Bozkurt B, Global CardioVascular Clinical Trialists (CVCT) Forum and Women As One Scientific Expert Panel. Ending Gender Inequality in Cardiovascular Clinical Trial Leadership: JACC Review Topic of the Week. *J Am Coll Cardiol* 2021;**77**:2960–2972.

232. Filbey L, Zhu JW, D’Angelo F, Thabane L, Khan MS, Lewis E, Patel MR, Powell-Wiley T, Miranda JJ, Zuhlke L, Butler J, Zannad F, Van Spall HGC. Improving representativeness in trials: a call to action from the Global Cardiovascular Clinical Trialists Forum. *Eur Heart J* 2023;**44**:921–930.

233. O’Neill J, Tabish H, Welch V, Petticrew M, Pottie K, Clarke M, Evans T, Pardo Pardo J, Waters E, White H, Tugwell P. Applying an equity lens to interventions: using PROGRESS ensures consideration of socially stratifying factors to illuminate inequities in health. *J Clin Epidemiol* 2014;**67**:56–64.

234. Heidari S, Babor TF, De Castro P, Tort S, Curno M. Sex and Gender Equity in Research: rationale for the SAGER guidelines and recommended use. *Res Integr Peer Rev* 2016;**1**:2.

235. Reza N, Nayak A, Lewsey SC, DeFilippis EM. Representation matters: a call for inclusivity and equity in heart failure clinical trials. *Eur Heart J Suppl* 2022;**24**:L45–L48.
